# Sulfonamide Inhibitors of Human Carbonic Anhydrases Designed through a Three-Tails Approach: Improving Ligand/Isoform Matching and Selectivity of Action

**DOI:** 10.1021/acs.jmedchem.0c00733

**Published:** 2020-06-10

**Authors:** Alessandro Bonardi, Alessio Nocentini, Silvia Bua, Jacob Combs, Carrie Lomelino, Jacob Andring, Laura Lucarini, Silvia Sgambellone, Emanuela Masini, Robert McKenna, Paola Gratteri, Claudiu T. Supuran

**Affiliations:** † Department NEUROFARBA − Pharmaceutical and nutraceutical section, University of Firenze, via Ugo Schiff 6, 50019 Sesto Fiorentino, Florence Italy; ‡ Department NEUROFARBA − Pharmaceutical and nutraceutical section; Laboratory of Molecular Modeling Cheminformatics & QSAR, University of Firenze, via Ugo Schiff 6, 50019 Sesto Fiorentino, Florence, Italy; § Department of Biochemistry and Molecular Biology, College of Medicine, University of Florida, Box 100245, Gainesville, Florida 32610, United States; ∥ Department NEUROFARBA − Pharmaceutical and nutraceutical section, University of Firenze, viale Gaetano Pieraccini 6, 50139 Firenze, Florence, Italy

## Abstract

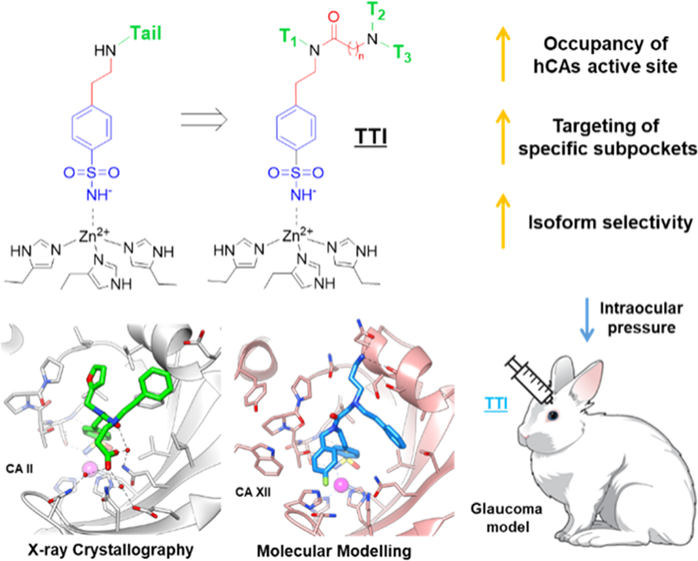

The “tail approach” has become a milestone in human carbonic anhydrase inhibitor (hCAI) design for various therapeutics, including antiglaucoma agents. Besides the classical hydrophobic/hydrophilic division of hCAs active site, several subpockets have been identified at the middle/outer active sites rim, which could be targeted to increase the CAI isoform selectivity. This postulate is explored here by three-tailed benzenesulfonamide CAIs (**TTI**) to fully exploit such amino acid differences among hCAs. In this proof-of-concept study, an extensive structure–activity relationship (SAR) study was carried out with 32 such benzenesulfonamides differing in tails combination that were assayed for hCAs I, II, IV, and XII inhibition. A structural study was undertaken by X-ray crystallography and *in silico* tools to assess the ligand/target interaction mode. The most active and selective inhibitors against isoforms implicated in glaucoma were assessed in a rabbit model of the disease achieving an intraocular pressure-lowering action comparable to the clinically used dorzolamide.

## Introduction

Carbonic anhydrases (CAs, EC 4.2.1.1) are among the most efficient catalysts, speeding up the simple yet physiologically essential reaction in all kingdoms: the reversible hydration of carbon dioxide to bicarbonate and protons.^1^ Among the eight genetically unrelated CA families α, β, γ, δ, η, ζ, θ, and ι,^2−9^ α-CAs are uniquely present in higher vertebrates.^2,10^ In particular, humans express 15 α-CA isoforms (hCAs) which differ in catalytic activity, subcellular/tissue localization, and physiological role.^11^ Therefore, hCAs are involved in multiple physiological processes and their levels of activities are linked to many human disorders such as glaucoma, retinal/cerebral edema, retinitis pigmentosa, other retinopathies, stroke, epilepsy, sterility, osteoporosis, altitude sickness, cariogenesis, neurodegeneration, obesity, and cancer.^12−14^ As a result, almost all catalytically active hCAs have generated great interest for the design of inhibitors (carbonic anhydrase inhibitors, CAIs) or activators (CAAs) with biomedical applications.^15^ Although initially CAIs were used as diuretics, antiglaucoma agents, antiepileptics, and for the management of altitude sickness,^2^ a new generation of CAIs are being developed for the treatment of cancers, obesity, inflammation, neuropathic pain, infections, and neurodegenerative disorders.^16−21^ CAAs are also of interest in the field of cognition, aging, and neurodegeneration.^22^

**Figure 1:**
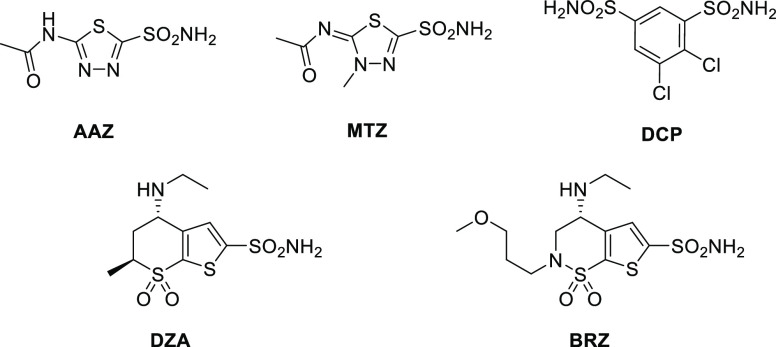
Clinically used antiglaucoma CAIs.

Nevertheless, the use as antiglaucoma agents is still the main therapeutic application of CAIs. In fixed-drugs combinations (mainly with prostaglandin analogues and β-blockers), CAIs continue to be marketed worldwide and widely used.^23^ Acetazolamide (**AAZ**), methazolamide (**MTZ**), and dichlorophenamide (**DCP**) are first-generation CAIs used as systemic drugs for the management of this disease (Figure 1). Dorzolamide (**DRZ**) and brinzolamide (**BRZ**) represent second-generation inhibitors used topically, as eye drops, with less side effects compared to first-generation drugs.^24^ However, none of these drugs possess a selective inhibition profile against the hCA isoforms mainly implicated in the disease that are hCA II (main isoform), IV, and XII. Considering that the current therapies are overall often inadequate given that multiple classes of medications have to be coadministered to control intraocular pressure (IOP) efficiently,^25^ it might be of crucial importance to optimize the single CAI agents, by increasing their efficacy (against the target CAs) and decreasing adverse events (improving their selectivity of action).

The 12 catalytically active hCAs (isoforms VIII, X, and XI are catalytically inactive) are characterized by a Zn(II) ion, which is tetrahedrally coordinated by three histidine residues and a solvent molecule that are situated at the base of a 13 Å deep conical cavity portioned into hydrophobic and hydrophilic sides.^11,15,26^ As the hCAs catalytic domains are structurally homologous and conserved in amino acid sequence identity, it is rather challenging to achieve targeted inhibition of a specific hCA isozyme over others. Despite this, many new approaches have been developed for this purpose, especially over the last two decades.^15^

So far, four unique CA inhibition mechanisms have been validated by both kinetic and structural assessments:^15,27^ (1) zinc binding, which consists of the direct coordination of a catalytical Zn(II) ion with a tetrahedral or trigonal bipyramidal coordination geometry (sulfonamides, sulfamides, sulfonates, anions, mono-dithiocarbamates, xanthates, thioxanthates, carboxylates, hydroxamates, benzoxaboroles, selenols); (2) anchorage to the zinc-bound water molecule/hydroxide ion (phenols, thiophenols, polyphenols, carboxylates, polyamines, 2-thioxocoumarins, sulfocoumarins); (3) occlusion of the active site entrance (coumarins and bioisosters); and (4) binding out of the active site (a unique carboxylic acid derivative exhibited this inhibition mode to date).

Undoubtedly, zinc binders, such as sulfonamides and their bioisosters sulfamates and sulfamides in a prominent position, are among the most effective and investigated derivatives in the field of CA inhibition as well as in the related clinical context.^11,15^

**Figure 2:**
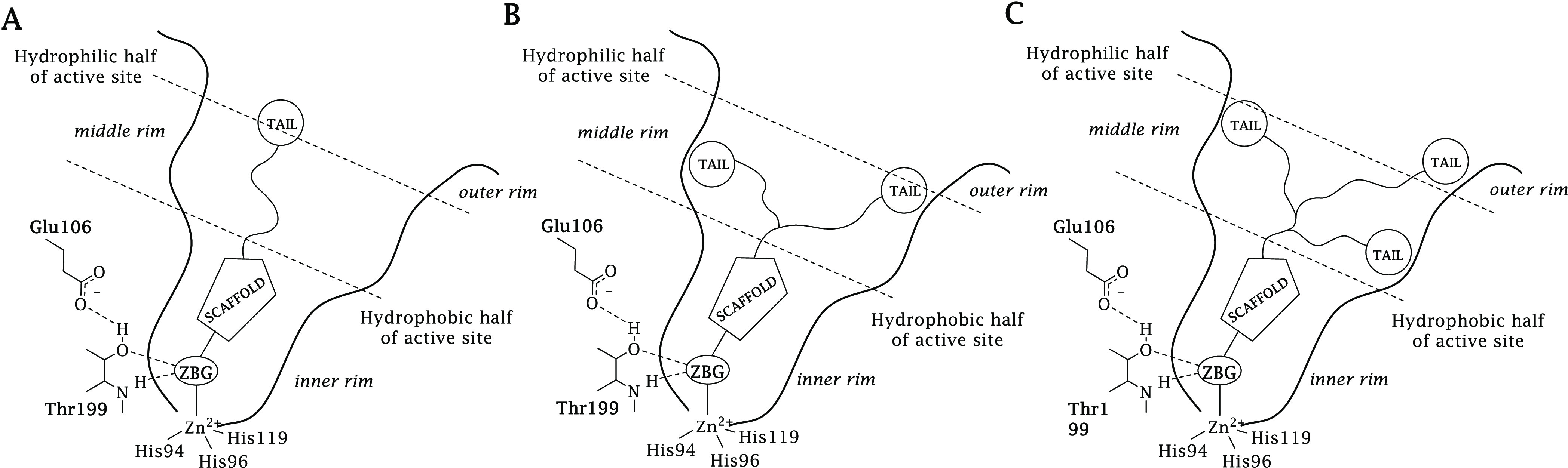
Schematic representation of the (A) “tail”, (B) “two-tails”, and (C) “three-tails” approach for the design of zinc-binding CAIs.

In fact, most efforts have been made on this class of CAIs to achieve isozyme selectivity of action, to lower the side effects consequent to promiscuous inhibition.^28^ As simple as effective, the so-called “tail approach” made its appearance in the field of CA inhibition in 1999 and led to the development of a large number of studies and compounds that expanded the database of CA isoform-selective inhibitors by appending a wide spectrum of chemical functionalities, named tails, to the main zinc-binding scaffold.^29−35^ The original aim was to increase the water solubility^29^ and subsequently membrane (im)permeability of aromatic sulfonamide derivatives.^32^ Afterward, the design was shifted toward the modulation of the interactions between the ligand and the middle and outer rims of the hCAs active sites, which contain the most variable polypeptide regions among the various isoforms, to increase isoform specificity. Simple tailed CAIs are composed of the following elements: (i) a zinc-binding function, (ii) a main scaffold that can include a linker, and (iii) the tail (Figure 2A).

An extension of this approach was proposed in 2015 by Tanpure et al.,^36^ with the simultaneous inclusion of two tails of diverse nature onto aromatic sulfonamide scaffolds, at a nitrogen atom branching point, allowing distinct binding to the hydrophobic and hydrophilic sections of the hCAs active site (Figure 2B). However, a limited number of compounds were reported (three), and an *in vitro* assay was performed solely on hCA II, which makes this pioneering study rather unfulfilled. More recently, Fares et al. have used a similar approach proposing a diverse type of dual tails to benzenesulfonamide CAIs.^37^

The detailed knowledge of the active site composition and architecture of hCAs (mostly available by X-ray crystallographic studies, except for CAs VA and VB) derived from many previous studies^38−40^ led to the conclusion that the simple hydrophobic/hydrophilic division of the isoforms binding pocket may no longer be sufficient. In fact, some CA isozymes do not exhibit such a precise distinction as originally noted in hCA I, II, and IX,^13^ and a bulk of accessory subpockets exist, which differentiate the various CA isoforms. Here, the inclusion of a third tail is proposed as an approach to improve the matching and fitting of the target–ligand interaction within the different hCAs active sites (Figure 2C).

As a first proof of concept of this improved approach, a diverse array of tail combinations were investigated with the aim of identifying suitable isoform imprints. Described here is the screening of hCA isozymes I, II, IV, and XII with 32 benzenesulfonamide derivatives incorporating three tails. In the context of the antiglaucoma CAI application, hCA I is the main off-target isoform as it is widespread in red blood cells and many other tissues.^2^ A comprehensive structural study was also undertaken by X-ray crystallography with hCA II and *in silico* with isozymes hCA I, IV, and XII, to assess the ligand–target interaction modes. A selection of the three-tailed inhibitors most active against hCAs implicated in glaucoma was assessed *in vivo* in a rabbit model of the diseases and compared to classical clinically used CAIs.

## Results and Discussion

### Drug Design and Chemistry

Currently, the tail approach has been a focus of CAIs research area with most design studies adopting the *p-*substituted benzenesulfonamide scaffold as a main foothold to include a variety of chemical frameworks.^15^ In fact, avoiding heteroaromatic sulfonamide scaffolds markedly eases the synthesis procedures, moving the focus on the inclusion of pendants on the inhibitor structure.^36^ Likewise, to converge efforts and attention on studying the three-tailing effects on CA inhibition, a *p-*substituted benzenesulfonamide was here adopted as a CAI scaffold.

**Figure 3:**
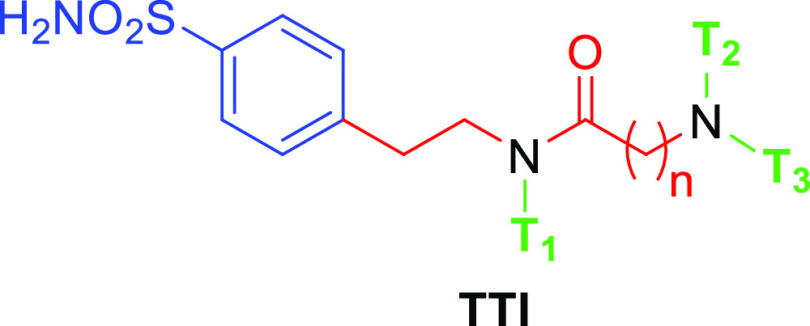
General structure of the designed three-tailed inhibitors (**TTIs**).

It should be stressed that it is not possible to easily include three chemically diverse tails on a single branching atom (e.g., a nitrogen atom, as proposed by Tanpure et al. in the two-tails approach),^36^ unless obtaining an ammonium salt or a chiral center. As a result, among several identified alternatives to branch a spacer attached to the main scaffold into three tails, the general structure **TTI** (Figure 3) was selected to combine easy and versatile chemistry with the possibility to extend it to many diverse chemical groups, which is relevant for producing a range of tail combinations. As a result, **TTI** was designed in the following manner: (i) a benzenesulfonamide scaffold (blue), which assures the interaction with the zinc ion and the bottom of the active site; (ii) an ethylenic spacer (red), which has the function to allow sufficient space between the main scaffold and the tails; (iii) a first ramification point (N atom, in black) from which the first tail T_1_ (green) branches off; (iv) an amide-based spacer (red); and (v) a second intersection point (N atom, in black) by which T_2_ and T_3_ (green) branch off. Having the benzenesulfonamide bound to the Zn(II) at the bottom of the active site, the linkers in red (Figure 3) were chosen in such a way as to explore a vast chemical space at the middle and outer rims of the binding clefts.

**Scheme 1:**
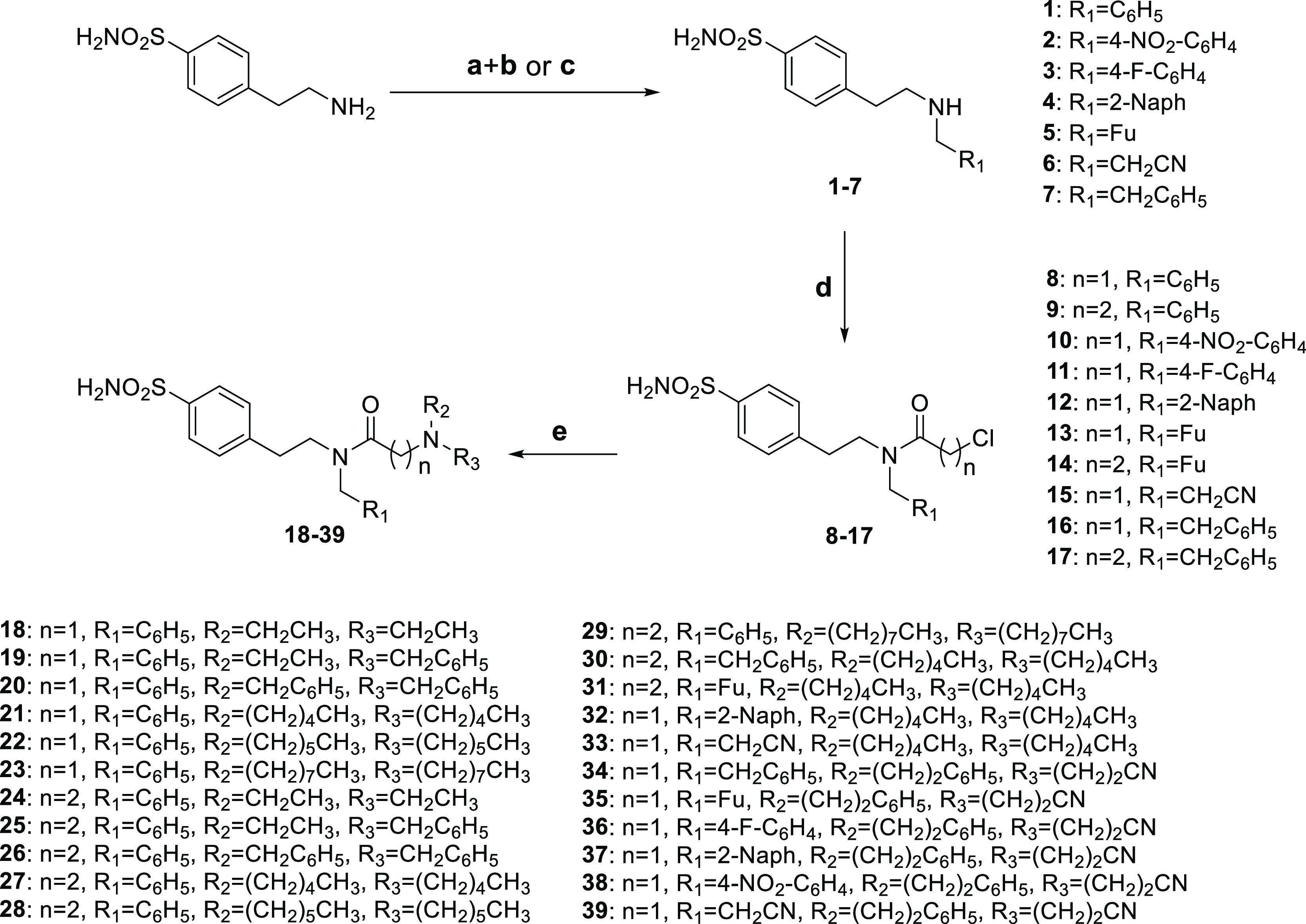
Reagents and Conditions: (a) R_1_CHO, Anhydrous MeOH, Reflux, 4 h; (b) NaBH_4_, Anhydrous MeOH, Reflux, 0.5–2 h; (c) R_1_CH_2_X, TEA, Anhydrous DMF; (d) ClCO(CH_2_)*_n_*Cl, K_2_CO_3_, Acetone, Room Temperature (r.t.), 1 h; (e) R_2_R_3_NH, TEA, Anhydrous ACN, Reflux, 4–24 h

**Scheme 2:**
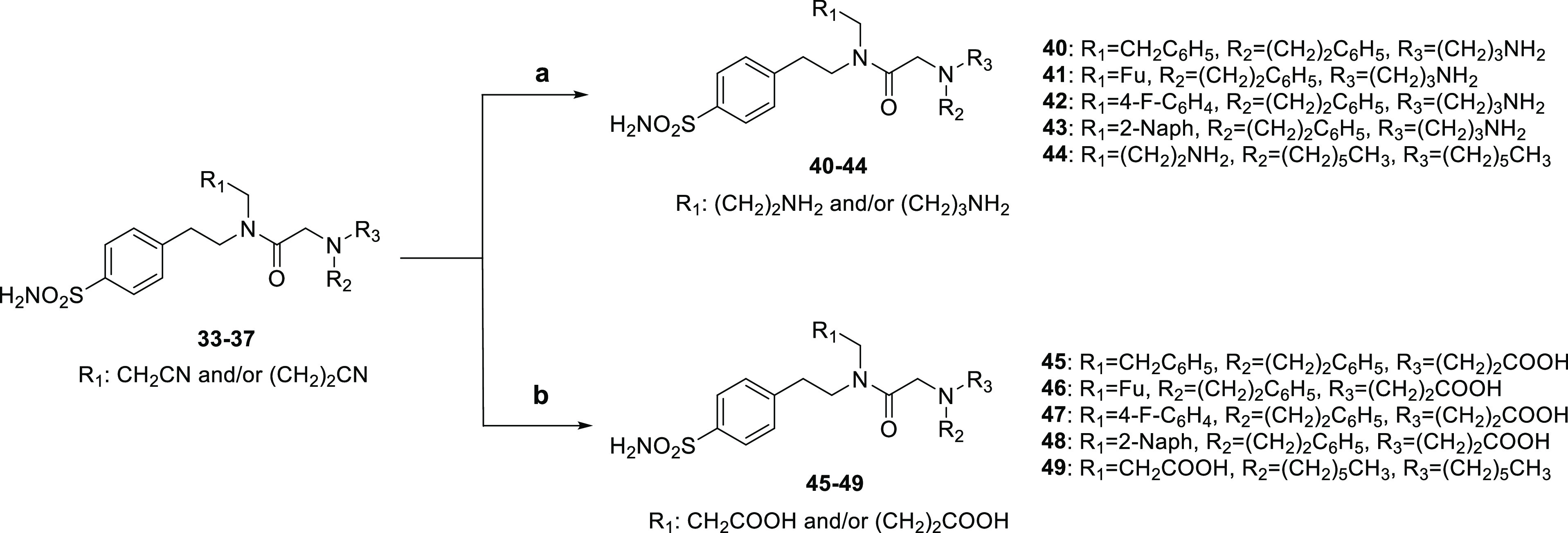
Reagents and Conditions: (a) H_2_, Ni/Raney, NaOH, EtOH, r.t., Overnight (o.n.); (b) NaOH, EtOH, Reflux, o.n

**Scheme 3:**
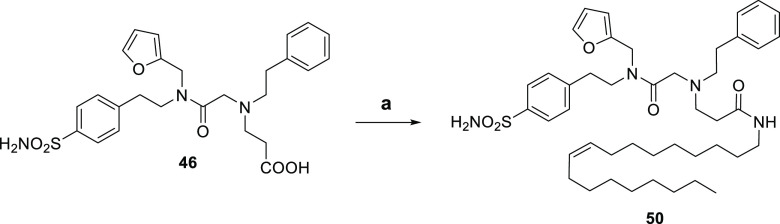
Reagents and Conditions: (a) Oleylamine, EDC·HCl, DMAP, Anhydrous DMF, r.t., o.n

The synthesis strategies adopted to yield the **TTI** derivatives are reported in Schemes 1–3. T_1_ was introduced on the 4-(2-aminoethyl)benzenesulfonamide by reductive amination with the proper aromatic aldehyde and sodium borohydride in MeOH or, alternatively, by nucleophilic substitution with the appropriate halides in anhydrous *N*,*N*-dimethylformamide (DMF) and in the presence of tetraethylammonium (TEA) to furnish secondary amines **1–5** and **6** and **7**, respectively. The latter were reacted with chloroacetyl chloride or chloropropionyl chloride in acetone and in the presence of K_2_CO_3_ to provide amides **8–17**. T_2_ and T_3_ were finally included through a nucleophilic substitution with commercially available or synthesized secondary amines in anhydrous ACN and TEA as a base to produce **TTIs 18–33**. The nitrile derivatives **33–37** were further converted to the corresponding amines **40–44** through a Ni/Raney-catalyzed hydrogenation or hydrolyzed in NaOH_(aq)_ into the corresponding carboxylic acids **45–49** (Scheme 2). Additionally, the markedly hydrophobic oleylamide derivative **50** was yielded by coupling the carboxylic acid **46** with oleylamine in the presence of EDC and 4-dimethylaminopyridine (DMAP) in anhydrous DMF.

All derivatives were purified by silica gel chromatography eluting with MeOH/DCM gradients and fully characterized by ^1^H NMR, ^13^C NMR, and high-resolution mass spectrometry (HRMS) (Supporting Information).

### Carbonic Anhydrase Inhibition

In this first screening, mono-tailed (**1–7**) and three-tailed (**18–50**) compounds were analyzed by a stopped-flow kinetic assay with hCA isoforms I, II, IV, and XII.^41^ HCAs II, IV, and XII are involved in glaucoma with the last isoform being reported to be upregulated in the eyes of glaucoma patients. Thus, all of them are involved in this disease, both in the elevation of intraocular pressure (IOP) and the decrease of blood flow and oxygen supply within the hypoxic neovascular retinic tissues.^42^ HCA IV was reported to be involved in stroke, glaucoma, retinitis pigmentosa, astrocytomas, and gliomas.^12^ HCA XII is also validated as an anticancer target (being overexpressed on the membrane of hypoxic tumor cells),^17^ and recently, overexpression of this isoform has also been linked to inflammation.^19^ HCA I is a main off-target isoform for the therapeutic application of CAIs in ocular diseases, as this isoform is widespread in red blood cells and many other tissues.^2^

Generally, the inhibition data reported in Table 1 highlighted that mono-tailed compounds **1–7** were medium to high nanomolar inhibitors of hCA I (*K*_I_ = 68.4–458.1 nM), II (*K*_I_ = 62.8–153.7 nM), and XII (*K*_I_ = 55.4–113.2 nM), and weak inhibitors of hCA IV with inhibition constant (*K*_I_) values in the low micromolar range (1.1–6.2 μM).

**Table 1:** Inhibition Data of Human CA Isoforms CA I, II, IV, and XII with Sulfonamides **1–7**, **18–50** Reported Here and the Standard Sulfonamide Inhibitor Acetazolamide (**AAZ**) by a Stopped-Flow CO_2_ Hydrase Assay^41^

					*K*_I_^a^ (nM)			
cmpd	*n*	R_1_	R_2_	R_3_	CA I	CA II	CA IV	CA XII
**1**		C_6_H_5_			95.3	98.4	2854.4	65.4
**2**		4-NO_2_-C_6_H_4_			224.3	120.9	1685.3	77.4
**3**		4-F-C_6_H_4_			112.8	78.5	1196.7	60.1
**4**		2-Naph			458.1	87.1	6248.1	78.6
**5**		Fu			68.4	62.8	1584.5	55.4
**6**		CH_2_CN			105.3	153.7	5547.2	113.2
**7**		CH_2_C_6_H_5_			278.4	89.1	3587.4	104.3
**18**	1	C_6_H_5_	CH_2_CH_3_	CH_2_CH_3_	786.6	8.3	4147.5	43.9
**19**	1	C_6_H_5_	CH_2_CH_3_	CH_2_C_6_H_5_	4210.4	391.6	>10000	82.6
**20**	1	C_6_H_5_	CH_2_C_6_H_5_	CH_2_C_6_H_5_	865.9	412.3	>10000	98.8
**21**	1	C_6_H_5_	(CH_2_)_4_CH_3_	(CH_2_)_4_CH_3_	506.1	124.5	>10000	69.4
**22**	1	C_6_H_5_	(CH_2_)_5_CH_3_	(CH_2_)_5_CH_3_	878.7	237	>10000	92.8
**23**	1	C_6_H_5_	(CH_2_)_7_CH_3_	(CH_2_)_7_CH_3_	946.7	843.8	>10000	99.4
**24**	2	C_6_H_5_	CH_2_CH_3_	CH_2_CH_3_	184.7	8.9	3928.8	61.1
**25**	2	C_6_H_5_	CH_2_CH_3_	CH_2_C_6_H_5_	544.3	79.6	>10000	90.4
**26**	2	C_6_H_5_	CH_2_C_6_H_5_	CH_2_C_6_H_5_	692.3	559.2	4640.8	302.5
**27**	2	C_6_H_5_	(CH_2_)_4_CH_3_	(CH_2_)_4_CH_3_	563.6	522.6	3244.8	100.3
**28**	2	C_6_H_5_	(CH_2_)_5_CH_3_	(CH_2_)_5_CH_3_	308.2	578.4	3455.4	77.8
**29**	2	C_6_H_5_	(CH_2_)_7_CH_3_	(CH_2_)_7_CH_3_	209.3	778.8	>10000	280
**30**	2	CH_2_C_6_H_5_	(CH_2_)_5_CH_3_	(CH_2_)_5_CH_3_	518.4	780.8	3413.2	62.5
**31**	2	Fu	(CH_2_)_5_CH_3_	(CH_2_)_5_CH_3_	220.1	60.4	3153.7	9.7
**32**	1	2-Naph	(CH_2_)_5_CH_3_	(CH_2_)_5_CH_3_	541.4	4562.9	>10000	61.7
**33**	1	CH_2_CN	(CH_2_)_5_CH_3_	(CH_2_)_5_CH_3_	395.9	52.5	3478.3	8.6
**34**	1	CH_2_C_6_H_5_	(CH_2_)_2_C_6_H_5_	(CH_2_)_2_CN	777.3	368.5	>10000	75.5
**35**	1	Fu	(CH_2_)_2_C_6_H_5_	(CH_2_)_2_CN	300.8	73.2	457.4	8.7
**36**	1	4-F-C_6_H_4_	(CH_2_)_2_C_6_H_5_	(CH_2_)_2_CN	676.4	133	4133.8	9.8
**37**	1	2-Naph	(CH_2_)_2_C_6_H_5_	(CH_2_)_2_CN	685	247.5	3812.9	64.9
**38**	1	4-NO_2_-C_6_H_4_	(CH_2_)_2_C_6_H_5_	(CH_2_)_2_CN	407.5	264.2	2421.5	89.5
**39**	1	CH_2_CN	(CH_2_)_2_C_6_H_5_	(CH_2_)_2_CN	61.6	0.7	726.6	8.9
**40**	1	CH_2_C_6_H_5_	(CH_2_)_2_C_6_H_5_	(CH_2_)_3_NH_2_	242.4	367.3	2149.2	83.7
**41**	1	Fu	(CH_2_)_2_C_6_H_5_	(CH_2_)_3_NH_2_	246.7	57	374.1	42.7
**42**	1	4-F-C_6_H_4_	(CH_2_)_2_C_6_H_5_	(CH_2_)_3_NH_2_	451.4	30.4	365.3	0.6
**43**	1	2-Naph	(CH_2_)_2_C_6_H_5_	(CH_2_)_3_NH_2_	506.7	5.6	819.2	10.5
**44**	1	(CH_2_)_2_NH_2_	(CH_2_)_5_CH_3_	(CH_2_)_5_CH_3_	435.8	2924.8	913.9	32.5
**45**	1	CH_2_C_6_H_5_	(CH_2_)_2_C_6_H_5_	(CH_2_)_2_COOH	203.5	72	2330.5	29.7
**46**	1	Fu	(CH_2_)_2_C_6_H_5_	(CH_2_)_2_COOH	79.5	2.4	335.5	7.1
**47**	1	4-F-C_6_H_4_	(CH_2_)_2_C_6_H_5_	(CH_2_)_2_COOH	95.8	23.5	419.3	8.8
**48**	1	2-Naph	(CH_2_)_2_C_6_H_5_	(CH_2_)_2_COOH	197	72.5	680.6	6.8
**49**	1	CH_2_COOH	(CH_2_)_5_CH_3_	(CH_2_)_5_CH_3_	285.5	585.7	45.8	9.9
**50**	1	Fu	(CH_2_)_2_C_6_H_5_	(CH_2_)_2_CONHoleyl	737.9	132	1807.1	5.5
AAZ					250	12	74	5.7

a Mean from three different assays, by a stopped-flow technique (errors were in the range of ±5–10% of the reported values). Fu = furyl; Naph = naphthyl.

In detail, compounds **1** (R_1_ = C_6_H_5_) and **5** (R_1_ = Fu) inhibited the off-target hCA I in the medium nanomolar range (*K*_I_ = 95.3 and 68.4 nM, respectively), while compounds **2**, **4**, and **7** acted as weaker inhibitors (*K*_I_ = 224.3–458.1 nM). In fact, the introduction of bulky substituents (**2** and **4**, *K*_I_s of 224.3 and 458.1 nM) or the elongation of the chain (**7**, *K*_I_ of 278.4 nM) in R_1_ decreased the action against hCA I compared to compound **1**.

The aryl-tailed compounds **1**–**6** acted as medium nanomolar inhibitors (*K*_I_ = 62.8–120.9 nM) against hCA II, with compound **5** (R_1_ = Fu) being the single-tail isoform inhibitor. Compound **7** (R_1_ = CH_2_CN) reported instead the worst inhibition of action against hCA II (*K*_I_ = 153.7 nM).

HCA IV was the least inhibited by compounds **1**–**7**. In this context, derivatives **2** (*K*_I_ = 1.6 μM), **3** (*K*_I_ = 1.1 μM), and **5** (*K*_I_ = 1.5 μM) resulted to be significantly better inhibitors than the bulkier derivative **4** (R_1_ = 2-Naph, *K*_I_ value of 6.2 μM).

HCA XII was inhibited almost similarly by the single-tail compounds **1**–**7**. Nonetheless, again derivative **5** (R_1_ = Fu) stood out as the best inhibitor (*K*_I_ = 55.4 nM), whereas the cyanoalkyl- and phenethyl-tailed compounds **6** and **7** exhibit *K*_I_s above 100 nM.

Data in Table 1 showed that the development of **1–7** upon inclusion of two other tails to synthesize compounds **18–50** significantly affected the inhibition profiles against the panel of CA isoforms. In fact, **TTI**s showed lightly decreased or markedly improved inhibition of hCA XII (*K*_I_s = 0.6–302.5 nM). HCA IV remained the less inhibited isozyme, though inhibition improvement of 1 or 2 orders of magnitude were testified for some compounds (*K*_I_s = 45.8–>10 000 nM). On the whole, no significant improvement of hCA I inhibition was detected with **TTIs** (*K*_I_s = 79.5–4210.4 nM). HCA II showed that the inhibition profiles most affected, both positively and negatively, upon inclusion of additional tails on the scaffold of **1–7** (*K*_I_s = 0.7–4562.9 nM).

To better discuss **TTI**s’ structure–activity relationship (SAR) from Table 1, compounds and related data were distinguished in five subsets: (i) **18–29** (with R_1_ = C_6_H_5_); (ii) **30–33, 44, 49** (with R_2_ = R_3_ = (CH_2_)_5_CH_3_); (iii) **34–39** (R_2_ = (CH_2_)_2_C_6_H_5_ and R_3_ = (CH_2_)_2_CN); (iv) **40–43** (R_2_ = (CH_2_)_2_C_6_H_5_ and R_3_ = (CH_2_)_3_NH_2_); and (v) **45–48** (R_2_ = (CH_2_)_2_C_6_H_5_ and R_3_ = (CH_2_)_2_COOH).

(i) In the first subset, compounds **18** and **20–29** were high nanomolar inhibitors of the ubiquitous off-target hCA I with *K*_I_ values between 184.7 and 946.7 nM, while derivative **24** (R_2_ = R_3_ = CH_2_CH_3_) showed the best inhibitory profile (*K*_I_ = 184.7 nM). Instead, compound **19** (R_2_ = CH_2_CH_3_ and R_3_ = CH_2_C_6_H_5_) resulted in the worst hCA I inhibitor among all synthesized compounds (*K*_I_ = 4210.4 nM).

The glaucoma-implicated isoform hCA II was inhibited in the nanomolar range (*K*_I_ = 8.3–843.8 nM) and, in particular, the introduction of R_2_ = R_3_ = CH_2_CH_3_ for compounds **18** (*n* = 1) and **24** (*n* = 2) and R_2_ = CH_2_CH_3_ and R_3_ = CH_2_C_6_H_5_ for derivative **25** (*n* = 2) increased the inhibition profile against this isoform (*K*_I_ = 8.3, 8.9, and 79.6 nM, respectively). Thus, derivative **18** is the most hCA II selective compound (CA I/CA II = 94).

Only compounds **18**, **24**, and **26–28** inhibited hCA IV with *K*_I_ values in the range of 3.2–4.6 μM, while the other compounds of this series showed no activity below 10 μM.

All derivatives potently inhibited the other glaucoma-associated isoform, hCA XII, with *K*_I_ values below 100 nM, except for compounds **26** and **29** that were also the worst inhibitors among all of the synthesized compounds against this isoform (*K*_I_ = 280.0 and 302.5 nM). Compound **18** showed the best inhibitory profile of this series (*K*_I_ = 43.9 nM).

The importance of the linker length (*n* = 1, 2) is pointed out from the activity analysis of this first subset. In fact, the elongation of the chain between R_1_ and R_2_/R_3_ increased the activity against hCA I, II and IV, which possess the smallest binding cavities, as a longer linker (*n* = 2) can shift the tails R_2_/R_3_ toward the rim of the active site, removing the ligand–target steric encumbrance. On the other hand, the larger active sites of hCA XII are able to host bulky substituents and the introduction of the linker *n* = 2, which drives the tails R_2_/R_3_ out from the active site, may decrease the activity by weakening the ligand–target interactions.

(ii) Comparing the second subset (**30–33**, **44**, **49** with R_2_ = R_3_ = (CH_2_)_5_CH_3_ compounds) with the first subset R_2_/R_3_-analogues **22** and **28**, it was highlighted that the introduction of Fu and CH_2_CN in R_1_ increased the activity against the off-target hCA I and hCA II, such as observed in compounds **31** (hCA I *K*_I_ = 220.1 nM; hCA II *K*_I_ = 60.4 nM) and **33** (hCA I *K*_I_ = 395.9 nM; hCA II *K*_I_ = 52.5 nM). On the other hand, for R_1_ = CH_2_C_6_H_5_ (**30**) and 2-Naph (**32**), the activity on hCA II strongly decreased for both substituents (*K*_I_ = 780.8 nM and 4.5 μM, respectively), while a weak increase in inhibition was observed for compound **30** (*K*_I_ = 518.4 nM) and a decrement for **32** (*K*_I_ = 541.4 nM) against hCA I.

HCA IV was weakly inhibited by **30**–**32** with *K*_I_ values in the micromolar range of 3.1–3.4 μM. Furthermore, the tail R_1_ = CH_2_CN reduction of compounds **33** into amine **44** decreased the activity on hCA II by 55 times (*K*_I_ = 2.9 μM) and increased the activity on hCA IV by 3 times (*K*_I_ = 913.9 nM). Instead, the swap of **33** nitrile into carboxylic acid **49** worsened the activity against hCA II by 11 times (*K*_I_ = 585.7 nM), but increased the inhibition profile against hCA IV by 76 times (*K*_I_ = 45.8 nM), obtaining the most potent and selective compounds against this isozyme (CA I/CA IV = 6.2).

In the case of hCA XII, all compounds showed a good activity against the target and, in particular, compounds **31** (*K*_I_ = 9.7 nM), **33** (*K*_I_ = 8.6 nM), and **49** (*K*_I_ = 9.9 nM) inhibited this isoform with *K*_I_ in the low nanomolar range while **30**, **32**, and **44** acted as medium nanomolar inhibitors (*K*_I_ = 32.5–62.5 nM).

Generally, for this subset, it was observed that the concomitant presence of R_2_ = R_3_ = (CH_2_)_5_CH_3_ with a 2-Naph in R_1_ (**32**) worsened the activity by 19 times against hCA II (*K*_I_ = 4.5 μM) and increased the activity by 1.5 times against hCA XII (*K*_I_ = 61.7 nM) with respect to the analogue **22** (R_1_ = C_6_H_5_), improving the CA II/CA XII selectivity from 2.5 to 74 times. Of note, the presence of a potentially charged moiety in R_1_ such as (CH_2_)_2_NH_2_ (**44**) or better CH_2_COOH (**49**) increased the activity against hCA IV, which possesses a wider hydrophilic half in the active site with respect to the other hCAs with many acidic/basic residues at the middle rim of the cavity.

(iii) The third subset (**34–39**) is characterized by the introduction of a hydrophobic tail R_2_ = (CH_2_)_2_C_6_H_5_, a polar one R_3_ = (CH_2_)_2_CN, and a variable pendant R_1_. Only compound **39** R_1_ = (CH_2_CN) was a medium nanomolar inhibitor (*K*_I_ = 61.6 nM), which resulted to be the most potent agent against the off-target hCA I, whereas **34**–**38** acted in the high nanomolar range (*K*_I_ = 300.8–777.3 nM).

The glaucoma-associated hCA II was potently inhibited by derivative **39** with *K*_I_ in the subnanomolar range (0.7 nM), resulting the most potent and third selective inhibitor against this isozyme (CA I/CA II = 88.0), while **35** (R_1_ = Fu) acted in the medium nanomolar range with *K*_I_ = 73.2 nM and derivatives **34** and **36**–**38** showed *K*_I_ values between 133.0 and 368.5 nM.

The best inhibitors against hCA IV within this subset were **35** and **39** with *K*_I_ in the high nanomolar range (457.4 and 726.6 nM, respectively), whereas **36**–**38** were low micromolar inhibitors with *K*_I_ values between 2.4 and 4.1 μM and derivative **34** (R_1_ = CH_2_C_6_H_5_) acted with *K*_I_ > 10 μM.

The target hCA XII was strongly inhibited by all compounds of the subset with compounds **35**, **36**, and **39** acting in a low nanomolar range (*K*_I_ = 8.7, 9.8, and 8.9 nM, respectively), while **34**, **37**, and **38** were medium nanomolar inhibitors (*K*_I_ = 75.5, 64.9, and 89.5 nM, respectively). In this case, derivative **36** resulted in the third most selective inhibitor against hCA XII (CA I/CA XII = 69.8).

The comparison of compounds **37** and **39** from subset (iii) with the second subset analogues **32** and **33** (R_2_ = R_3_ = (CH_2_)_5_CH_3_) pointed out that the substitution of R_2_ and R_3_ with the tails (CH_2_)_2_C_6_H_5_ and (CH_2_)_2_CN, respectively, generally increased the activity against hCA II and IV, with the opposite effect against hCA I and no significant effect against hCA XII.

(iv) The fourth series (**40**–**43**) was obtained by reducing R_3_ = (CH_2_)_2_CN to obtain primary amine tails in the aforesaid derivatives **34**–**37**, introducing a potentially positively charged pendant. This structural modification led to a general increment of the activity against hCA I, II, IV, and XII, suggesting that a strong polar interaction is favorable for the binding and might take place in all five active sites.

In detail, the four compounds resulted to be high nanomolar inhibitors of hCA I with *K*_I_ in the 242.4–506.7 nM range. Moreover, it is observed that **40** (R_1_ = CH_2_C_6_H_5_) and **41** (R_1_ = Fu) inhibited this isoform with a 2-fold potency (*K*_I_ = 242.4 and 246.7 nM, respectively) with respect to **42** (R_1_ = 4-F-C_6_H_5_) and **43** (R_1_ = 2-Naph), which showed a *K*_I_ of 451.4 and 506.7 nM, respectively.

Derivatives **40**–**43** were good inhibitors of the glaucoma-associated hCA II with *K*_I_s in the high nanomolar range for **40** (*K*_I_ = 367.3 nM), medium nanomolar range for **41** and **42** (*K*_I_ = 57.0 and 30.4 nM, respectively), and low nanomolar range for **43** (*K*_I_ = 5.6 nM), which was the second most selective obtained inhibitor against this isoform (CA I/CA II = 90.5).

Interestingly, it was observed that the introduction of a positively charged tail increased the activity against hCA IV at least 4 times for **40** (*K*_I_ = 2.1 μM), 1.2 times for **41** (*K*_I_ = 374.1 nM), 11 times for **42** (*K*_I_ = 365.3 nM), and 4.5 times for **43** (*K*_I_ = 819.2 nM) with respect to their analogues of the third subset (**34**–**37**).

The glaucoma-related hCA XII was strongly inhibited by **42** with a subnanomolar *K*_I_ of 0.6 nM that makes it the most potent and selective compound against this isoform (selectivity ratio CA I/CA XII = 752.3), whereas **40** (*K*_I_ = 83.7 nM), **41** (*K*_I_ = 42.7 nM), and **43** (*K*_I_ = 10.5 nM) acted with a *K*_I_ in the medium nanomolar range.

(v) The fifth subset (**45**–**48**) obtained by the introduction of a potentially negatively charged tail in R_3_ showed a general increment of the inhibition activity against hCA I, II, IV, and XII compared to their analogues **34**–**37**.

In detail, compounds **46** (*K*_I_ = 79.5 nM) and **47** (*K*_I_ = 95.8 nM) acted as medium nanomolar inhibitors against the off-target CA I, whereas the introduction of a more encumbering R_1_ (CH_2_C_6_H_5_ and 2-Naph), such as in **45** and **48**, lightly decreased the activity to the high nanomolar range (*K*_I_ = 203.5 and 197.0 nM, respectively).

The target hCA II was inhibited in the low nanomolar range by compound **46** (*K*_I_ = 2.4 nM), the second most potent inhibitor against this isozyme, and in the medium nanomolar range by **45** (*K*_I_ = 72.0 nM), **47** (*K*_I_ = 23.5 nM), and **48** (*K*_I_ = 72.5 nM).

The inhibition profile against hCA IV was in the high nanomolar range for derivatives **46**–**47** (*K*_I_ = 335.5, 419.3, and 680.6 nM, respectively) and decreased for compound **45** with a *K*_I_ value of 2.3 μM.

Moreover, derivatives **46**–**48** were low nanomolar inhibitors of hCA XII (*K*_I_ = 7.1, 8.8, and 6.8 nM, respectively), whereas **48** and **46** resulted to be the second and third most potent inhibitors of this glaucoma-associated isoform, while compound **45** acted with a *K*_I_ of 29.7 nM.

Comparing the fourth (**40**–**43**) and fifth subsets (**45**–**48**), it was detected that the presence of R_3_ = (CH_2_)_2_COOH in place of amine tails shifted the activity against hCA I.

Finally, the loss of the hydrophilic tail R_3_ in **50** decreased the activity against hCA I (*K*_I_ = 737.9 nM), II (*K*_I_ = 132.0 nM), and IV (*K*_I_ = 1.8 μM) without effects against hCA XII (*K*_I_ = 5.5 nM), obtaining the second most potent and selective compound against this isoform (CA I/CA XII = 134.2).

As pointed out by data in Table 1, single-tail inhibitors **1**–**7** showed rather flat inhibition profiles against all tested hCAs and no marked isoform selectivity was detected. In contrast, the selectivity of action is often enhanced with TTIs **18**–**50** (selectivity index, SI, in Table S1, Supporting Information).

For instance, starting from compound **1** (R_1_ = C_6_H_5_; SI CA I/CA II = 1.0; CA I/CA XII = 1.5; CA II/CA XII = 1.5; CA IV/CA XII = 43.6), the introduction of various lipophilic pendants in R_2_ and R_3_ (as in **18**–**29**) decreased the activity against all isoforms, except for derivatives **18** and **24** where the inhibition profile against hCA II and XII was increased. Interestingly, CA I/CA II selectivity of **18–27** was improved up to an SI of 94.8 for compound **18**. Compounds **28** and **29** (CA I/CA II = 0.5 and 0.3, respectively) were instead the most selective hCA I inhibitors of this subset.

Derivatives **18–28** also exhibited improved selectivity for hCA XII over hCA I (SI 2.3–51.0), whereas **29** showed a greater action against hCA I (SI I/XII = 0.7). Moreover, derivatives **19**–**23** and **26**–**29** showed an increased selectivity for hCA XII over CA II (SI 1.8–9.5), in contrast to **24** and **25** more active against hCA II (SI 0.14 and 0.9). Within this subset, improved selectivity profiles for hCA IV over hCA I and II were not detected. CA IV/XII selectivity increased up to 64.3–>144.1 for the subset **18**–**25**.

In comparison to the single-tail derivative **2**, compound **38** (R_1_ = 4-NO_2_-C_6_H_4_, R_2_ = (CH_2_)_2_C_6_H_5_, R_3_ = (CH_2_)_2_CN) showed an increased selectivity for hCA XII over hCA I, II and IV (SI I/XII 4.6, II/XII 3.0, IV/XII 27.1), while I/II selectivity showed a decrease (SI 1.5).

While derivative **3 (**R_1_ = 4-F-C_6_H_4_) showed SIs equal to I/II 1.4, I/IV 0.1, I/XII 1.9, II/XII 1.3, and IV/XII 19.9, the addition of (CH_2_)_2_C_6_H_5_ and (CH_2_)_2_CN (**36**) in R_2_, and (CH_2_)_3_NH_2_ (**42**) and (CH_2_)_2_COOH (**47**) in R_3_ led to remarkable results in terms of selectivity of action. In detail, selectivity was increased for hCA II over hCA I and for hCA XII over hCA I, II, and IV for compounds **36** (I/II = 5.1, I/XII = 69.8, II/XII = 13.6, IV/XII = 421.8), **47** (I/II = 4.1, I/XII = 10.9, II/XII = 2.7, IV/XII = 47.6), and even more in derivative **42** (I/II = 14.9, I/XII = 753.3, II/XII = 50.7, IV/XII = 608.8). Notably, the introduction of an amine moiety in R_3_ significantly shifted the selectivity toward hCA XII, making compound **42** 752.3 times more active against the glaucoma-associated isoform hCA XII than the off-target hCA I. **42** also showed the best CA IV/CA XII selectivity index with a ratio of 608.8. The nature of R_3_ can also be assumed to be responsible for a >1 SI for hCA IV over I.

Variable outcomes in terms of selectivity of action were observed appending R_2_ and R_3_ tails on the 2-Naph single-tail **4** (SIs CA I/CA II = 5.3, CA I/CA XII = 5.8, CA II/CA XII = 1.1, CA IV/CA XII = 79.5) yielding **32 (**R_2_ = R_3_ (CH_2_)_5_CH_3_, R_3_), **37 (**R_2_ = (CH_2_)_2_C_6_H_5_, R_3_ = (CH_2_)_2_CN), **43 (**R_2_ = (CH_2_)_2_C_6_H_5_, R_3_ = (CH_2_)_3_NH_2_), and **48** (R_2_ = (CH_2_)_2_C_6_H_5_, R_3_ = (CH_2_)_2_COOH). In fact, I/II selectivity decreased for derivative **37** (I/II SI 2.8) and **48** (I/II SI 2.7) up to the inversion displayed by **32** (SI 0.1). In contrast, it strongly increased with amine **43** (I/II SI 90.5). I/XII selectivity was improved for all of these derivatives in the order **32** (I/XII SI = 8.8), **37** (I/XII SI = 10.6), **48** (I/XII SI = 29.0), **43** (I/XII SI = 48.3). The lipophilic **TTI 32** showed great selectivity for hCA XII over hCA II and hCA IV (SI = 74.0 and >162.1, respectively). Carboxylic acid **48** (SI II/XII = 10.7, IV/XII = 100.1) acted likewise. A low II/XII SI increase was observed for nitrile **37** (SI 3.8), while an inversion was detected for amine **43** (SI II/XII = 0.5). The latter also showed an improved I/IV SI value (0.6) with respect to **4** (0.07).

The **TTI** development of derivative **5** (SIs I/II = 1.1, I/IV = 0.04, I/XII = 1.2, II/XII = 1.1, IV/XII = 28.6), to give **31** (R_2_ = R_3_ (CH_2_)_5_CH_3_, R_3_), **35** (R_2_ = (CH_2_)_2_C_6_H_5_, R_3_ = (CH_2_)_2_CN), **41 (**R_2_ = (CH_2_)_2_C_6_H_5_, R_3_ = (CH_2_)_3_NH_2_), **46** (R_2_ = (CH_2_)_2_C_6_H_5_, R_3_ = (CH_2_)_2_COOH), and **50** (R_2_ = (CH_2_)_2_C_6_H_5_, R_3_ = (CH_2_)_2_CONHoleyl), overall increased the selectivity for hCA II over hCA I (3.6, 4.1, 4.3, 33.1, and 5.6, respectively). CA I/IV SIs were overall improved (0.2–0.7) with respect to **5** (except **31**) but not reversed. Interestingly, the reduction of nitrile **35** into amine **41** did not lead to variations in the I/IV selectivity, while the hydrolysis to carboxylic acid **46** decreased it by 3 times. I/IV SI increased instead twice upon formation of amide **50**.

The selective index for hCA XII over hCA I increased for all five derivatives, greatly with **31** (SI 22.7), nitrile **35** (SI 34.6), and amide **50** (SI 134.1), and less with amine **41** (SI 5.8) and carboxylic acid **46** (I/XII = 11.2). These compounds also showed selectivity for hCA XII over hCA II with SIs of 6.2 (**31**), 8.4 (**35**), 1.3 (**41**), and 24.0 (**24**), except for the carboxylic acid **46** (SI 0.3).

Except for derivative **41** (SI 8.8), selectivity for hCA XII over IV ratio was enhanced for compounds **31** (SI 325.1), **35** (SI 52.6), **46** (SI 47.3), and **50** (SI 328.6) with respect to the lead **5**.

The functionalization of **6** (I/II = 0.7, I/IV = 0.02, II/IV = 0.03, I/XII = 0.9, II/XII = 1.4, IV/XII = 49.0) with R_2_ and R_3_ produced derivatives **33** (R_2_ = R_3_ (CH_2_)_5_CH_3_) and **39 (**R_2_ = (CH_2_)_2_C_6_H_5_, R_3_ = (CH_2_)_2_CN) that acted 7.5 and 88.0 times more efficiently against hCA II over hCA I. Moreover, compound **33** showed an increment of SI I/XII (46.0), II/XII (6.1), and IV/XII (404.5). Instead, derivative **39** showed a drastically improved action against hCA II over hCA XII (CA II/CA XII = 0.1) and improved SIs I/XII (6.9) and IV/XII (81.6). The reduction and hydrolysis of the nitrile of derivative **33** to give amine **44** and carboxylic acid **49** led to a selectivity against hCA I over hCA II (CA I/CA II = 0.2 and 0.5, respectively). Interestingly, **49** was the first-in-class selective hCA IV inhibitor over CA I (SI 6.2) and hCA II (SI 12.8) and also showed the lowest IV/XII SI (4.6). Finally, amine and carboxylic acid **44** and **49** showed increased II/XII SI (90.0 and 59.2, respectively).

The R_2_/R_3_ development of compound **7** (R_1_ = CH_2_C_6_H_5_, I/II = 3.1, I/XII = 2.7, II/XII = 0.9, IV/XII = 34.4) to give **30** (R_2_ = R_3_ (CH_2_)_5_CH_3_, R_3_), **34** (R_2_ = (CH_2_)_2_C_6_H_5_, R_3_ = (CH_2_)_2_CN), **40** (R_2_ = (CH_2_)_2_C_6_H_5_, R_3_ = (CH_2_)_3_NH_2_), and **45** (R_2_ = (CH_2_)_2_C_6_H_5_, R_3_ = (CH_2_)_2_COOH) decreased I/II selectivity up to a total inversion with derivatives **30** (SI 0.7) and **40** (SI 0.7). On the contrary, an improvement was detected in the selectivity against hCA XII over hCA I (SI 2.9–10.3), hCA II (SI 2.4–12.5), and hCA IV (SI 54.6–>137.5), except for compound **45** that showed a worsening in the IV/XII selectivity (SI 25.9) compared to the lead **7**.

### X-ray Crystallography

**Figure 4:**
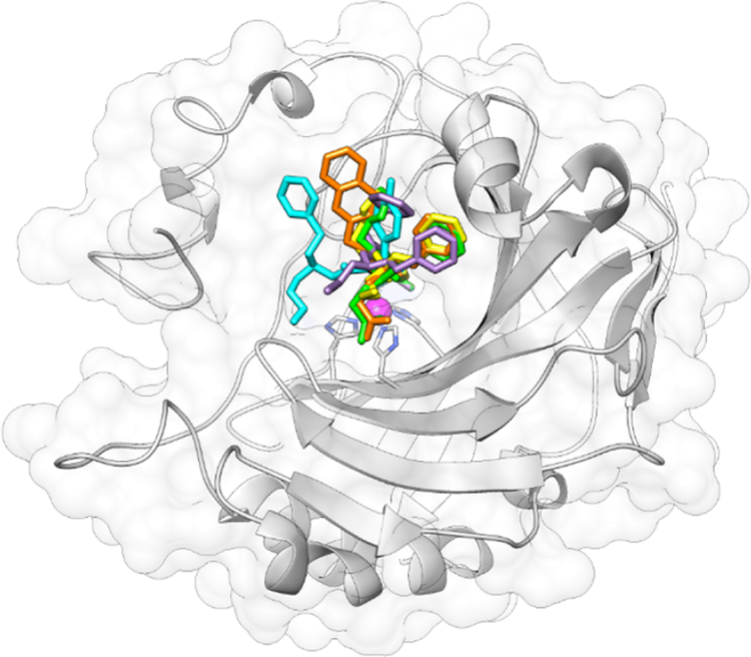
X-ray crystallography: surface representation of hCA II with inhibitors **34** (purple), **41** (yellow), **42** (cyan), **46** (green), and **48** (orange) bound within the active site (PDBs 6WQ4, 6WQ5, 6WQ7, 6WQ8, and 6WQ9, respectively).

**Figure 5:**
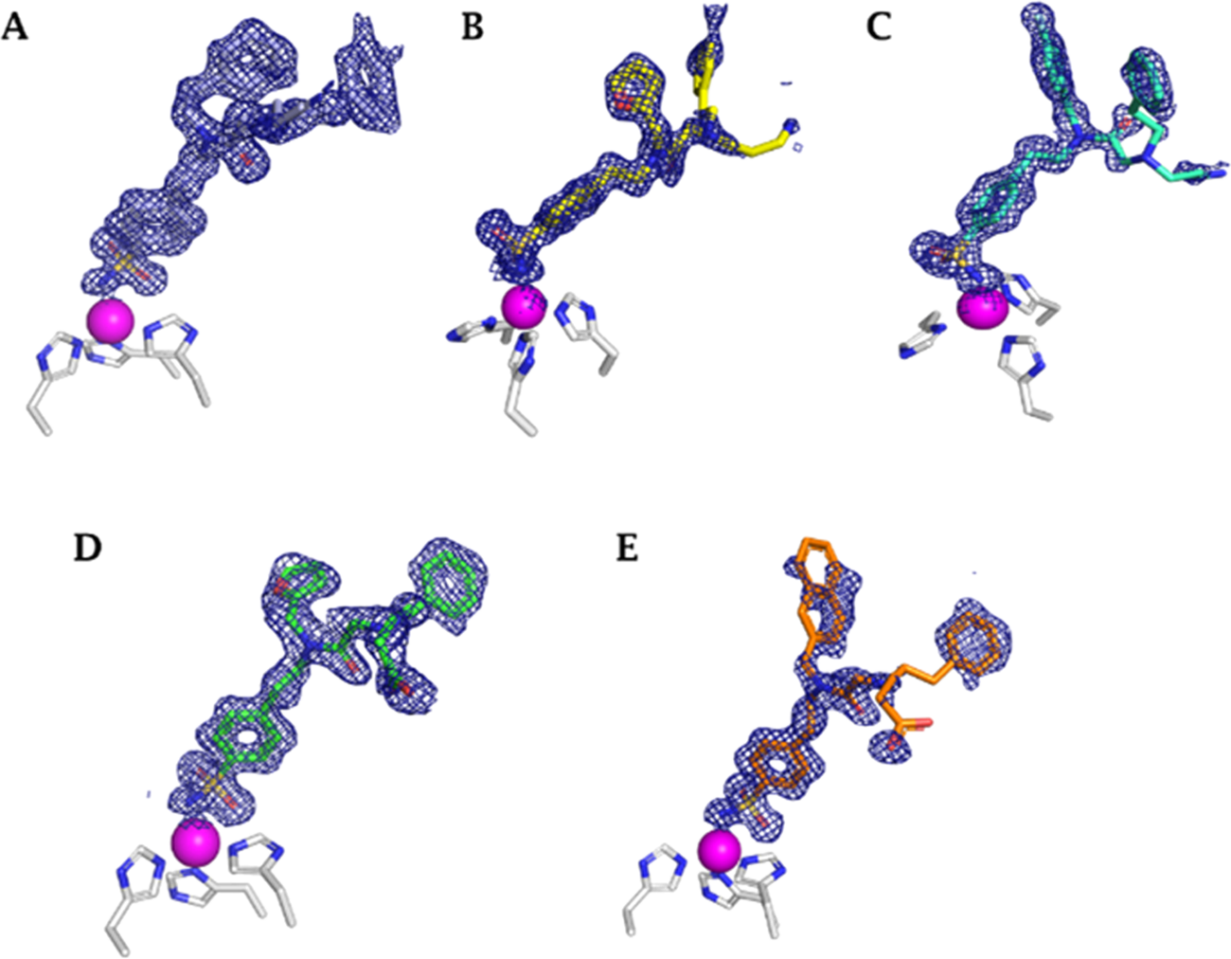
Electron densities of (A) **34**, (B) **41**, (C) **42**, (D) **46**, and (E) **48** in hCA II active site with a sigma of 1.0.

**Figure 6:**
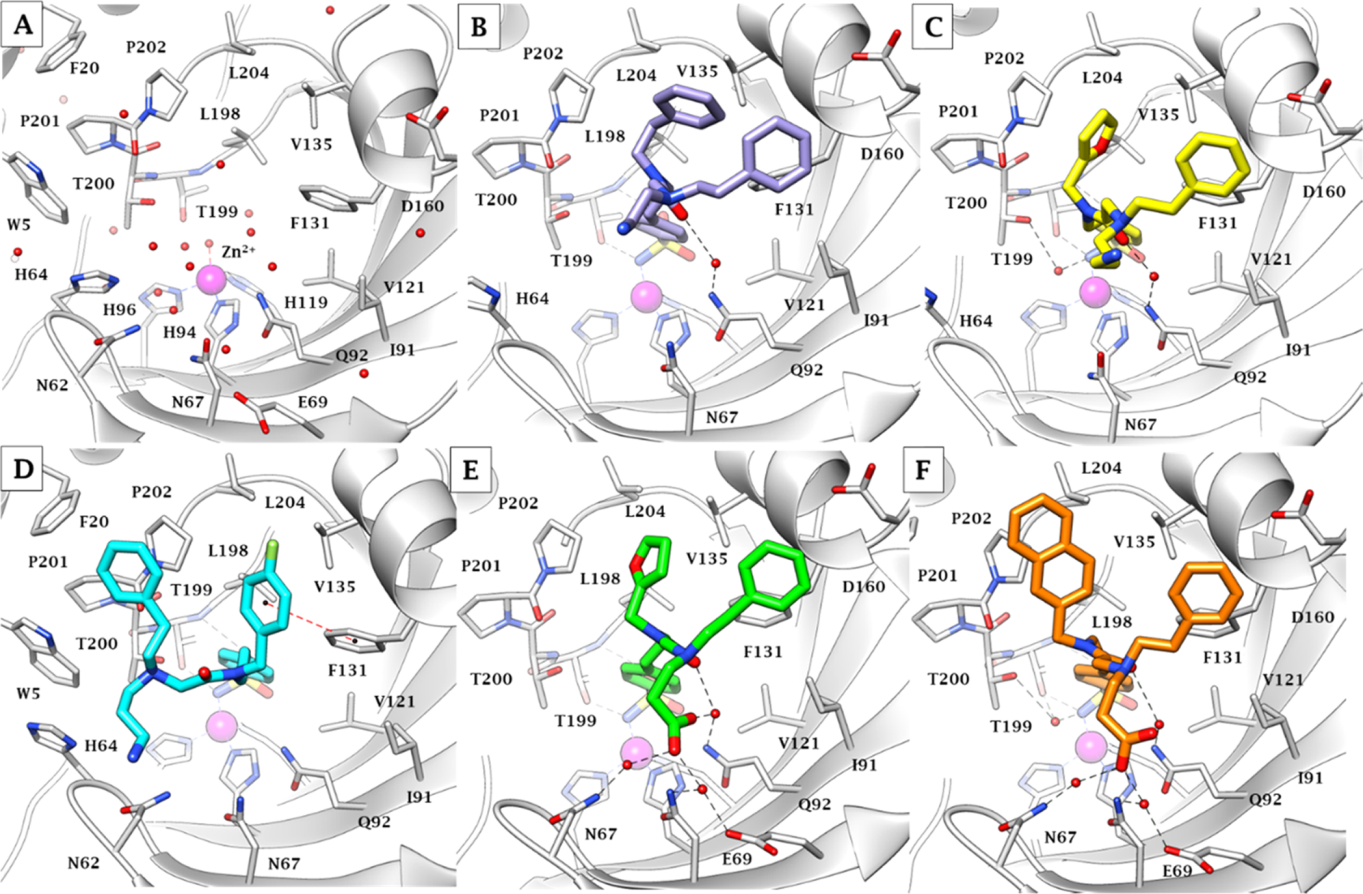
X-ray crystallography: active site view of hCA II in adduct with (A) no inhibitor (PDB 3KKX), (B) **34** (PDB 6WQ4), (C) **41** (PDB 6WQ5), (D) **42** (PDB 6WQ7), (E) **46** (PDB 6WQ8), and (F) **48** (PDB 6WQ9). H-bonds and π–π stackings are represented as black and red dashed lines, respectively. Water molecules involved in water-bridged H-bonds are shown as red spheres. Amino acids are labeled with one-letter symbols: D, Asp; E, Glu; F, Phe; H, His; I, Ile; L, Leu; N, Asn; P, Pro; Q, Gln; T, Thr; V, Val; W, Trp.

Co-crystallization of hCA II with selected three-tailed inhibitors resulted in solved structures with resolutions between 1.35 and 1.62 Å (Figures 4–6 and Table 2). For all of the inhibitors studied, the benzenesulfonamide was orientated with the zinc-binding group displacing the active site zinc-bound water (ZBW) and forming a hydrogen bond between the amide backbone of Thr199 and oxygen of sulfonamide (2.8–3.0 Å). Therefore, with the benzenesulfonamide binding in an identical manner, any differences in observed binding affinity most likely result from differences in the tail regions.

**Table 2:** X-ray Crystallography Data Collection and Refinement Statistics of Inhibitors Bound hCA II Crystal Structures^e^

inhibitor	**34**	**41**	**42**	**46**	**48**
PDB	6WQ4	6WQ5	6WQ7	6WQ8	6WQ9
space group	*P*21				
cell dimensions:	42.4, 41.5,	42.3, 41.4,	42.1, 41.3	42.4, 41.3,	42.3, 41.3,
*a*, *b*, *c*, β (Å, deg)	72.3, 104.3	72.3, 104.4	72.1, 104.3	72.4, 104.4	72.3, 104.4
resolution (Å)	29.19–1.35	25.34–1.30	25.28–1.30	28.75–1.41	21.13–1.30
highest-resolution shell (Å)	(1.40–1.35)	(1.35–1.30)	(1.35–1.30)	(1.46–1.41)	(1.35–1.30)
total reflections	9536	8627	8885	14 181	8927
*I*/σ(*I*)	16.3 (2.7)	14.5 (1.6)	15.5 (1.7)	12.5 (2.4)	20.6 (2.5)
redundancy	3.1 (2.2)	3.1 (2.2)	3.1 (1.9)	3.3 (3.1)	3.2 (2.3)
completeness (%)	98.0 (82.5)	95.8 (68.1)	97.4 (81.5)	99.4 (97.8)	94.0 (66.6)
*R*_sym_^a^	4.10 (25.6)	4.33 (49.0)	3.87 (40.7)	5.44 (39.3)	3.29 (35.7)
*R*_crys_^b^	15.4 (22.7)	16.0 (29.3)	16.0 (26.9)	14.6 (20.5)	14.9 (22.8)
Rf_ree_^c^	17.3 (25.2)	18.1 (31.7)	18.1 (30.5)	17. (22.4)	17.3 (28.5)
*R*_pim_^d^	2.67 (19.2)	2.82 (37.1)	2.53 (34.6)	3.52 (26.0)	2.12 (26.5)
# of atoms: protein, ligand, water	2049, 52, 209	2075, 44, 239	2076, 46, 239	2073, 87, 235	2080, 56, 248
protein residues	257	257	257	258	257
Ramachandran stats (%): favored, allowed	96.1, 3.9	96.9, 3.1	96.9, 3.1	96.1, 3.9	96.5, 3.5
avg. B-factors (Å^2^): main-,	13.9, 14.7	15.4, 16.5	16.4, 17.4	15.1, 16.5	14.9, 16.4
side chain, inhibitor, solvent	16.7, 21.9	25.7, 24.0	27.5, 24.5	29.3, 24.1	31.5, 25.2
RMSD for bond lengths, angles (Å, deg)	0.008, 1.05	0.008, 1.04	0.008, 1.04	0.009, 1.09	0.008, 1.07

a *R*__sym__ = (∑|*I* – ⟨*I*⟩|/∑⟨*I*⟩) × 100.

b *R*__cryst__ = (∑|*F*__o__ – *F*__c__|/∑|*F*__o__|) × 100.

c Rf__ree__ is calculated in the same way as *R*__cryst__ except it is for data omitted from refinement (5% of reflections for all data sets).

d *R*__pim__ = [(∑√1/*N* – 1)∑|*I* – ⟨*I*⟩|/∑⟨*I*⟩] × 100.

e Values in parentheses correspond to those of the highest-resolution shell.

s

Compound **34** showed a well-observed omit map electron density, indicating good binding with a high binding occupancy (PDB 6WQ4 and Figure 5B). The T_1_ phenethyl was accommodated in the lipophilic pocket lined by Val135, Leu198, Pro202, and Leu204, whereas the phenethyl in T_2_ lied above Phe131, forming contacts with the α-helix portion constituted by residues 130–136 (Figure 6B). A water-bridged H-bond took place between the ligand amide carbonyl group and Gln92 side chain NH_2_. The hydrophilic CN tail extended into bulk solvent.

Compound **41** exhibited a weaker observed omit map electron density around the phenethyl and aminopropyl tails (PDB 6WQ5 and Figure 5C). While the furyl ring took the place occupied by the T_1_ benzene ring of compound **34**, the phenethyl tail in T_2_ formed again interactions with Phe131 and residues nearby (Figure 6C). The amino group in T_3_ protonated at physiological pH was exposed to bulk solvent.

Compound **42** showed a weak observed omit map electron density at the end of the aminopropyl tail (PDB 6WQ7 and Figure 5D). The switch from a furyl (**41**) to a 4-F-benzyl (**42**) in T_1_ markedly shifted the tails of the ligand within the CA II active site, probably because the pocket hosting the furyl ring cannot accommodate additional steric hindrance. This did not occur with the T_1_ phenethyl group of **34** as the presence of an additional carbon unit allowed a torsion preserving a **41**-like binding mode. The 4-F-benzyl formed hydrophobic contacts with Val135, Leu198, and Phe131, and an edge-to-face π–π stacking with the latter residue benzene ring (Figure 6D). The T_2_ phenethyl of **42** lodged over the lipophilic portion composed of Trp5, Phe20, Pro201, and Pro202, while the protonated amino group in T_3_ was again exposed to bulk solvent.

Compound **46** showed a strong observed omit map electron density, which is indicative of a high binding occupancy (PDB 6WQ8 and Figure 5E). The T_1_ and T_2_ tails of the ligand adopted analogue positions within the active site to those of compound **41** (Figure 6E). The carboxylic tail was oriented toward the hydrophilic region within the active site, where the COOH, presumably as COO^–^, is involved in a water-mediated H-bond network with Asn62, Asn67, Glu69, and Gln92.

Compound **48** had a weaker observed omit map electron density near the carboxylic acid tail (PDB 6WQ9 and Figure 5F). As it occurred with compound **42**, the additional steric hindrance in T_1_ moved the naphthyl ring away from the pocket occupied by the furyl core of **41** (Figure 6F). Nonetheless, the intense H-bond network between the COO^–^ moiety in T_3_ and Asn62, Asn67, Glu69, and Gln92 prevented the T_2_/T_3_ branching N atom to move toward Trp5. This produced a switch between the positioning of T_1_ and T_2_ tails for **48** with respect to **42**. The naphthyl portion in T_1_ accommodated above the lipophilic pocket lined by Leu198, Pro201, Pro202, and Leu204, whereas the phenethyl in T_2_ interacted with Phe131 and other α-helix composing residues by van der Waals contacts.

It can be noted that the binding mode exhibited by compound **46** was the most efficient for promoting hCA II inhibition because of a 10-fold higher *K*_I_ (2.4 nM) than the second-best derivative among those co-crystallized (**42**, *K*_I_ of 30.4 nM). Considering the similar interactions observed for tails T_1_ and T_2_ with respect to compounds **34** and **41**, this enhanced efficacy might be consequent of the extended water-mediated H-bond network the carboxyethyl pendant formed with the hydrophilic portion of the binding cleft. Interestingly, the binding mode exhibited by **42**, though most departed from those of the other co-crystallized ligands, produced the second-best inhibition of hCA II. Swapping the furyl ring of **46** with the naphthyl of **48** significantly lowered the efficiency of the binding mode, as the bi-cycle cannot be accommodated in the Leu198, Pro201, Pro202, and Leu204 pocket and was partially exposed to bulk solvent. The exposure of the markedly less hydrophilic cyanoethyl tails of **34** to bulk solvent is the presumable reason for the drop of CA II inhibition exhibited by the ligand.

### *In Silico* Study

The crystallographic screening was complemented with docking calculations to also study hCA isoforms not included in the crystallographic study; hCA I (PDB 2NMX),^43^ hCA IV (PDB 1ZNC),^44^ and hCA XII (PDB 1JD0).^45^ The *in silico* study was performed on the single-tail derivatives **1–7** and, among **TTI**s, the most potent compounds against each isoform and co-crystallized ligands assembling a subset of seven derivatives (**34**, **39**, **41**, **42**, **46**, **48**, and **49**) and predicting their binding to hCAs I, IV and XII as well as CA II (PDB 5LJT)^46^ when missing (Figures S1–S6, Supporting Information). The binding orientations resulting from docking were refined with an MM-GBSA method simulating a water media (VSGB method) for improving the comparison with the crystallographic outcomes. The efficiency of the adopted protocol with three-tail compounds was validated by application to the crystallographic target/inhibitor adducts described above. Despite the absence of water molecules, crystallographic/simulated ligand RMSDs were computed below 1.0 Å, with the main deviation at the level of aliphatic tails (e.g., the carboxylate pendant in compound **46**; Figure S1, Supporting Information).

Predictably, derivatives **1–7** showed interactions within the hCA I, II, and XII active sites limited to a portion of the hydrophobic half of the cavity (Figure S2, Supporting Information). As a result, this produces inhibition profiles devoid of selectivity and thus promiscuous. The absence of a hydrophobic half in the active site of hCA IV led the tails of **1–7** toward alternative pockets according to the nature of the pendants, and on the whole, reduces the inhibition efficacy up to a micromolar range.

**Figure 7:**
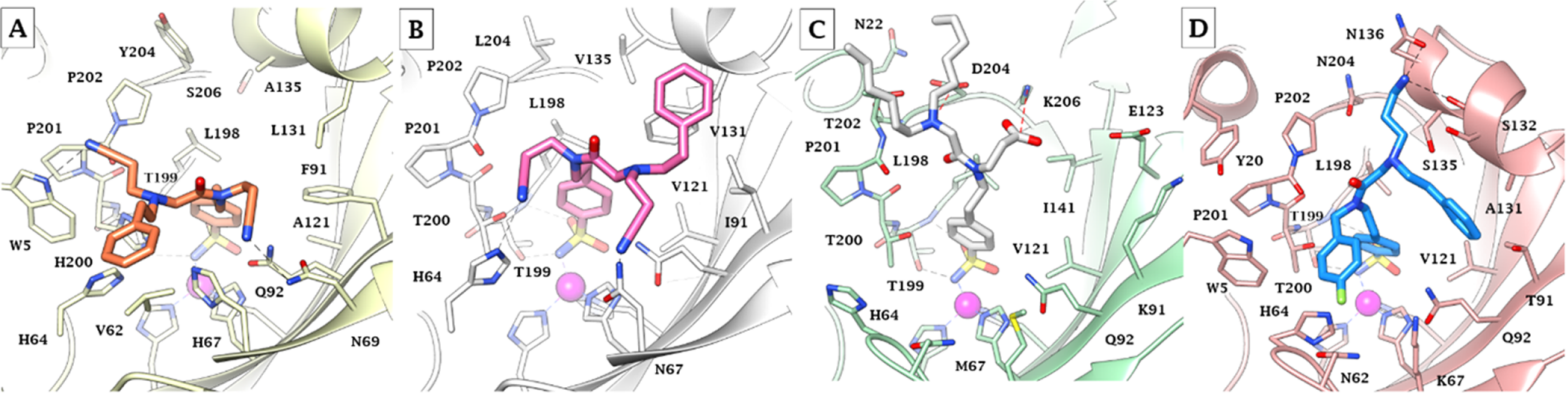
*In silico* predicted binding conformations for the adducts (A) **39**/hCA I, (B) **39**/hCA II, (C) **49**/hCA IV, and (D) **42**/hCA XII. H-bond and salt bridge interactions are depicted as black and red dashed lines, respectively.

Figure 7A,B depicts the predicted binding modes of **39** to hCA I and II, respectively, as the most active inhibitor against these two isoforms. HCA I shows a narrower active site than hCA II because of specific amino acid mutations such as Thr/His200, Asn/His67, Leu/Tyr204, and, mostly, Ile/Phe91 (Figure S3, Supporting Information). As the main result of the latter mutation, T_2_ and T_3_ are shifted toward the lipophilic pocket lined by Trp5, Val62 (solely present in hCA I), His64, and Pro201, where the cyanoethyl moiety receives a H-bond by Trp5 NH. The cyanoethyl in T_1_ engages interactions with the hydrophilic half of the binding cavity, among which forms a H-bond with Asn69 side chain NH_2_. As for hCA II, the tail of the ligand occupies on the whole a region nearer to the hydrophobic half of the active site. In fact, the phenethyl in T_2_, as observed in crystallography with similar ligands, lies above Phe131 interacting with residues 13–135 of the α-helix. The position of the two cyanoethyl portions is almost inverted compared to hCA I: the moiety in T_1_ receives H-bond by His64 NH, whereas the nitrile group in T3 is in H-bond distance with Asn67. As compound **39** uniquely possesses, among the selected derivatives (Figure S4, Supporting Information), two aliphatic, partially polar but nonprotic tails (cyanoethyl), it can be supposed a favorable complementarity with the narrow and rather lipophilic active sites of hCA I and II, which drives the most potent action here reported against the two ubiquitous isoforms (*K*_I_’s of 61.6 and 0.7 nM, respectively).

In fact, the greater steric hindrance produced by another phenethyl in T_1_ (compound **34**, Figures 6B and S4A, Supporting Information) lowered the inhibition potency by 10 and 500 times against hCA I and II, respectively. Solely the presence of a carboxyethyl tail in T_3_ of compound **46** (but not **48**, presumably because of the unwieldy naphthyl ring in T_1_) leads the inhibitor action against isoform I (*K*_I_ of 79.5 nM) and II (*K*_I_ of 2.4 nM) to the level of compound **39**, likely because of the interactions of the carboxylate with the hydrophilic portion of the binding cavity (Figures 6E,F and S4B, Supporting Information).

The active site of hCA IV is the most particular among those of hCAs as largely losing the hydrophibic/hydrophilic division common to most other catalytically active isoforms. In fact, α-helix 130–135 is absent and replaced by an extended loop which protrudes to bulk solvent. At the same time, the hydrophobic half of the binding cavity is replaced by a region rich in polar amino acids such as Lys91, Glu123, Thr202, Asp204, Lys206, and Glu138 (Figures 7C and S5, Supporting Information). As a result, this isoform is less inhibited by **TTI**s, with *K*_I_s above 100 nM, except for derivative **49**, that solely possesses a carboxylate function in T_1_. As shown in Figure 7C, the latter forms a salt bridge with Lys206, and this conformation also leads the protonated T_2_/T_3_ N branching atom in salt bridge with Asp204. Other ligands, such as **41**, **42**, and **48**, were also predicted to form salt bridges within the hCA IV active site (Figure S5, Supporting Information), but involving carboxylate or amine moieties in T_3_. As a result, the ligands adopt conformation, which do not allow the formation of two salt bridges with the polar pocket of the active site, as observed for **49**. As the latter shows a *K*_I_ of 45.8 nM despite two hexyl groups protruding to bulk solvent (Figure 7C), it can be supposed that their replacement with less lipophilic groups might even increase the inhibition efficiency of this membrane-associated CA.

Isoform hCA XII maintains an overall hydro/lipophilic partition in its wide active site (Phe/Ala131 with respect to hCA II), but specific mutations with respect to CA II, that are Asn/Lys67, Ile/Thr91, Gly/Ser132, Val/Ser135, and Leu/Asn204, significantly enhance the hydrophilicity of the binding cavity (Figures 7D and S6, Supporting Information). It should be noted that compound **42** shows the unique subnanomolar *K*_I_ value against a tumor-associated CA (*K*_I_ of 0.6 nM against CA XII). The peculiar active site architecture of hCA XII indeed drives a favorable disposition of the three tails of the ligands: the T_1_ 4-F-phenyl accommodates in the pocket lined by Trp5, His64, Asn62, and Lys67; the T_2_ phenethyl lies over the most lipophilic cleft of the binding pocket, made by Val121, Thr91, and Ala131; and the propylamine pendant in T_3_ is involved in a bifurcated H-bond system with the side-chain Asn136 and Ser132 backbone CO (Figure 7D). In contrast, compound **41**, having a furyl ring in place of the 4-F-phenyl of **42**, exhibits a very different binding orientation in the hCA XII active site (Figure S6, Supporting Information), as it occurred with CA II as well (Figures 6 and 7).

### Intraocular Pressure-Lowering Activity

**Figure 8:**
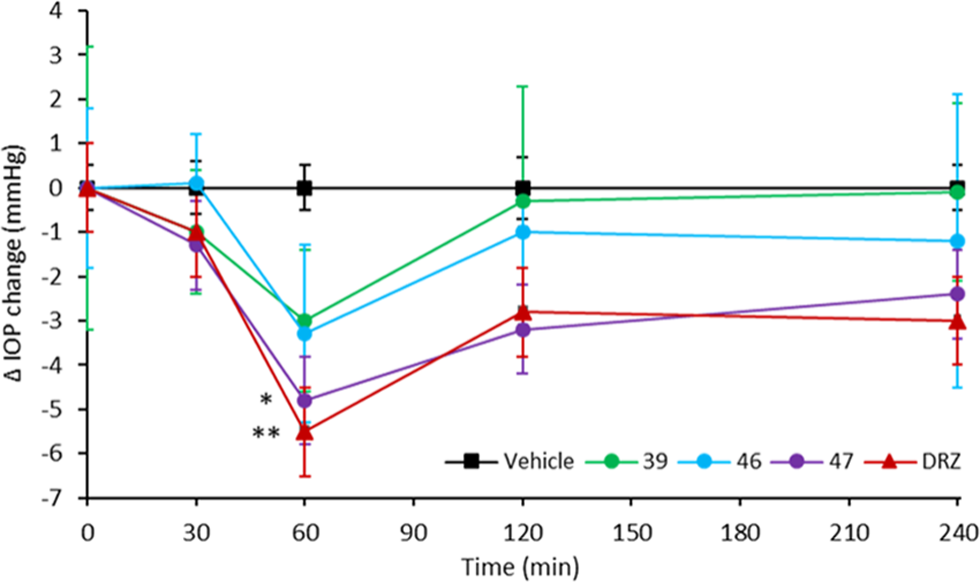
Drop of intraocular pressure (ΔIOP, mmHg) versus time (min) in hypertonic saline-induced ocular hypertension in rabbits, treated with 50 μL of 1% solution of compounds **39**, **46**, and **47**, and **DRZ** as the standard. Hydroxypropylcellulose at 0.05% was used as vehicle. Data are analyzed with two-way analysis of variance (ANOVA) followed by Bonferroni multiple comparison test. *p < 0.05 **47** vs vehicle at 60′; **p < 0.01 **DRZ** vs vehicle at 60′.

For a first pharmacological application of the proposed approach, we selected the inhibitors showing the best concomitant action against hCA II, IV, and XII (**39**, **46**, and **47**) for evaluating their intraocular pressure (IOP)-lowering activity in a rabbit model of glaucoma (Figure 8). The compounds showed sufficient water solubility to be formulated as 1% eye drops and **DRZ** hydrochloride 1% were used as reference compound and hydroxypropylcellulose 0.05% as vehicle in the experimental setting. The compounds were formulated and administered as 1% eye drops to rabbits with high IOP, induced by the injection of 0.05 mL of hypertonic saline solution (5% in distilled water) into the vitreous of both eyes. As depicted in Figure 4, at 30 min post-instillation, only compounds **39** and **47** decreased the IOP by 1.0 and 1.3 mmHg, respectively, such as **DRZ** (−1.0 mmHg), while **46** was inactive. At 60 min after administration, all compounds triggered the maximum IOP reduction, where **39** and **46** showed maximal IOP-lowering activities of 3.0 and 3.3 mmHg, respectively. Instead, compound **47** resulted the most effective, decreasing the IOP of 4.8 mmHg in a comparable manner of **DRZ** (−5.4 mmHg). After 120 min, a decrease of the effect was observed for all compounds with **39** and **46** that decreased IOP by 0.3 and 1.0 mmHg, while the standard **DRZ** showed to be less effective than **47** (−3.2 mmHg) with an IOP reduction of 2.8 mmHg. Uniquely, compounds **46** (−1.2 mmHg) and **47** (−2.4 mmHg) protracted their action at 240 min post-instillation, whereas compound **39** was inactive. In particular, **47** showed a similar profile to the standard **DRZ** (−3.0 mmHg).

## Conclusions

The tail approach was proposed already in 1999 and progressively developed with a variety of chemical scaffolds up to the first report of dual-tail design in 2015 to target both the hydrophobic and hydrophilic halves of hCAs active sites. Such an undoubtedly favorable approach in the field of hCAs is still the main strategy used for obtaining CAIs and led us to propose its further development by the incorporation of three tails onto a benzenesulfonamide CA inhibitory scaffold. In fact, we deem the simple hydrophobic/hydrophilic division of hCAs binding pocket not totally sufficient anymore because of many accessory pockets existing in each hCA isoform. This proof-of-concept study reported here was carried out by the design and synthesis of 32 benzenesulfonamide derivatives of the **TTI** type (Figure 3) screened against a first set of hCAs that are I, II, IV, and XII, and comparing the results with the corresponding single-tail derivatives **1–7** (Table 1).

Our results showed that the development of **1–7** upon inclusion of two other tails to give compounds **18–50** significantly affected the inhibition profiles in terms of potency and selectivity of action. On the whole, it should be noted that the inclusion of three lipophilic tails in the **TTI** structure, such as in compounds **18–32**, did not produce noteworthy outcomes in terms of potency and selectivity against the tested hCAs, with very flat SAR within the subset, except for a few exceptions. In contrast, increasing the polarity of at least one tail (starting from compound **33**) resulted in a great variability of potencies and selectivities according to the type of tails included in T_1_, T_2_, and T_3_.

The structural study made by X-ray crystallography with hCA II and *in silico* tools with the other isozymes pointed out the limited and almost superimposable interactions that the tail of **1–7** can establish within the CAs active site. In contrast, we demonstrated that the **TTI** derivatives show a greater occupancy of the binding cavities with a great variability among isoforms that contribute to the development of improved selectivity of action.

Structural studies and SAR analysis showed how different tail combinations can distinctly promote the binding of benzenesulfonamide derivatives to the various hCAs active site. As an outcome of this preliminary investigation, we can infer that inhibition of hCA I and II, possessing narrow and markedly lipophilic active sites, can be promoted by inclusion of a lipophilic tail and two small half-polarity pendants in the **TTI** structure (e.g., compound **39**) or alternatively two not too bulky tails and a polar one (e.g., compound **46**). The marked polarity of hCA IV active site makes a significantly polar tail in T_1_ (nearby the main CA inhibitory scaffold) necessary to attain a low nanomolar inhibition (e.g., compound **49**). HCA XII and its wide hydrophobic binding pocket better accommodate almost all ligands with respect to hCA II, and thus most tail combinations produce efficient inhibition of the isozyme. The combination of lipophilic and polar tails coexisting with a medium polarity one even led to a subnanomolar hCA XII inhibitor (compound **42**).

For a first pharmacological application of the proposed approach, three **TTI**s were selected because of their potent and concomitant inhibition of hCA II, IV, and XII (CAs implicated in glaucoma) and were assessed *in vivo* in a rabbit model of the disease. Compound **47** showed the capability of lowering IOP as efficiently as the clinically used **DRZ** up to 120 min post-administration.

The outcomes of this proof-of-concept study represent a firm starting point for optimizing the general **TTI** design as well as to produce a wider set of tail combinations to even improve the ligand/isoforms matching in search of new CAI candidates in the treatment of spreading diseases such as glaucoma, tumors, neuropathic pain, and inflammation. It should be stressed that an analogous approach might be extended to other multi-isoform metalloenzymes to improve the outcomes in terms of selectivity of action.

## Experimental Section

### Chemistry

Anhydrous solvents and all reagents were purchased from Sigma-Aldrich, Fluorochem, and TCI Chemicals. All reactions involving air- or moisture-sensitive compounds were performed under a nitrogen atmosphere using dried glassware, and syringes were used to transfer solutions. Nuclear magnetic resonance (^1^H NMR, ^13^C NMR) spectra were recorded using a Bruker Advance III 400 MHz spectrometer in DMSO-*d*_6_. Chemical shifts are reported in parts per million (ppm), and the coupling constants (*J*) are expressed in hertz (Hz). Splitting patterns are designated as follows: s, singlet; d, doublet; t, triplet; q, quadruplet; m, multiplet; bs, broad singlet; dd, double of doublets. The assignment of exchangeable protons (O*H* and N*H*) was confirmed by the addition of D_2_O. Two tautomeric forms of the amide bond were detected for compounds **8–50**, which partially double the signals in the ^1^H and ^13^C NMR spectra. Analytical thin-layer chromatography (TLC) was carried out on Sigma-Aldrich silica gel F-254 plates. Flash chromatography purifications were performed on Sigma-Aldrich silica gel 60 (230–400 mesh ASTM) as the stationary phase, and ethyl acetate/n-hexane or MeOH/DCM was used as eluents. Melting points (mp) were measured in open capillary tubes with a Gallenkamp MPD350.BM3.5 apparatus and are uncorrected.

Compounds **1–7** and **18–50** were ≥95% pure. The purity of the final compounds was determined by HPLC analysis performed using an Agilent 1200 Series equipped with an autosampler, a binary pump system, and a diode array detector (DAD). The column used was a Luna PFP with 30 mm length, 2 mm internal diameter, and 3 μm particle size (Phenomenex, Bologna, Italy) at a constant flow of 0.25 mL min^–1^, employing a binary mobile phase elution gradient. The eluents used were 10 mM formic acid and 5 mM ammonium formate in an mQ water solution (solvent A) and 10 mM formic acid and 5 mM ammonium formate in methanol (solvent B) according to the elution gradient as follows: initial at 90% solvent A, which was then decreased to 10% in 8 min, kept for 3 min, returned to initial conditions in 0.1 min, and maintained for 3 min for reconditioning, to a total run time of 14 min. The stock solution of each analyte was prepared in methanol at 1.0 mg mL^–1^ and stored at 4 °C. The sample solution of the analyte was freshly prepared by diluting its stock solution up to a concentration of 10 μg mL^–1^ in a mixture of mQ water:methanol 50:50 (v/v), and 5 μL was injected into the HPLC system. The solvents used in HPLC measurements were methanol (Chromasolv grade), purchased from Sigma-Aldrich (Milan, Italy), and mQ water 18 MΩ cm, obtained from Millipore’s Simplicity system (Milan, Italy).

The high-resolution mass spectrometry (HRMS) analysis was performed with a Thermo Finnigan LTQ Orbitrap mass spectrometer equipped with an electrospray ionization (ESI) source. The analysis was carried out by introducing, via a syringe pump at 10 μL min^–1^, the sample solution (1.0 μg mL^–1^ in mQ water/acetonitrile 50:50), and it acquired the signal of the positive ions. These experimental conditions allow the monitoring of protonated molecules of the studied compounds ([M + H]^+^ species) that were measured with a proper dwell time to achieve 60 000 units of resolution at full width at half-maximum (FWHM). Elemental compositions of compounds were calculated on the basis of their measured accurate masses, accepting only results with an attribution error less than 2.5 ppm and a noninteger RDB (double bond/ring equivalents) value, to consider only the protonated species.^47^ None of the screened derivatives reported PAINS alerts determined by SwissADME server (www.swissadme.ch).

### General Synthesis Procedures for Preparation of 4-(2-(arylalkyl)aminoethyl)benzenesulfonamides (**1–7**)

*Procedure* 1: To a solution of 4-(2-aminoethyl)benzenesulfonamide (9.99 mmol, 1.0 equiv) in dry MeOH (40 mL), the appropriate aldehyde (1.1 equiv) was added and the mixture was heated at reflux temperature under stirring for 0.5–4 h. Sodium borohydride (1.6 equiv) was added portionwise at 0 °C, and the reaction mixture was stirred at reflux temperature for 0.5–3 h. The solvent was evaporated under *vacuum*, and water was added (25 mL). pH was taken to 7 with 1 M HCl. The suspension was filtered, and the collected powder was purified by flash silica chromatography (5% MeOH in DCM) to give compounds **1–5**.

*Procedure* 2: To a solution of 4-(2-aminoethyl)benzenesulfonamide (9.99 mmol, 1.0 equiv) in dry DMF (5 mL), triethylamine (1.2 equiv) and the appropriate halide (1.1 equiv) were added at room temperature, and the mixture was stirred at room temperature for 0.5 h (**6**) or 60 °C for 8 h (**7**). The reaction mixture was quenched by addition of water (20 mL) and extracted with DCM (30 mL × 3). The organic layer was collected, washed with brine (40 mL × 3), dried over Na_2_SO_4_, filtered, and evaporated under *vacuum* to give compounds **6–7** as powders.

#### 4-(2-(Benzylamino)ethyl)benzenesulfonamide (**1**)^48^

Compound **1** was obtained according to the general procedure 1 earlier reported using 4-(2-aminoethyl)benzenesulfonamide (9.99 mmol, 1.0 equiv) and benzaldehyde (1.1 equiv) in dry MeOH (40 mL). The reaction mixture was initially stirred at reflux temperature for 4 h, and after the addition of sodium borohydride (1.6 equiv), it was stirred at reflux temperature for another 2 h. Yield 96%; mp 173–175 °C; silica gel TLC *R*_*f*_ 0.08 (TFA/MeOH/DCM 3/5/92% v/v). δH (400 MHz, DMSO-*d*_6_): 7.76 (d, *J* = 8.1 Hz, 2H, Ar-*H*), 7.42 (m, 7H, Ar-*H*), 7.32 (s, 2H, exchange with D_2_O, SO_2_N*H*_2_, overlap with signal at 7.42), 4.04 (s, 2H, C*H*_2_), 3.07 (m, 2H, C*H*_2_), 2.97 (m, 2H, C*H*_2_). δC (100 MHz, DMSO-*d*_6_): 145.87, 142.74, 141.80, 129.99, 129.05, 128.86, 127.46, 126.55, 53.77, 50.91, 36.50. ESI-MS (*m*/*z*) [M + H]^+^: calcd for C_15_H_19_N_2_O_2_S 291.1; found 291.2.

#### 4-(2-((4-Nitrobenzyl)amino)ethyl)benzenesulfonamide (**2**)

Compound **2** was obtained according to the general procedure 1 earlier reported using 4-(2-aminoethyl)benzenesulfonamide (9.99 mmol, 1.0 equiv) and 4-nitrobenzaldehyde (1.1 equiv) in dry MeOH (40 mL). The reaction mixture was initially stirred at reflux temperature for 1 h, and after the addition of sodium borohydride (1.6 equiv), it was stirred at reflux temperature for 3 h. Yield 94%; mp 166–168 °C; silica gel TLC *R*_*f*_ 0.17 (TFA/MeOH/DCM 3/5/92% v/v). δH (400 MHz, DMSO-*d*_6_): 8.16 (d, *J* = 8.6 Hz, 2H, Ar-*H*), 7.72 (d, *J* = 8.2 Hz, 2H, Ar-*H*), 7.57 (d, *J* = 8.6 Hz, 2H, Ar-*H*), 7.39 (d, *J* = 8.2 Hz, 2H, Ar-*H*), 7.26 (s, 2H, exchange with D_2_O, SO_2_N*H*_2_), 3.84 (s, 2H, C*H*_2_), 2.82 (m, 2H, C*H*_2_), 2.73 (m, 2H, C*H*_2_), 2.40 (bs, 1H, exchange with D_2_O, N*H*). δC (100 MHz, DMSO-*d*_6_): 150.49, 147.34, 145.84, 142.88, 130.12, 129.84, 126.69, 124.29, 53.05, 51.00, 36.63. ESI-MS (*m*/*z*) [M + H]^+^: calcd for C_15_H_18_N_3_O_4_S 336.1; found 336.1.

#### 4-(2-((4-Fluorobenzyl)amino)ethyl)benzenesulfonamide (**3**)

Compound **3** was obtained according to the general procedure 1 earlier reported using 4-(2-aminoethyl)benzenesulfonamide (9.99 mmol, 1.0 equiv) and 4-fluorobenzaldehyde (1.1 equiv) in dry MeOH (40 mL). The reaction mixture was initially stirred at reflux temperature for 2 h, and after the addition of sodium borohydride (1.6 equiv), it was stirred at reflux temperature for another 2 h. Yield 95%; mp 145–147 °C; silica gel TLC *R*_*f*_ 0.21 (TFA/MeOH/DCM 3/5/92% v/v). δH (400 MHz, DMSO-*d*_6_): 7.73 (d, *J* = 8.2 Hz, 2H, Ar-*H*), 7.38 (m, 4H, Ar-*H*), 7.28 (s, 2H, exchange with D_2_O, SO_2_N*H*_2_, overlap with signal at 7.38), 7.12 (t, *J* = 8.8 Hz, 2H, Ar-*H*), 3.73 (s, 2H, C*H*_2_), 2.79 (m, 4H, 2 × C*H*_2_). δF (376 MHz, DMSO-*d*_6_): −116.18. δC (100 MHz, DMSO-*d*_6_): 145.61, 142.94, 131.06, 130.98, 130.10, 126.73, 115.96, 115.75, 52.74, 50.62, 36.18. ESI-MS (*m*/*z*) [M + H]^+^: calcd for C_15_H_18_FN_2_O_2_S 309.1; found 309.1.

#### 4-(2-((Naphthalen-2-ylmethyl)amino)ethyl)benzenesulfonamide (**4**)

Compound **4** was obtained according to the general procedure 1 earlier reported using 4-(2-aminoethyl)benzenesulfonamide (9.99 mmol, 1.0 equiv) and 2-naphthaldehyde (1.1 equiv) in dry MeOH (40 mL). The reaction mixture was initially stirred at reflux temperature for 0.5 h, and after the addition of sodium borohydride (1.6 equiv), it was stirred at reflux temperature for another 0.5 h. Yield 86%; mp 186–188 °C; silica gel TLC *R*_*f*_ 0.04 (TFA/MeOH/DCM 3/5/92% v/v). δH (400 MHz, DMSO-*d*_6_): 7.85 (m, 2H, Ar-*H*), 7.76 (s, 1H, Ar-*H*), 7.70 (d, *J* = 8.2 Hz, 2H, Ar-*H*), 7.48 (m, 4H, Ar-*H*), 7.38 (d, *J* = 8.1 Hz, 2H, Ar-*H*), 7.19 (s, 2H, exchange with D_2_O, SO_2_N*H*_2_), 3.87 (s, 2H, C*H*_2_), 2.79 (m, 4H, 2 × C*H*_2_), 2.21 (bs, 1H, exchange with D_2_O, N*H*). δC (100 MHz, DMSO-*d*_6_): 145.85, 142.88, 142.83, 139.55, 133.94, 133.08, 130.05, 128.57, 128.48, 127.73, 126.97, 126.88, 126.58, 126.40, 53.85, 51.01, 36.57. ESI-MS (*m*/*z*) [M + H]^+^: calcd for C_19_H_21_N_2_O_2_S 341.1; found 341.1.

#### 4-(2-((Furan-2-ylmethyl)amino)ethyl)benzenesulfonamide (**5**)

Compound **5** was obtained according to the general procedure 1 earlier reported using 4-(2-aminoethyl)benzenesulfonamide (9.99 mmol, 1.0 equiv) and 2-furaldehyde (1.1 equiv) in dry MeOH (40 mL). The reaction mixture was initially stirred at reflux temperature for 4 h, and after the addition of sodium borohydride (1.6 equiv), it was stirred at reflux temperature for another 3h. Yield 88%; mp 133–135 °C; silica gel TLC *R*_*f*_ 0.19 (TFA/MeOH/DCM 3/5/92% v/v). δH (400 MHz, DMSO-*d*_6_): 7.71 (d, *J* = 8.3 Hz, 2H, Ar-*H*), 7.56–7.49 (m, 1H, Ar-*H*), 7.37 (d, *J* = 8.3 Hz, 2H, Ar-*H*), 7.24 (s, 2H, exchange with D_2_O, SO_2_N*H*_2_), 6.35 (dd, *J* = 3.1, 1.9 Hz, 1H, Ar-*H*), 6.20 (d, *J* = 3.1 Hz, 1H, Ar-*H*), 3.67 (s, 2H, C*H*_2_), 2.75 (m, 4H, 2 × C*H*_2_), 2.04 (bs, 1H, exchange with D_2_O, N*H*). δC (100 MHz, DMSO-*d*_6_): 155.36, 145.73, 142.62, 129.93, 126.49, 126.48, 111.13, 107.47, 50.67, 46.21, 36.29. ESI-MS (*m*/*z*) [M + H]^+^: calcd for C_13_H_17_N_2_O_3_S 281.1; found 281.1.

#### 4-(2-((2-Cyanoethyl)amino)ethyl)benzenesulfonamide (**6**)

Compound **6** was obtained according to the general procedure 2 earlier reported using 4-(2-aminoethyl)benzenesulfonamide (9.99 mmol, 1.0 equiv) and 3-chloropropionitrile (1.1 equiv) in dry DMF (5 mL) and at rt stirring for 0.5 h. Yield 85%; mp 85–87 °C; silica gel TLC *R*_*f*_ 0.15 (TFA/MeOH/DCM 3/5/92% v/v). δH (400 MHz, DMSO-*d*_6_): 7.72 (d, *J* = 8.0 Hz, 2H, Ar-*H*), 7.41 (d, *J* = 8.0 Hz, 2H, Ar-*H*), 7.27 (s, 2H, exchange with D_2_O, SO_2_N*H*_2_), 2.76 (m, 6H, 3 × CH_2_), 2.57 (t, *J* = 6.6 Hz, 2H, C*H*_2_). δC (100 MHz, DMSO-*d*_6_): 145.72, 142.88, 130.14, 126.68, 121.19, 50.83, 45.66, 36.59, 18.88. ESI-MS (*m*/*z*) [M + H]^+^: calcd for C_11_H_16_N_3_O_2_S 254.1; found 254.0.

#### 4-(2-(Phenethylamino)ethyl)benzenesulfonamide (**7**)

Compound **7** was obtained according to the general procedure 2 earlier reported using 4-(2-aminoethyl)benzenesulfonamide (9.99 mmol, 1.0 equiv) and (2-bromoethyl)benzene (1.1 equiv) in dry DMF (5 mL) and at 60 °C stirring for 8 h. Yield 73%; mp 213–215 °C; silica gel TLC *R*_*f*_ 0.02 (TFA/MeOH/DCM 3/5/92% v/v). δH (400 MHz, DMSO-*d*_6_): 7.78 (d, *J* = 8.2 Hz, 2H, Ar-*H*), 7.44 (d, *J* = 8.2 Hz, 2H, Ar-*H*), 7.34 (m, 4H, Ar-*H*), 7.26 (s, 2H, exchange with D_2_O, SO_2_N*H*_2_, overlap with signal at 7.25), 7.25 (m, 1H, Ar-*H*), 2.89 (m, 8H, 4 × C*H*_2_). δC (100 MHz, DMSO-*d*_6_): 145.71, 142.27, 141.02, 130.04, 129.47, 129.26, 126.88, 126.59, 51.35, 50.91, 36.29, 36.05. ESI-MS (*m*/*z*) [M + H]^+^: calcd for C_16_H_21_N_2_O_2_S 305.1; found 305.1.

### General Synthesis Procedure of Chloro-amides (**8–17**)

To a suspension of 4-(2-(arylalkyl)aminoethyl)benzenesulfonamide **1–7** (6.89 mmol, 1.0 equiv) and K_2_CO_3_ (1.2 equiv) in acetone (40 mL) cooled to 0 °C, the appropriate chloroacylchloride (1.2 equiv) was added dropwise and the mixture was stirred for 0.5 h. The solvent was evaporated under *vacuum*, then slush (50 mL) was added, and the basic suspension was neutralized with 1 M HCl. The precipitate was collected by filtration and purified with flash chromatography (1% MeOH in DCM) to give compounds **8–17**.

#### *N*-Benzyl-2-chloro-*N*-(4-sulfamoylphenethyl)acetamide (**8**)

Compound **8** was obtained according to the general procedure earlier reported using 4-(2-(benzylamino)ethyl)benzenesulfonamide **1** and 2-chloroacetyl chloride (1.2 equiv). Yield 91%; mp 122–124 °C; silica gel TLC *R*_*f*_ 0.32 (TFA/MeOH/DCM 3/5/92% v/v). δH (400 MHz, DMSO-*d*_6_): 7.74 (t, *J* = 8.7 Hz, 2H, Ar-*H*), 7.35 (m, 7H, Ar-*H*), 7.27 (s, 2H, exchange with D_2_O, SO_2_N*H*_2_, overlap with signal at 7.35), 4.58 (s, 2H, C*H*_2_), 4.42 (s, 1.2H, C*H*_2_), 4.40 (s, 0.8H, C*H*_2_), 3.46 (m, 2H, C*H*_2_), 2.96 (m, 1H, C*H*_2_), 2.82 (m, 1H, C*H*_2_). δC (100 MHz, DMSO-*d*_6_): 167.23, 144.15, 143.63, 143.50, 143.28, 138.55, 137.89, 130.43, 130.15, 129.83, 129.55, 128.62, 128.23, 128.14, 126.89, 49.33, 49.08, 48.26, 43.19, 43.11, 34.80, 33.62. ESI-MS (*m*/*z*) [M + H]^+^: calcd for C_17_H_20_ClN_2_O_3_S 367.1; found 367.0.

#### *N*-Benzyl-3-chloro-*N*-(4-sulfamoylphenethyl)propanamide (**9**)

Compound **9** was obtained according to the general procedure earlier reported using 4-(2-(benzylamino)ethyl)benzenesulfonamide **1** and 3-chloropropionyl chloride (1.2 equiv). Yield 93%; mp 151–153 °C; silica gel TLC *R*_*f*_ 0.36 (TFA/MeOH/DCM 3/5/92% v/v). δH (400 MHz, DMSO-*d*_6_): 7.73 (t, *J* = 7.2 Hz, 2H, Ar-*H*), 7.32 (m, 7H, Ar-*H*), 7.25 (s, 2H, exchange with D_2_O, SO_2_N*H*_2_, overlap with signal at 7.32), 4.56 (s, 2H, C*H*_2_), 3.79 (m, 2H, C*H*_2_), 3.47 (m, 3H, 2 × C*H*_2_), 2.86 (m, 3H, 2 × C*H*_2_). δC (100 MHz, DMSO-*d*_6_): 170.61, 170.55, 144.41, 143.79, 143.39, 143.13, 138.89, 138.40, 130.43, 130.19, 129.82, 129.52, 128.65, 128.43, 128.16, 127.65, 126.89, 126.85, 51.60, 48.93, 48.76, 48.41, 41.94, 41.68, 36.60, 36.05, 34.93, 34.05. ESI-MS (*m*/*z*) [M + H]^+^: calcd for C_18_H_22_ClN_2_O_3_S 381.1; found 381.0.

#### 2-Chloro-*N*-(4-nitrobenzyl)-*N*-(4-sulfamoylphenethyl)acetamide (**10**)

Compound **10** was obtained according to the general procedure earlier reported using 4-(2-((4-nitrobenzyl)amino)ethyl)benzenesulfonamide **2** and 2-chloroacetyl chloride (1.2 equiv). Yield 89%; mp 204–206 °C; silica gel TLC *R*_*f*_ 0.28 (TFA/MeOH/DCM 3/5/92% v/v). δH (400 MHz, DMSO-*d*_6_): 8.20 (d, *J* = 8.7 Hz, 2H, Ar-*H*), 7.77 (m, 3H, Ar-*H*), 7.49 (m, 4H, Ar-*H*), 7.31 (s, 2H, exchange with D_2_O, SO_2_N*H*_2_), 4.76 (s, 0.6 H, C*H*_2_), 4.71 (s, 1.4 H, C*H*_2_), 4.47 (s, 1.4 H, C*H*_2_), 4.38 (s, 0.6 H, C*H*_2_), 3.55 (m, 2H, C*H*_2_), 2.97 (m, 1.5H, C*H*_2_), 2.84 (m, 0.5H, C*H*_2_). δC (100 MHz, DMSO-*d*_6_): 166.50, 164.81, 146.66, 145.78, 144.58, 142.46, 142.41, 140.51, 131.32, 131.13, 129.42, 129.13, 128.40, 128.17, 125.99, 125.82, 123.81, 123.57, 50.32, 49.22, 48.96, 48.16, 47.53, 44.25, 42.06, 33.84. ESI-MS (*m*/*z*) [M + H]^+^: calcd for C_17_H_19_ClN_3_O_5_S 412.1; found 412.0.

#### 2-Chloro-*N*-(4-fluorobenzyl)-*N*-(4-sulfamoylphenethyl)acetamide (**11**)

Compound **11** was obtained according to the general procedure earlier reported using 4-(2-((4-fluorobenzyl)amino)ethyl)benzenesulfonamide **3** and 2-chloroacetyl chloride (1.2 equiv). Yield 86%; mp 167–169 °C; silica gel TLC *R*_*f*_ 0.30 (TFA/MeOH/DCM 3/5/92% v/v). δH (400 MHz, DMSO-*d*_6_): 7.74 (t, *J* = 8.6 Hz, 2H, Ar-*H*), 7.32 (m, 6H, Ar-*H*), 7.30 (s, 2H, exchange with D_2_O, SO_2_N*H*_2_, overlap with signal at 7.32), 4.56 (s, 2H, C*H*_2_), 4.41 (s, 2H, C*H*_2_), 3.45 (m, 2H, C*H*_2_), 2.95 (t, *J* = 7.4 Hz, 1.2H, C*H*_2_), 2.78 (m, 0.8H, C*H*_2_). δF (376 MHz, DMSO-*d*_6_): −114.96, −115.41. δC (100 MHz, DMSO-*d*_6_): 167.31, 143.60, 143.47, 142.79, 134.74, 130.74, 130.66, 130.44, 130.15, 129.80, 126.89, 116.68, 116.39, 116.18, 51.23, 49.32, 48.44, 48.10, 43.15, 43.07, 34.79, 33.57. ESI-MS (*m*/*z*) [M + H]^+^: calcd for C_17_H_19_ClFN_2_O_3_S 385.0; found 385.0.

#### 2-Chloro-*N*-(naphthalen-2-ylmethyl)-*N*-(4-sulfamoylphenethyl)acetamide (**12**)

Compound **12** was obtained according to the general procedure earlier reported using 4-(2-((naphthalen-2-ylmethyl)amino)ethyl)benzenesulfonamide **4** and 2-chloroacetyl chloride (1.2 equiv). Yield 54%; mp 181–183 °C; silica gel TLC *R*_*f*_ 0.34 (TFA/MeOH/DCM 3/5/92% v/v). δH (400 MHz, DMSO-*d*_6_): 7.91 (m, 3H, Ar-*H*), 7.73 (m, 3H, Ar-*H*), 7.40 (m, 5H, Ar-*H*), 7.30 (s, 2H, exchange with D_2_O, SO_2_N*H*_2_, overlap with signal at 7.40), 4.75 (s, 2H, C*H*_2_), 4.47 (s, 1.2H, C*H*_2_), 4.46 (s, 0.8H, C*H*_2_), 3.52 (t, *J* = 7.5 Hz, 2H, C*H*_2_), 3.01 (m, 2H, C*H*_2_), 2.84 (m, 2H, C*H*_2_). δC (100 MHz, DMSO-*d*_6_): 167.33, 144.15, 143.64, 143.50, 143.29, 136.15, 135.55, 134.99, 134.01, 133.95, 133.44, 133.32, 130.44, 130.17, 129.50, 129.24, 128.80, 128.62, 127.49, 127.35, 127.09, 126.98, 126.87, 126.49, 126.41, 52.08, 49.33, 49.27, 48.35, 43.29, 43.21, 34.83, 33.67. ESI-MS (*m*/*z*) [M + H]^+^: calcd for C_21_H_22_ClN_2_O_3_S 417.1; found 417.0.

#### 2-Chloro-*N*-(furan-2-ylmethyl)-*N*-(4-sulfamoylphenethyl)acetamide (**13**)

Compound **13** was obtained according to the general procedure earlier reported using 4-(2-((furan-2-ylmethyl)amino)ethyl)benzenesulfonamide **5** and 2-chloroacetyl chloride (1.2 equiv). Yield 75%; mp 141–143 °C; silica gel TLC *R*_*f*_ 0.27 (TFA/MeOH/DCM 3/5/92% v/v). δH (400 MHz, DMSO-*d*_6_): 7.74 (t, *J* = 7.6 Hz, 2H, Ar-*H*), 7.67 (s, 0.5H, Ar), 7.61 (s, 0.5H, Ar-*H*), 7.44 (d, *J* = 8.0 Hz, 1H, Ar-*H*), 7.37 (d, *J* = 8.1 Hz, 1H, Ar-*H*), 7.30 (s, 2H, exchange with D_2_O, SO_2_N*H*_2_)_,_ 6.45 (m, 1.5H, Ar-*H*), 6.38 (m, 0.5H, Ar-*H*), 4.57 (s, 2H, C*H*_2_), 4.51 (s, 1H, C*H*_2_), 4.38 (s, 1H, C*H*_2_), 3.48 (dd, *J* = 14.0, 6.3 Hz, 2H, C*H*_2_), 2.90 (t, *J* = 7.7 Hz, 1H, C*H*_2_), 2.75 (m, 1H, C*H*_2_). δC (100 MHz, DMSO-*d*_6_): 166.74, 166.66, 151.48, 151.18, 151.07, 144.24, 144.11, 143.97, 143.86, 143.66, 143.50, 143.36, 143.13, 130.33, 130.05, 126.78, 126.74, 111.62, 111.54, 110.64, 109.83, 109.70, 49.13, 48.21, 45.07, 43.19, 43.09, 42.37, 34.57, 33.47. ESI-MS (*m*/*z*) [M + H]^+^: calcd for C_15_H_18_ClN_2_O_4_S 357.0; found 357.0.

#### 3-Chloro-*N*-(furan-2-ylmethyl)-*N*-(4-sulfamoylphenethyl)propanamide (**14**)

Compound **14** was obtained according to the general procedure earlier reported using 4-(2-((furan-2-ylmethyl)amino)ethyl)benzenesulfonamide **5** and 3-chloropropionyl chloride (1.2 equiv). Yield 71%; mp 113–115 °C; silica gel TLC *R*_*f*_ 0.31 (TFA/MeOH/DCM 3/5/92% v/v). δH (400 MHz, DMSO-*d*_6_): 7.74 (m, 2H, Ar-*H*), 7.64 (s, 0.5H, Ar-*H*), 7.60 (s, 0.5H, Ar-*H*), 7.40 (m, 2H, Ar-*H*), 7.29 (s, 2H, exchange with D_2_O, SO_2_N*H*_2_), 6.42 (m, 1.5H, Ar-*H*), 6.34 (s, 0.5H, Ar-*H*) 4.55 (s, 1H, C*H*_2_), 4.54 (s, 1H, C*H*_2_), 3.81 (t, *J* = 6.5 Hz, 1H, C*H*_2_), 3.76 (t, *J* = 6.4 Hz, 1H, C*H*_2_), 3.48 (m, 2H, C*H*_2_), 2.97 (t, *J* = 6.5 Hz, 1H, C*H*_2_), 2.79 (m, 3H, 2 × C*H*_2_). δC (100 MHz, DMSO-*d*_6_): 170.08, 169.94, 151.94, 151.67, 144.21, 144.03, 143.61, 143.50, 143.33, 143.07, 130.29, 130.04, 126.77, 126.73, 111.54, 111.52, 109.47, 109.28, 48.75, 47.96, 44.98, 41.87, 41.59, 41.43, 36.49, 35.98, 34.73, 33.82. ESI-MS (*m*/*z*) [M + H]^+^: calcd for C_16_H_20_ClN_2_O_4_S 371.1; found 371.1.

#### 2-Chloro-*N*-(2-cyanoethyl)-*N*-(4-sulfamoylphenethyl)acetamide (**15**)

Compound **15** was obtained according to the general procedure earlier reported using 4-(2-((2-cyanoethyl)amino)ethyl)benzenesulfonamide **6** and 2-chloroacetyl chloride (1.2 equiv). Yield 71%; mp 199–201 °C; silica gel TLC *R*_*f*_ 0.04 (TFA/MeOH/DCM 3/5/92% v/v). δH (400 MHz, DMSO-*d*_6_): 7.77 (d, *J* = 7.4 Hz, 2H, Ar-*H*), 7.51 (d, 7.3 Hz, 2H, Ar-*H*), 7.29 (s, 2H, exchange with D_2_O, SO_2_N*H*_2_), 4.46 (s, 1H, C*H*_2_), 4.36 (s, 1H, C*H*_2_), 3.59 (s, 4H, 2 × C*H*_2_), 2.86 (m, 4H, 2 × C*H*_2_). δC (100 MHz, DMSO-*d*_6_): 167.37, 167.13, 144.04, 143.50, 143.24, 130.54, 130.29, 126.85, 124.09, 120.03, 95.68, 49.92, 47.96, 44.16, 43.20, 43.02, 42.82, 34.99, 33.62, 18.01, 16.44. ESI-MS (*m*/*z*) [M + H]^+^: calcd for C_13_H_17_ClN_3_O_3_S 330.0; found 330.0.

#### 2-Chloro-*N*-phenethyl-*N*-(4-sulfamoylphenethyl)acetamide (**16**)

Compound **16** was obtained according to the general procedure earlier reported using 4-(2-(phenethylamino)ethyl)benzenesulfonamide **7** and 2-chloroacetyl chloride (1.2 equiv). Yield 92%; mp 178–180 °C; silica gel TLC *R*_*f*_ 0.29 (TFA/MeOH/DCM 3/5/92% v/v). δH (400 MHz, DMSO-*d*_6_): 7.75 (d, *J* = 7.1 Hz, 2H, Ar-*H*), 7.44 (dd, *J* = 15.3, 8.2 Hz, 2H, Ar-*H*), 7,29 (s, 2H, exchange with D_2_O, SO_2_N*H*_2_, overlap with signal at 7.26)_,_ 7.26 (m, 5H, Ar-*H*), 4.29 (s, 0.9H, C*H*_2_), 4.18 (s, 1.1H, C*H*_2_), 3.47 (m, 4H 0.9H, C*H*_2_), 2.92 (m, 1H, C*H*_2_), 2.85 (t, *J* = 7.5 Hz, 2H, C*H*_2_), 2.77 (m, 1H, C*H*_2_). δC (100 MHz, DMSO-*d*_6_): 167.49, 144.74, 144.06, 143.60, 143.25, 140.38, 139.73, 130.85, 130.66, 130.32, 130.12, 129.92, 129.81, 127.94, 127.67, 127.18, 127.12, 50.73, 50.37, 48.96, 48.41, 43.08, 43.05, 35.57, 35.33, 34.26, 33.98. ESI-MS (*m*/*z*) [M + H]^+^: calcd for C_18_H_22_ClN_2_O_3_S 381.1; found 381.0.

#### 3-Chloro-*N*-phenethyl-*N*-(4-sulfamoylphenethyl)propanamide (**17**)

Compound **17** was obtained according to the general procedure earlier reported using 4-(2-(phenethylamino)ethyl)benzenesulfonamide **7** and 3-chloropropionyl chloride (1.2 equiv). Yield 54%; mp 181–183 °C; silica gel TLC *R*_*f*_ 0.34 (TFA/MeOH/DCM 3/5/92% v/v). δH (400 MHz, DMSO-*d*_6_): 7.74 (d, *J* = 8.1 Hz, 2H, Ar-*H*), 7.43 (dd, *J* = 12.1, 8.2 Hz, 2H, Ar-*H*), 7.30 (s, 2H, exchange with D_2_O, SO_2_N*H*_2_, overlap with signal at 7.24), 7.24 (m, 5H, Ar-*H*), 3.75 (t, *J* = 6.4 Hz, 1H, C*H*_2_), 3.70 (t, *J* = 6.4 Hz, 1H, C*H*_2_), 3.46 (s, 4H, 2 × C*H*_2_), 2.85 (m, 5H, 3 × C*H*_2_), 2.64 (t, *J* = 6.4 Hz, 1H, C*H*_2_). δC (100 MHz, DMSO-*d*_6_): 169.88, 169.79, 144.48, 143.65, 143.32, 143.01, 140.18, 139.57, 130.32, 130.08, 129.88, 129.64, 129.35, 129.28, 127.32, 127.09, 126.66, 49.89, 49.53, 48.28, 47.81, 41.71, 36.00, 35.43, 35.17, 34.28, 33.98. ESI-MS (*m*/*z*) [M + H]^+^: calcd for C_19_H_24_ClN_2_O_3_S 395.1; found 395.1.

### Synthesis of 3-(Phenethylamino)propanenitrile

To a solution of phenethylamine (16,5 mmol, 1.0 equiv) in dry DMF (5 mL), triethylamine (1.2 equiv) and 3-chloropropionitrile (1.1 equiv) were added, and the mixture was stirred at room temperature for 0.5 h. The reaction was quenched by addition of water (20 mL) and extracted with EtOAc (30 mL × 3). The organic layer was collected, washed with brine (40 mL × 3), dried over Na_2_SO_4_, filtered off, and evaporated under *vacuum* to give 3-(phenethylamino)propanenitrile as an orange oil. Yield 92%; silica gel TLC *R*_*f*_ 0.42 (TFA/MeOH/DCM 1.5/1.5/97% v/v). 7.23 (m, 5H, Ar-*H*), 2.85 (m, 6H, 3 × C*H*_2_), 2.57 (t, *J* = 6.6 Hz, 2H, C*H*_2_), 1.89 (bs, 1H, exchange with D_2_O, N*H*). δC (100 MHz, DMSO-*d*_6_): 141.30, 129.64, 129.28, 126.90, 120.99, 51.41, 45.80, 37.04, 18.99. ESI-MS (*m*/*z*) [M + H]^+^: calcd for C_11_H_15_N_2_ 175.1; found 175.0.

### General Synthesis Procedure of Three-Tail Compounds **18–39**

To a solution of chloroalkylamide **8–17** (0.69 mmol, 1.0 equiv) and triethylamine (1.2 equiv) in MeCN dry (5 mL), the proper secondary amine (1.1 equiv) was added, and the mixture was heated at reflux temperature for 4–24 h under stirring. The solvent was evaporated under *vacuum*, and the crude was treated with NaHCO_3_ saturated solution (5 mL) and extracted with EtOAc (10 mL × 3). The organic layer was dried over Na_2_SO_4_, filtered, and evaporated under vacuum. The obtained residue was purified by flash chromatography (1% MeOH in DCM) to give compounds **18–39** as an oil or powder.

#### *N*-Benzyl-2-(diethylamino)-*N*-(4-sulfamoylphenethyl)acetamide (**18**)

Compound **18** was obtained according to the general procedure earlier reported using *N*-benzyl-2-chloro-*N*-(4-sulfamoylphenethyl)acetamide **8** and diethylamine (1.1 equiv) in dry MeCN (5 mL) and stirring for 4 h at reflux temperature. The sticky residue was purified by flash chromatography (1% MeOH in DCM) to give **18** as a powder. Yield 71%; mp 93–95 °C; silica gel TLC *R*_*f*_ 0.12 (TFA/MeOH/DCM 3/5/92% v/v). δH (400 MHz, DMSO-*d*_6_): 7.74 (t, *J* = 7.5 Hz, 2H, Ar-*H*), 7.31 (m, 7H, Ar-*H*), 7.27 (s, 2H, exchange with D_2_O, SO_2_N*H*_2_), 4.70 (s, 0.9H, C*H*_2_), 4.56 (s, 1.1H, C*H*_2_), 3.57 (m, 1H, C*H*_2_), 3.44 (m, 1H, C*H*_2_), 3.16 (s, 1H, C*H*_2_), 3.13 (s, 1H, C*H*_2_), 2.94 (m, 1H, C*H*_2_), 2.78 (m, 1H, C*H*_2_), 2.46 (m, 4H, 2 × C*H*_2_), 0.90 (m, 6H, 2 × C*H*_3_). δC (100 MHz, DMSO-*d*_6_): 170.22, 143.34, 143.10, 142.34, 142.13, 138.18, 137.82, 129.25, 129.09, 128.69, 128.42, 127.54, 127.19, 127.00, 126.77, 125.80, 125.72, 56.33, 56.25, 50.03, 47.71, 47.15, 46.76, 46.72, 46.42, 33.89, 32.72, 11.38. ESI-HRMS (*m*/*z*) [M + H]^+^: calcd for C_21_H_30_N_3_O_3_S 404.2007; found 404.2012.

#### *N*-Benzyl-2-(benzyl(ethyl)amino)-*N*-(4-sulfamoylphenethyl)acetamide (**19**)

Compound **19** was obtained according to the general procedure earlier reported using *N*-benzyl-2-chloro-*N*-(4-sulfamoylphenethyl)acetamide **8** and N-ethylbenzylamine (1.1 equiv) in dry MeCN (5 mL) and stirring for 16 h at reflux temperature. The sticky residue was purified by flash chromatography (1% MeOH in DCM) to give **19** as an oil. Yield 65%; silica gel TLC *R*_*f*_ 0.27 (TFA/MeOH/DCM 3/5/92% v/v). δH (400 MHz, DMSO-*d*_6_): 7.75 (t, *J* = 8.5 Hz, 2H, Ar-*H*), 7.37 (m, 11H, Ar-*H*), 7.28 (s, 2H, exchange with D_2_O, SO_2_N*H*_2_, overlap with signal at 7.37), 7.13 (d, *J* = 7.2 Hz, 1H, Ar-*H*), 4.62 (s, 1H, C*H*_2_), 4.60 (s, 1H, C*H*_2_), 3.64 (s, 1H, C*H*_2_), 3.60 (s, 1H, C*H*_2_), 3.55 (m, 1H, C*H*_2_), 3.45 (m, 1H, C*H*_2_), 3.25 (s, 1H, C*H*_2_), 3.15 (s, 1H, C*H*_2_), 2.84 (m, 2H, C*H*_2_), 247 (m, 2H, C*H*_2_), 0.98 (m, 3H, C*H*_3_). δC (100 MHz, DMSO-*d*_6_): 171.12, 144.35, 143.83, 143.39, 143.22, 139.72, 139.03, 138.56, 130.40, 130.26, 130.12, 129.67, 129.42, 129.25, 129.22, 128.62, 128.20, 128.09, 127.69, 126.80, 126.78, 58.40, 56.64, 55.97, 51.10, 48.37, 48.12, 47.70, 34.76, 33.82, 16.77, 13.76, 12.53. ESI-HRMS (*m*/*z*) [M + H]^+^: calcd for C_26_H_32_N_3_O_3_S 466.2164; found 466.2169.

#### *N*-Benzyl-2-(dibenzylamino)-*N*-(4-sulfamoylphenethyl)acetamide (**20**)

Compound **20** was obtained according to the general procedure earlier reported using *N*-benzyl-2-chloro-*N*-(4-sulfamoylphenethyl)acetamide **8** and dibenzylamine (1.1 equiv) in dry MeCN (5 mL) and stirring for 15 h at reflux temperature. The sticky residue was purified by flash chromatography (MeOH 1%/DCM) to give **20** as a white powder. Yield 68%; mp 98–100 °C; silica gel TLC *R*_*f*_ 0.38 (TFA/MeOH/DCM 3/5/92% v/v). δH (400 MHz, DMSO-*d*_6_): 7.67 (t, *J* = 8.1 Hz, 2H, Ar-*H*), 7.28 (m, 15H, Ar-*H*), 7.24 (s, 2H, exchange with D_2_O, SO_2_N*H*_2_, overlap with signal at 7.28), 6.94 (m, 2H, Ar-*H*), 4.54 (s, 1.1H, C*H*_2_), 4.43 (s, 0.9H, C*H*_2_), 3.68 (s, 2.3H, 2 × C*H*_2_), 3.59 (s, 1.7H, 2 × C*H*_2_), 3.41 (m, 2H, C*H*_2_), 3.23 (s, 1H, C*H*_2_), 3.08 (s, 1H, C*H*_2_), 2.79 (t, *J* = 7.3 Hz, 0.9H, C*H*_2_), 2.61 (t, *J* = 6.8 Hz, 1.1H, C*H*_2_). δC (100 MHz, DMSO-*d*_6_): 170.96, 170.84, 144.38, 143.48, 143.28, 143.16, 139.69, 139.61, 139.04, 138.39, 130.13, 129.98, 129.91, 129.57, 129.44, 129.36, 129.29, 128.64, 128.22, 128.10, 127.45, 126.79, 126.71, 58.48, 58.42, 55.73, 55.42, 50.99, 48.13, 47.99, 34.58, 33.91. ESI-HRMS (*m*/*z*) [M + H]^+^: calcd for C_31_H_34_N_3_O_3_S 528.2321; found 466. 528.2317.

#### *N*-Benzyl-2-(dipentylamino)-*N*-(4-sulfamoylphenethyl)acetamide (**21**)

Compound **21** was obtained according to the general procedure earlier reported using *N*-benzyl-2-chloro-*N*-(4-sulfamoylphenethyl)acetamide **8** and dipentylamine (1.1 equiv) in dry MeCN (5 mL) and stirring for 16 h at reflux temperature. The sticky residue was purified by flash chromatography (1% MeOH in DCM) to give **21** as an oil. Yield 70%; silica gel TLC *R*_*f*_ 0.14 (TFA/MeOH/DCM 3/5/92% v/v). δH (400 MHz, DMSO-*d*_6_): 7.73 (t, *J* = 7.8 Hz, 2H, Ar-*H*), 7.34 (m, 7H, Ar-*H*), 7.28 (s, 2H, exchange with D_2_O, SO_2_N*H*_2_, overlap with signal at 7.34), 4.73 (s, 0.7H, C*H*_2_), 4.58 (s, 1.3H, C*H*_2_), 3.61 (m, 0.9H, C*H*_2_), 3.46 (m, 1.1H, C*H*_2_), 3.15 (m, 2H, C*H*_2_), 2.95 (m, 1.1H, C*H*_2_), 2.81(m, 0.9H, C*H*_2_), 2.43 (m, 4H, 2x C*H*_2_), 1.21 (m, 12H, 6 × C*H*_2_), 0.81 (m, 6H, 2 × C*H*_3_). δC (100 MHz, DMSO-*d*_6_): 167.34, 144.32, 144.01, 143.73, 143.45, 143.22, 138.70, 130.29, 130.08, 129.73, 129.40, 128.79, 128.62, 128.22, 128.14, 127.68, 126.82, 54.91, 54.85, 51.87, 51.13, 49.31, 48.49, 48.24, 47.90, 43.11, 34.74, 33.88, 30.18, 22.95, 22.75, 16.34, 15.05, 14.73. ESI-HRMS (*m*/*z*) [M + H]^+^: calcd for C_27_H_42_N_3_O_3_S 488.2947; found 488.2942.

#### *N*-Benzyl-2-(dihexylamino)-*N*-(4-sulfamoylphenethyl)acetamide (**22**)

Compound **22** was obtained according to the general procedure earlier reported using *N*-benzyl-2-chloro-*N*-(4-sulfamoylphenethyl)acetamide **8** and dihexylamine (1.1 equiv) in dry MeCN (5 mL) and stirring for 16 h at reflux temperature. The sticky residue was purified by flash chromatography (1% MeOH in DCM) to give **22** as an oil. Yield 72%; silica gel TLC *R*_*f*_ 0.34 (TFA/MeOH/DCM 3/5/92% v/v). δH (400 MHz, DMSO-*d*_6_): 7.73 (t, *J* = 7.5 Hz, 2H, Ar-*H*), 7.33 (m, 7H, Ar-*H*), 7.26 (s, 2H, exchange with D_2_O, SO_2_N*H*_2_, overlap with signal at 7.33), 4.73 (s, 0.9H, C*H*_2_), 4.56 (s, 1.1H, C*H*_2_), 3.62 (m, 1H, C*H*_2_), 3.42 (m, 1H, C*H*_2_), 3.14 (m, 1H, C*H*_2_), 2.94 (m, 1.1H, C*H*_2_), 2.82 (m, 0.9H, C*H*_2_), 2.41 (m, 4H, 2x C*H*_2_), 1.26 (m, 16H, 8 × C*H*_2_), 0.83 (m, 6H, 2 × C*H*_3_). δC (100 MHz, DMSO-*d*_6_): 170.65, 170.49, 143.73, 143.43, 142.85, 142.63, 138.61, 138.23, 129.62, 129.41, 129.07, 128.74, 128.18, 127.58, 127.46, 127.04, 126.21, 65.33, 58.19, 57.91, 54.32, 54.21, 50.55, 47.89, 47.69, 47.35, 47.31, 34.32, 33.32, 31.63, 31.17, 27.03, 26.99, 26.80, 26.75, 26.11, 26.00, 22.52, 22.32, 15.65, 14.32, 14.27. ESI-HRMS (*m*/*z*) [M + H]^+^: calcd for C_29_H_46_N_3_O_3_S 516.3260; found 516.3264.

#### *N*-Benzyl-2-(dioctylamino)-*N*-(4-sulfamoylphenethyl)acetamide (**23**)

Compound **23** was obtained according to the general procedure earlier reported using *N*-benzyl-2-chloro-*N*-(4-sulfamoyl-phenethyl)acetamide **8** and dioctylamine (1.1 equiv) in dry MeCN (5 mL) and stirring for 16 h at reflux temperature. The sticky residue was purified by flash chromatography (MeOH 1%/DCM) to give **23** as a powder. Yield 67%; mp 62–64 °C; silica gel TLC *R*_*f*_ 0.16 (TFA/MeOH/DCM 3/5/92% v/v). δH (400 MHz, DMSO-*d*_6_): 7.72 (t, *J* = 7.5 Hz, 2H, Ar-*H*), 7.32 (m, 7H, Ar-*H*), 7.27 (s, 2H, exchange with D_2_O, SO_2_N*H*_2_, overlap with signal at 7.32), 4.74 (s, 0.9H, C*H*_2_), 4.56 (s, 1.1H, C*H*_2_), 3.61 (m, 1H, C*H*_2_), 3.38 (m, 1H, C*H*_2_), 3.14 (m, 1H, C*H*_2_), 2.95 (m, 1.1H, C*H*_2_), 2.81 (m, 0.9H, C*H*_2_), 2.37 (m, 4H, 2x C*H*_2_), 1.28 (m, 24H, 12 × C*H*_2_), 0.83 (m, *J* = 6.2 Hz, 6H, 2 × C*H*_3_). δC (100 MHz, DMSO-*d*_6_): 170.23, 170.10, 143.27, 143.01, 142.38, 142.17, 138.18, 137.82, 129.20, 128.99, 128.63, 128.31, 127.74, 127.14, 127.01, 126.60, 125.76, 64.93, 57.81, 57.59, 53.78, 53.70, 50.04, 47.41, 47.14, 46.88, 33.84, 32.86, 31.27, 31.23, 28.91, 28.70, 28.68, 26.93, 26.88, 26.42, 26.35, 22.09, 15.18, 13.94. ESI-HRMS (*m*/*z*) [M + H]^+^: calcd for C_33_H_54_N_3_O_3_S 572.3886; found 572.3881.

#### *N*-Benzyl-3-(diethylamino)-*N*-(4-sulfamoylphenethyl)propanamide (**24**)

Compound **24** was obtained according to the general procedure earlier reported using *N*-benzyl-3-chloro-*N*-(4-sulfamoylphenethyl)propanamide **9** and diethylamine (1.1 equiv) in dry MeCN (5 mL) and stirring for 16 h at reflux temperature. The sticky residue was purified by flash chromatography (1% MeOH in DCM) to give **24** as an oil. Yield 72%; silica gel TLC *R*_*f*_ 0.04 (TFA/MeOH/DCM 3/5/92% v/v). δH (400 MHz, DMSO-*d*_6_): 7.74 (t, *J* = 7.8 Hz, 2H, Ar-*H*), 7.33 (m, 7H, Ar-*H*), 7.30 (s, 2H, exchange with D_2_O, SO_2_N*H*_2_, overlap with signal at 7.33), 4.58 (s, 0.9H, C*H*_2_), 4.56 (s, 1.1H, C*H*_2_), 3.49 (t, *J* = 7.4 Hz, 2H, C*H*_2_), 3,38 (m, 2H, C*H*_2_), 2.92 (t, *J* = 7.3 Hz, 1,1H, C*H*_2_), 2.85 (m, 0.9H, C*H*_2_), 2.72 (m, 2H, C*H*_2_), 2.45 (m, 4H, 2 × C*H*_2_), 0.96 (m, 6H, 2 × C*H*_3_). δC (100 MHz, DMSO-*d*_6_): 172.42, 172.16, 144.45, 143.90, 143.42, 143.17, 139.17, 138.88, 130.37, 130.15, 129.76, 129.43, 128.68, 128.28, 128.02, 127.57, 126.84, 126.80, 55.97, 51.78, 49.67, 49.40, 49.00, 48.40, 48.29, 47.39, 47.35, 34.96, 34.30, 34.11, 30.56, 12.48. ESI-HRMS (*m*/*z*) [M + H]^+^: calcd for C_22_H_32_N_3_O_3_S 418.2164; found 418.2170.

#### *N*-Benzyl-3-(benzyl(ethyl)amino)-*N*-(4-sulfamoylphenethyl)propanamide (**25**)

Compound **25** was obtained according to the general procedure earlier reported using *N*-benzyl-3-chloro-*N*-(4-sulfamoylphenethyl)propanamide **9** and N-ethylbenzylamine (1.1 equiv) in dry MeCN (5 mL) and stirring for 20 h at reflux temperature. The sticky residue was purified by flash chromatography (1% MeOH in DCM) to give **25** as a powder. Yield 69%; mp 77–79 °C; silica gel TLC *R*_*f*_ 0.12 (TFA/MeOH/DCM 3/5/92% v/v). δH (400 MHz, DMSO-*d*_6_): 7.73 (t, *J* = 7.8 Hz, 2H, Ar-*H*), 7.35 (m, 12H, Ar-*H*), 7.33 (s, 2H, exchange with D_2_O, SO_2_N*H*_2_, overlap with signal at 7.35), 4.53 (s, 2H, C*H*_2_), 4.11 (s, 1H, C*H*_2_), 3.53 (m, 3H, 2 × C*H*_2_), 2.86 (m, 3H, 2 × C*H*_2_), 2.67 (m, 1H, C*H*_2_), 2.39 (m, 2H, C*H*_2_), 1.21 (t, *J* = 7.2 Hz, 2H, C*H*_2_), 0.95 (m, 3H, 1 × C*H*_3_). δC (100 MHz, DMSO-*d*_6_): 172.27, 171.59, 144.43, 143.82, 143.43, 143.17, 139.16, 138.99, 138.81, 133.37, 130.99, 130.38, 130.12, 129.86, 129.76, 129.68, 129.45, 129.25, 128.71, 128.31, 128.05, 127.63, 126.83, 126.82, 58.05, 51.74, 50.52, 48.99, 48.42, 48.24, 47.94, 47.76, 42.70, 34.95, 34.09, 11.96. ESI-HRMS (*m*/*z*) [M + H]^+^: calcd for C_27_H_34_N_3_O_3_S 480.2321; found 480.2315.

#### *N*-Benzyl-3-(dibenzylamino)-*N*-(4-sulfamoylphenethyl)propanamide (**26**)

Compound **26** was obtained according to the general procedure earlier reported using *N*-benzyl-3-chloro-*N*-(4-sulfamoylphenethyl)propanamide **9** and dibenzylamine (1.1 equiv) in dry MeCN (5 mL) and stirring for 22 h at reflux temperature. The sticky residue was purified by flash chromatography (1% MeOH in DCM) to give **26** as an oil. Yield 64%; silica gel TLC *R*_*f*_ 0.32 (TFA/MeOH/DCM 3/5/92% v/v). δH (400 MHz, DMSO-*d*_6_): 7.67 (m, 2H, Ar-*H*), 7.30 (s, 2H, exchange with D_2_O, SO_2_N*H*_2_, overlap with signal at 7.29), 7.29 (m, 15H, Ar-*H*), 6.93 (m, 2H, Ar-*H*), 4.53 (s, 1.1H, C*H*_2_), 4.41 (s, 0.9H, C*H*_2_), 3.67 (s, 0.9H, C*H*_2_), 3.59 (s, 1.1H, C*H*_2_), 3.45 (m, 6H, 3 × C*H*_2_), 3.23 (s, 0.9H, C*H*_2_), 3.08 (s, 1.1H, C*H*_2_), 2.77 (m, 1H, C*H*_2_), 2.60 (t, *J* = 7.1 Hz, 1H, C*H*_2_). δC (100 MHz, DMSO-*d*_6_): 170.99, 170.87, 144.38, 143.48, 143.28, 143.17, 139.68, 139.61, 139.02, 138.36, 130.53, 130.21, 130.11, 129.97, 129.90, 129.55, 129.42, 129.34, 129.28, 128.64, 128.20, 128.09, 127.46, 126.79, 126.71, 58.50, 58.45, 55.73, 55.45, 51.03, 49.65, 48.15, 48.05, 47.94, 34.60, 33.91, 22.08. ESI-HRMS (*m*/*z*) [M + H]^+^: calcd for C_32_H_36_N_3_O_3_S 542.2477; found 542.2473.

#### *N*-Benzyl-3-(dipentylamino)-*N*-(4-sulfamoylphenethyl)propanamide (**27**)

Compound **27** was obtained according to the general procedure earlier reported using *N*-benzyl-3-chloro-*N*-(4-sulfamoylphenethyl)propanamide **9** and dipentylamine (1.1 equiv) in dry MeCN (5 mL) and stirring for 16 h at reflux temperature. The sticky residue was purified by flash chromatography (1% MeOH in DCM) to give **27** as an oil. Yield 73%; silica gel TLC *R*_*f*_ 0.16 (TFA/MeOH/DCM 3/5/92% v/v). δH (400 MHz, DMSO-*d*_6_): 7.72 (m, 2H, Ar-*H*), 7.29 (m, 7H, Ar-*H*), 7.27 (s, 2H, exchange with D_2_O, SO_2_N*H*_2_, overlap with signal at 7.29), 4.57 (s, 1.1H, C*H*_2_), 4.54 (s, 0.9H, C*H*_2_), 3.46 (m, 2H, C*H*_2_), 2.89 (m, 1H, C*H*_2_), 2.82 (m, 1H, C*H*_2_), 2.63 (s, 4H, 2 × C*H*_2_), 2.32 (m, 4H, 2 × C*H*_2_), 1.27 (m, 12H, 6 × C*H*_2_), 0.84 (m, 6H, 2 × C*H*_3_). δC (100 MHz, DMSO-*d*_6_): 172.97, 172.71, 144.46, 143.82, 143.41, 143.13, 139.21, 138.96, 130.27, 130.13, 129.71, 129.39, 128.66, 128.23, 128.00, 127.39, 126.83, 126.80, 65.98, 54.42, 54.36, 51.91, 51.00, 50.60, 49.18, 49.09, 48.54, 48.42, 35.12, 34.17, 32.30, 32.26, 32.04, 31.23, 30.93, 28.41, 27.78, 27.68, 27.62, 27.57, 27.13, 23.18, 23.04, 16.21, 14.97, 14.94. ESI-HRMS (*m*/*z*) [M + H]^+^: calcd for C_28_H_44_N_3_O_3_S 502.3103; found 502.3098.

#### *N*-Benzyl-3-(dihexylamino)-*N*-(4-sulfamoylphenethyl)propanamide (**28**)

Compound **28** was obtained according to the general procedure earlier reported using *N*-benzyl-3-chloro-*N*-(4-sulfamoylphenethyl)propanamide **9** and dipentylamine (1.1 equiv) in dry MeCN (5 mL) and stirring for 16 h at reflux temperature. The sticky residue was purified by flash chromatography (1% MeOH in DCM) to give **28** as an oil. Yield 74%; silica gel TLC *R*_*f*_ 0.20 (TFA/MeOH/DCM 3/5/92% v/v). δH (400 MHz, DMSO-*d*_6_): 7.73 (m, 2H, Ar-*H*), 7.32 (m, 7H, Ar-*H*), 7.30 (s, 2H, exchange with D_2_O, SO_2_N*H*_2_, overlap with signal at 7.32), 4.60 (s, 0.9H, C*H*_2_), 4.56 (s, 1.1H, C*H*_2_), 3.47 (m, 2H, C*H*_2_), 3.17 (m, 2H, C*H*_2_), 2.85 (m, 8H, 4 × C*H*_2_), 1.55 (m, 4H, 2 × C*H*_2_), 1.27 (m, 12H, 6 × C*H*_2_), 0.87 (m, 6H, 2 × C*H*_3_). δC (100 MHz, DMSO-*d*_6_): 172.90, 172.65, 144.47, 143.82, 143.44, 143.16, 139.27, 139.03, 130.27, 130.12, 129.71, 129.39, 128.66, 128.21, 127.99, 127.40, 126.83, 126.81, 54.44, 54.37, 54.09, 51.89, 51.02, 50.62, 49.73, 49.16, 48.53, 48.43, 36.03, 35.15, 34.20, 32.32, 32.27, 32.19, 31.69, 31.26, 30.95, 30.24, 29.40, 27.84, 27.72, 27.63, 27.59, 27.36, 23.20, 23.11, 14.99. ESI-HRMS (*m*/*z*) [M + H]^+^: calcd for C_30_H_48_N_3_O_3_S 530.3416; found 530.3421.

#### *N*-Benzyl-3-(dioctylamino)-*N*-(4-sulfamoylphenethyl)propanamide (**29**)

Compound **29** was obtained according to the general procedure earlier reported using *N*-benzyl-3-chloro-*N*-(4-sulfamoylphenethyl)propanamide **9** and dioctylamine (1.1 equiv) in dry MeCN (5 mL) and stirring for 16 h at reflux temperature. The sticky residue was purified by flash chromatography (1% MeOH in DCM) to give **29** as an oil. Yield 73%; silica gel TLC *R*_*f*_ 0.22 (TFA/MeOH/DCM 3/5/92% v/v). δH (400 MHz, DMSO-*d*_6_): 7.73 (m, 2H, Ar-*H*), 7.31 (m, 7H, Ar-*H*), 7.30 (s, 2H, exchange with D_2_O, SO_2_N*H*_2_, overlap with signal at 7.31), 4.60 (s, 0.9H, C*H*_2_), 4.56 (s, 1.1H, C*H*_2_), 3.47 (m, 2H, C*H*_2_), 2.93 (m, 4H, 2 × C*H*_2_), 2.82 (m, 4H, 2 × C*H*_2_), 2.66 (m, 1H, C*H*_2_), 2.57 (m, 1H, C*H*_2_), 1.58 (m, 4H, 2 × C*H*_2_), 1.27 (m, 20H, 5 × C*H*_2_), 0.85 (m, 6H, 2 × C*H*_3_). δC (100 MHz, DMSO-*d*_6_): 171.86, 171.61, 143.40, 142.75, 142.41, 142.12, 138.19, 137.95, 129.19, 129.05, 128.64, 128.33, 127.62, 127.16, 126.92, 126.37, 125.79, 125.75, 53.36, 53.31, 50.87, 49.99, 49.63, 49.15, 48.11, 47.47, 47.41, 34.12, 33.15, 31.27, 30.28, 29.91, 29.12, 28.96, 28.95, 28.92, 28.74, 28.73, 28.70, 26.88, 26.85, 26.81, 26.80, 26.72, 22.09, 13.94, 13.92. ESI-HRMS (*m*/*z*) [M + H]^+^: calcd for C_34_H_56_N_3_O_3_S 586.4042; found 586.4036.

#### 3-(Dihexylamino)-*N*-phenethyl-*N*-(4-sulfamoylphenethyl)propanamide (**30**)

Compound **30** was obtained according to the general procedure earlier reported using 3-chloro-*N*-phenethyl-*N*-(4-sulfamoylphenethyl)propanamide **17** and dihexylamine (1.1 equiv) in dry MeCN (5 mL) and stirring for 16 h at reflux temperature. The sticky residue was purified by flash chromatography (1% MeOH in DCM) to give **30** as an oil. Yield 68%; silica gel TLC *R*_*f*_ 0.21 (TFA/MeOH/DCM 3/5/92% v/v). δH (400 MHz, DMSO-*d*_6_): 7.75 (m, 2H, Ar-*H*), 7.35 (m, 7H, Ar-*H*), 7.30 (s, 2H, exchange with D_2_O, SO_2_N*H*_2_, overlap with signal at 7.35), 4.11 (m, 2H, C*H*_2_), 3.49 (m, 2H, C*H*_2_), 3.16 (m, 4H, 2 × C*H*_2_), 2.90 (m, 4H, 2 × C*H*_2_), 2.08 (m, 4H, 2 × C*H*_2_), 1.60 (m, 4H, 2 × C*H*_2_), 1.29 (m, 12H, 6 × C*H*_2_), 0.87 (m, 6H, 2 × C*H*_3_). δC (100 MHz, DMSO-*d*_6_): 170.21, 170.08, 144.37, 143.78, 143.42, 143.22, 140.21, 139.72, 130.56, 130.50, 130.19, 130.05, 129.75, 129.66, 129.44, 127.45, 127.12, 126.82, 65.98, 55.92, 53.01, 49.69, 49.64, 49.07, 48.24, 47.79, 35.36, 35.12, 34.47, 34.14, 31.78, 26.80, 26.39, 23.90, 22.98, 16.22, 14.95, 14.90. ESI-HRMS (*m*/*z*) [M + H]^+^: calcd for C_31_H_50_N_3_O_3_S 544.3573; found 544.3578.

#### 3-(Dihexylamino)-*N*-(furan-2-ylmethyl)-*N*-(4-sulfamoylphenethyl)propenamide (**31**)

Compound **31** was obtained according to the general procedure earlier reported using 3-chloro-*N*-(furan-2-ylmethyl)-*N*-(4-sulfamoylphenethyl)propenamide **14** and dihexylamine (1.1 equiv) in dry MeCN (5 mL) and stirring for 16 h at reflux temperature. The sticky residue was purified by flash chromatography (1% MeOH in DCM) and to give **31** as a powder. Yield 68%; mp 118–120 °C; silica gel TLC *R*_*f*_ 0.26 (TFA/MeOH/DCM 3/5/92% v/v). δH (400 MHz, DMSO-*d*_6_): 7.73 (d, *J* = 8.3 Hz, 2H, Ar-*H*), 7.60 (m, 1H, Ar-*H*), 7.36 (d, *J* = 8.3 Hz, 2H, Ar-*H*), 7.30 (s, 2H, exchange with D_2_O, SO_2_N*H*_2_), 6.40 (m, 2H, Ar-*H*), 4.57 (s, 0.9H, C*H*_2_), 4.54 (s, 1.1H, C*H*_2_), 3.47 (m, 2H, C*H*_2_), 3.16 (m, 2H, C*H*_2_), 2.82 (m, 8H, 4 × C*H*_2_), 1.56 (m, 4H, 2 × C*H*_2_), 1.27 (m, 12H, 6 × C*H*_2_), 0.85 (m, 6H, 2 × C*H*_3_). δC (100 MHz, DMSO-*d*_6_): 152.21, 151.98, 144.38, 144.00, 143.75, 143.51, 143.45, 143.16, 130.30, 130.09, 126.84, 111.62, 111.59, 109.50, 109.25, 54.06, 50.19, 49.65, 49.14, 48.04, 47.80, 45.36, 41.86, 34.96, 33.98, 32.17, 31.91, 31.77, 27.41, 26.71, 26.48, 23.15, 22.94, 14.97, 14.91. ESI-HRMS (*m*/*z*) [M + H]^+^: calcd for C_28_H_46_N_3_O_4_S 520.3209; found 520.3215.

#### 2-(Dihexylamino)-*N*-(naphthalen-2-ylmethyl)-*N*-(4-sulfamoylphenethyl)acetamide (**32**)

Compound **32** was obtained according to the general procedure earlier reported using 2-chloro-*N*-(naphthalen-2-ylmethyl)-*N*-(4-sulfamoylphenethyl)acetamide **12** and dihexylamine (1.1 equiv) in dry MeCN (5 mL) and stirring for 16 h at reflux temperature. The sticky residue was purified by flash chromatography (1% MeOH in DCM) to give **32** as an oil. Yield 61%; silica gel TLC *R*_*f*_ 0.16 (TFA/MeOH/DCM 3/5/92% v/v). δH (400 MHz, DMSO-*d*_6_): 7.89 (m, 3H, Ar-*H*), 7.71 (m, 3H, Ar-*H*), 7.41 (m, 7H, Ar-*H*), 7.29 (s, 2H, exchange with D_2_O, SO_2_N*H*_2_, overlap with signal at 7.41), 4.92 (s, 0.9H, C*H*_2_), 4.73 (s, 1.1H, C*H*_2_), 3.68 (m, 1H, C*H*_2_), 3.51 (m, 1H, C*H*_2_), 3.23 (m, 1.1H, C*H*_2_), 3.14 (m, 0.9H, C*H*_2_), 2.99 (m, 1H, C*H*_2_), 2.85 (m, 1H, C*H*_2_), 2.37 (m, 4H, 2 × C*H*_2_), 1.29 (m, 4H, 2 × C*H*_2_), 1.14 (m, 12H, 6 × C*H*_2_), 0.78 (t, *J* = 6.4 Hz, 6H, 2 × C*H*_3_). δC (100 MHz, DMSO-*d*_6_): 170.44, 170.23, 143.32, 143.08, 142.36, 142.15, 135.81, 135.53, 133.05, 132.89, 132.22, 129.24, 129.04, 128.27, 127.99, 127.58, 127.46, 126.37, 126.34, 126.24, 126.20, 126.16, 125.85, 125.76, 125.02, 124.61, 64.93, 57.91, 57.48, 53.86, 53.76, 50.17, 47.61, 47.38, 47.08, 34.03, 32.82, 31.17, 26.61, 26.56, 26.38, 22.06, 15.18, 13.89, 13.86. ESI-HRMS (*m*/*z*) [M + H]^+^: calcd for C_33_H_48_N_3_O_3_S 566.3416; found 566.3410.

#### *N*-(2-Cyanoethyl)-2-(dihexylamino)-*N*-(4-sulfamoylphenethyl)acetamide (**33**)

Compound **33** was obtained according to the general procedure earlier reported using 2-chloro-*N*-(2-cyanoethyl)-*N*-(4-sulfamoylphenethyl)acetamide **15** and dihexylamine (1.1 equiv) in dry MeCN (5 mL) and stirring for 16 h at reflux temperature. The sticky residue was purified by flash chromatography (1% MeOH in DCM) to give **33** as an oil. Yield 66%; silica gel TLC *R*_*f*_ 0.08 (TFA/MeOH/DCM 3/5/92% v/v). δH (400 MHz, DMSO-*d*_6_): 7.75 (d, *J* = 7.9 Hz, 2H, Ar-*H*), 7.44 (d, *J* = 8.0 Hz, 2H, Ar-*H*), 7.29 (s, 2H, exchange with D_2_O, SO_2_N*H*_2_), 3.74 (m, 2H, C*H*_2_), 3.50 (m, 2H, C*H*_2_), 3.27 (s, 1H, C*H*_2_), 3.07 (s, 1H, C*H*_2_), 2.95 (m, 1H, C*H*_2_), 2.85 (m, 2H, C*H*_2_), 2.71 (m, 1H, C*H*_2_), 2.37 (m, 4H, 2 × C*H*_2_), 1.36 (m, 4H, 2 × C*H*_2_), 1.22 (m, 12H, 6 × C*H*_2_), 0.84 (m, 6H, 2 × C*H*_3_). δC (100 MHz, DMSO-*d*_6_): 171.58, 171.22, 144.28, 143.93, 143.50, 143.44, 143.24, 130.36, 130.19, 126.81, 126.78, 120.18, 59.14, 58.70, 54.81, 49.27, 47.39, 43.72, 42.10, 35.16, 34.82, 33.81, 32.25, 32.23, 27.66, 27.61, 27.32, 27.23, 23.15, 23.13, 17.86, 16.46, 14.99, 14.96. ESI-HRMS (*m*/*z*) [M + H]^+^: calcd for C_25_H_43_N_4_O_3_S 479.3055; found 479.3049.

#### 2-((2-Cyanoethyl)(phenethyl)amino)-*N*-phenethyl-*N*-(4-sulfamoylphenethyl)acetamide (**34**)

Compound **34** was obtained according to the general procedure earlier reported using 2-chloro-*N*-phenethyl-*N*-(4-sulfamoylphenethyl)acetamide **16** and 3-(phenethylamino)propanenitrile (1.1 equiv) in MeCN dry (5 mL) and stirring for 18 h at reflux temperature. The sticky residue was purified by flash chromatography (1% MeOH in DCM) to give **34** as a powder. Yield 68%; mp 118–120 °C; silica gel TLC *R*_*f*_ 0.26 (TFA/MeOH/DCM 3/5/92% v/v). δH (400 MHz, DMSO-*d*_6_): 7.74 (d, *J* = 6.8 Hz, 2H, Ar-*H*), 7.42 (d, *J* = 8.1 Hz, 2H, Ar-*H*), 7.29 (s, 2H, exchange with D_2_O, SO_2_N*H*_2_, overlap with signal at 7.23), 7.23 (m, 10H, Ar-*H*), 3.46 (m, 4H, 2 × C*H*_2_), 3.28 (s, 0.9H, C*H*_2_), 3.16 (s, 1.1H, C*H*_2_), 2.71 (m, 12H, 6 × C*H*_2_). δC (100 MHz, DMSO-*d*_6_): 170.49, 170.33, 144.45, 143.98, 143.23, 143.02, 141.07, 140.19, 139.84, 135.53, 130.30, 130.07, 129.95, 129.61, 129.60, 129.57, 129.52, 129.30, 129.25, 129.13, 127.27, 127.05, 126.77, 126.63, 121.05, 56.31, 56.13, 56.06, 55.91, 55.83, 51.24, 50.06, 49.20, 48.93, 47.63, 47.22, 45.60, 36.82, 35.15, 34.98, 34.36, 34.15, 34.10, 33.86, 18.75, 16.72, 16.56. ESI-HRMS (*m*/*z*) [M + H]^+^: calcd for C_29_H_35_N_4_O_3_S 519.2430; found 519.2434.

#### 2-((2-Cyanoethyl)(phenethyl)amino)-*N*-(furan-2-ylmethyl)-*N*-(4-sulfamoylphenethyl)acetamide (**35**)

Compound **35** was obtained according to the general procedure earlier reported using 2-chloro-*N*-(furan-2-ylmethyl)-*N*-(4-sulfamoylphenethyl)acetamide **13** and 3-(phenethylamino)propanenitrile (1.1 equiv) in dry MeCN (5 mL) and stirring for 16 h at reflux temperature. The sticky residue was purified by flash chromatography (1% MeOH in DCM) to give **35** as an oil. Yield 70%; silica gel TLC *R*_*f*_ 0.20 (TFA/MeOH/DCM 3/5/92% v/v). δH (400 MHz, DMSO-*d*_6_): 7.69 (m, 3H, Ar-*H*), 7.27 (m, 7H, Ar-*H*), 7.22 (s, 2H, exchange with D_2_O, SO_2_N*H*_2_, overlap with signal at 7.27), 6.39 (m, 2H, Ar-*H*), 3.57 (s, 1H, C*H*_2_), 3.49 (m, 2H, C*H*_2_), 3.45 (s, 1H, C*H*_2_), 2.72 (m, 12H, 6 × C*H*_2_). δC (100 MHz, DMSO-*d*_6_): 169.56, 169.50, 151.07, 150.90, 143.25, 143.03, 142.96, 142.49, 142.34, 142.12, 140.12, 140.08, 129.33, 129.06, 128.69, 128.67, 128.61, 128.26, 128.22, 125.87, 125.84, 125.77, 120.14, 110.61, 110.54, 108.49, 108.40, 55.52, 55.36, 55.16, 54.96, 49.26, 49.16, 46.74, 44.63, 43.49, 33.64, 33.25, 33.09, 32.76, 30.71, 15.74, 15.63. *m*/*z* (ESI positive) 495.3 [M + H]^+^.

#### 2-((2-Cyanoethyl)(phenethyl)amino)-*N*-(4-fluorobenzyl)-*N*-(4-sulfamoylphenethyl)acetamide (**36**)

Compound **36** was obtained according to the general procedure earlier reported using 2-chloro-*N*-(4-fluorobenzyl)-*N*-(4-sulfamoylphenethyl)acetamide **11** and 3-(phenethylamino)propanenitrile (1.1 equiv) in dry MeCN (5 mL) and stirring for 17 h at reflux temperature. The sticky residue was purified by flash chromatography (1% MeOH in DCM) to give **36** as an oil. Yield 73%; silica gel TLC *R*_*f*_ 0.24 (TFA/MeOH/DCM 3/5/92% v/v). δH (400 MHz, DMSO-*d*_6_): 7.73 (t, *J* = 9.7 Hz, 2H, Ar-*H*), 7.30 (s, 2H, exchange with D_2_O, SO_2_N*H*_2_, overlap with signal at 7.29), 7.29 (m, 11H, Ar-*H*), 4.52 (s, 1.1H, C*H*_2_), 4.41 (s, 0.9H, C*H*_2_), 3.43 (m, 2H, C*H*_2_), 2.74 (m, 12H, 6 × C*H*_2_). δF (376 MHz, DMSO-*d*_6_): -115.54, −115.71. δC (100 MHz, DMSO-*d*_6_): 170.92, 170.41, 144.33, 143.98, 143.40, 143.19, 141.13, 130.87, 130.79, 130.47, 130.40, 130.32, 130.12, 130.01, 129.91, 129.84, 129.71, 129.27, 126.91, 126.81, 121.20, 116.30, 116.09, 56.50, 56.26, 56.12, 55.93, 55.52, 50.47, 50.34, 50.21, 49.50, 48.69, 47.81, 34.77, 34.29, 34.08, 33.88, 16.72. ESI-HRMS (*m*/*z*) [M + H]^+^: calcd for C_28_H_32_FN_4_O_3_S 523.2179; found 523.2183.

#### 2-((2-Cyanoethyl)(phenethyl)amino)-*N*-(naphthalen-2-ylmethyl)-*N*-(4-sulfamoylphenethyl)acetamide (**37**)

Compound **37** was obtained according to the general procedure earlier reported using 2-chloro-*N*-(4-fluorobenzyl)-*N*-(4-sulfamoylphenethyl)acetamide **12** and 3-(phenethylamino)propanenitrile (1.1 equiv) in dry MeCN (5 mL) and stirring for 20 h at reflux temperature. The sticky residue was purified by flash chromatography (1% MeOH in DCM) to give **37** as an oil. Yield 73%; silica gel TLC *R*_*f*_ 0.38 (TFA/MeOH/DCM 3/5/92% v/v). δH (400 MHz, DMSO-*d*_6_): 7.89 (m, 3H, Ar-*H*)7.73 (t, *J* = 9.7 Hz, 3H, Ar-*H*), 7.30 (m, 10H, Ar-*H*), 7.28 (s, 2H, exchange with D_2_O, SO_2_N*H*_2_, overlap with signal at 7.30), 4.75 (m, 2H, C*H*_2_), 3.49 (m, 4H, 2 × C*H*_2_), 2.84 (m, 8H, 4 × C*H*_2_), 2.58 (m, 2H, C*H*_2_). δC (100 MHz, DMSO-*d*_6_): 171.13, 171.01, 167.33, 144.40, 144.18, 144.04, 143.68, 143.51, 143.44, 143.41, 143.29, 143.16, 141.30, 141.14, 141.09, 136.74, 136.45, 136.16, 135.55, 134.10, 134.00, 133.95, 133.36, 133.25, 130.46, 130.42, 130.18, 130.14, 129.72, 129.67, 129.31, 129.28, 129.23, 129.18, 129.16, 128.71, 128.62, 127.44, 127.36, 127.29, 127.14, 127.08, 126.98, 126.93, 126.90, 126.87, 126.83, 126.48, 126.39, 126.24, 125.90, 121.24, 55.98, 51.34, 50.26, 48.68, 45.71, 43.23, 37.03, 34.77, 34.27, 33.95, 33.64, 31.76, 18.84, 16.79, 16.74, 16.70. ESI-HRMS (*m*/*z*) [M + H]^+^: calcd for C_32_H_35_N_4_O_3_S 555.2430; found 555.2425.

#### 2-((2-Cyanoethyl)(phenethyl)amino)-*N*-(4-nitrobenzyl)-*N*-(4-sulfamoylphenethyl)acetamide (**38**)

Compound **38** was obtained according to the general procedure earlier reported using 2-chloro-*N*-(4-nitrobenzyl)-*N*-(4-sulfamoylphenethyl)acetamide **10** and 3-(phenethylamino)propanenitrile (1.1 equiv) in dry MeCN (5 mL) and stirring for 24 h at reflux temperature. The sticky residue was purified by flash chromatography (1% MeOH in DCM) to give **38** as a powder. Yield 51%; mp 108–110 °C; silica gel TLC *R*_*f*_ 0.09 (TFA/MeOH/DCM 3/5/92% v/v). δH (400 MHz, DMSO-*d*_6_): 8.14 (m, 3H, Ar-*H*), 7.71 (m, 3H, Ar-*H*), 7.32 (m, 7H, Ar-*H*), 7.27 (s, 2H, exchange with D_2_O, SO_2_N*H*_2_, overlap with signal at 7.32), 4.67 (s, 1.1H, Ar-*H*), 4.62 (s, 0.9H, C*H*_2_), 3.49 (s, 1H, C*H*_2_), 3.46 (m, 2H, C*H*_2_), 3.26 (s, 1H, C*H*_2_) 2.81 (m, 6H, 3 × C*H*_2_). δC (100 MHz, DMSO-*d*_6_): 170.81, 170.36, 144.67, 144.48, 143.87, 142.53, 141.34, 130.88, 130.68, 130.41, 130.29, 130.15, 129.21, 128.43, 126.82, 126.64, 124.73, 124.60, 124.30, 124.26, 121.61, 116.46, 116.17, 56.32, 56.21, 56.07, 55.84, 55.41, 50.59, 50.33, 50.04, 49.72, 48.65, 47.29, 34.72, 34.24, 34.11, 33.66, 16.43. ESI-HRMS (*m*/*z*) [M + H]^+^: calcd for C_28_H_32_N_5_O_5_S 550.2124; found 550.2119.

#### *N*-(2-Cyanoethyl)-2-((2-cyanoethyl)(phenethyl)amino)-*N*-(4-sulfamoylphenethyl)acetamide (**39**)

Compound **39** was obtained according to the general procedure earlier reported using 2-chloro-*N*-(2-cyanoethyl)-*N*-(4-sulfamoylphenethyl)acetamide **15** and 3-(phenethylamino)propanenitrile (1.1 equiv) in MeCN dry (5 mL) and stirring for 14 h at reflux temperature. The sticky residue was purified by flash chromatography (1% MeOH in DCM) to give **39** as an oil. Yield 75%; silica gel TLC *R*_*f*_ 0.24 (TFA/MeOH/DCM 3/5/92% v/v). δH (400 MHz, DMSO-*d*_6_): 7.76 (m, 2H, Ar-*H*), 7.35 (m, 7H, Ar-*H*), 7.30 (s, 2H, exchange with D_2_O, SO_2_N*H*_2_, overlap with signal at 7.35), 3,55 (m, 6H, 3 × C*H*_2_), 2.82 (m, 10H, 5 × C*H*_2_). δC (100 MHz, DMSO-*d*_6_): 167.38, 167.09, 144.29, 144.09, 143.83, 143.50, 143.47, 143.24, 138.62, 130.54, 130.50, 130.43, 130.28, 130.23, 129.77, 129.33, 129.10, 126.84, 126.82, 126.58, 120.21, 120.04, 56.21, 50.23, 49.92, 49.14, 47.96, 47.42, 44.10, 43.46, 43.21, 43.03, 42.82, 42.17, 34.99, 33.84, 21.84, 18.01, 17.88, 16.59, 16.43. ESI-HRMS (*m*/*z*) [M + H]^+^: calcd for C_24_H_30_N_5_O_3_S 468.2069; found 468.2073.

### General Synthesis Procedure of Amine Derivatives **40–44**

To a solution of nitrile derivatives **33–39** (0.5 mmol, 1.0 equiv) and 5 M NaOH_(aq)_ (3.0 equiv) in EtOH (10 mL), Ni/Raney (0.5 mL) was added, and the mixture was stirred o.n. under H_2_ pressure (50 psi). The solution was filtered off, and the solvent was evaporated under *vacuum*. The residue was purified by flash chromatography (5–15% MeOH in DCM) to give compounds **40–44**.

#### 2-((3-Aminopropyl)(phenethyl)amino)-*N*-phenethyl-*N*-(4-sulfamoylphenethyl)acetamide (**40**)

Compound **40** was obtained according to the general procedure earlier reported using 2-((2-cyanoethyl)(phenethyl)amino)-*N*-phenethyl-*N*-(4-sulfamoylphenethyl)acetamide **34**. The obtained residue was purified by flash chromatography to give **40** as an oil. Yield 26%; silica gel TLC *R*_*f*_ 0.38 (TFA/MeOH/DCM 3/5/92% v/v). δH (400 MHz, DMSO-*d*_6_): 7.76 (m, 4H, Ar-*H*), 7.44 (m, 4H, Ar-*H*), 7.23 (s, 2H, exchange with D_2_O, SO_2_N*H*_2_, overlap with signal at 7.20), 7.20 (m, 11H, Ar-*H*), 3.50 (m, 4H, 2 × C*H*_2_), 3.19 (s, 1.1H, C*H*_2_), 3.09 (s, 0.9H, C*H*_2_), 2.75 (m, 10H, 5 × C*H*_2_), 2.34 (m, 2H, C*H*_2_), 1.60 (m, 2H, C*H*_2_). δC (100 MHz, DMSO-*d*_6_): 171.90, 171.75, 144.45, 143.88, 143.51, 143.24, 141.28, 141.22, 140.24, 139.80, 130.54, 130.26, 130.16, 129.78, 129.75, 129.50, 129.42, 129.31, 127.49, 127.24, 126.95, 126.81, 126.77, 56.63, 56.40, 55.82, 52.80, 52.70, 49.06, 48.74, 47.95, 47.42, 39.56, 35.12, 34.89, 34.16, 33.88, 33.29, 33.23, 24.57, 23.08. ESI-HRMS (*m*/*z*) [M + H]^+^: calcd for C_29_H_39_N_4_O_3_S 523.2743; found 523.2748.

#### 2-((3-Aminopropyl)(phenethyl)amino)-*N*-(furan-2-ylmethyl)-*N*-(4-sulfamoylphenethyl)acetamide (**41**)

Compound **41** was obtained according to the general procedure earlier reported using 2-((2-cyanoethyl)(phenethyl)amino)-*N*-(furan-2-ylmethyl)-*N*-(4-sulfamoylphenethyl)acetamide **35**. The obtained residue was purified by flash chromatography to give **41** as an oil. Yield 33%; silica gel TLC *R*_*f*_ 0.42 (TFA/MeOH/DCM 3/5/92% v/v). δH (400 MHz, DMSO-*d*_6_): 7.75 (t, J = 8.4 Hz, 2H, Ar-*H*), 7.67 (s, 0.5H, Ar-*H*), 7.62 (s, 0.5H, Ar-*H*), 7.29 (m, 7H, Ar-*H*), 7.20 (s, 2H, exchange with D_2_O, SO_2_N*H*_2_, overlap with signal at 7.29), 6.44 (m, 2H, Ar-*H*), 4.60 (m, 2H, C*H*_2_), 3.57 (s, 2H, C*H*_2_), 3.49 (m, 2H, C*H*_2_), 3.26 (s, 2H, C*H*_2_), 2.75 (m, 7H, 4 × C*H*_2_), 2.34 (m, 1H, C*H*_2_), 1.65 (m, 2H, C*H*_2_). δC (100 MHz, DMSO-*d*_6_): 172.04, 171,99, 151.83, 151.55, 144.24, 144.20, 143.77, 143.69, 143.56, 143.25, 141.15, 130.50, 130.16, 129.75, 129.34, 129.31, 127.00, 126.88, 126.82, 111.71, 111.67, 109.73, 109.69, 56.71, 56.51, 55.85, 55.67, 53.17, 52.75, 48.22, 47.96, 44.39, 42.03, 39.64, 34.47, 33.78, 33.30, 33.19, 24.31, 24.22. ESI-HRMS (*m*/*z*) [M + H]^+^: calcd for C_26_H_35_N_4_O_4_S 499.2379; found 499.2373.

#### 2-((3-Aminopropyl)(phenethyl)amino)-*N*-(4-fluorobenzyl)-*N*-(4-sulfamoylphenethyl)acetamide (**42**)

Compound **42** was obtained according to the general procedure earlier reported using 2-((2-cyanoethyl)(phenethyl)amino)-*N*-(4-fluorobenzyl)-*N*-(4-sulfamoylphenethyl)acetamide **36**. The obtained solid was purified by flash chromatography to give **42** as an oil. Yield 28%; silica gel TLC *R*_*f*_ 0.37 (TFA/MeOH/DCM 3/5/92% v/v). δH (400 MHz, DMSO-*d*_6_): 7.77 (m, 2H, Ar-*H*), 7.30 (m, 11H, Ar-*H*), 7.21 (s, 2H, exchange with D_2_O, SO_2_N*H*_2_, overlap with signal at 7.30), 4.60 (m, 2H, C*H*_2_), 3.48 (m, 6H, 3 × C*H*_2_), 2.83 (m, 7H, 4 × C*H*_2_), 2.42 (m, 1H, C*H*_2_), 1.68 (m, 2H, C*H*_2_). δF (376 MHz, DMSO-*d*_6_): −115.42, −115.62. δC (100 MHz, DMSO-*d*_6_): 172.31, 163.69, 161.11, 144.28, 143.76, 143.58, 143.26, 141.22, 141.13, 134.99, 134.97, 134.43, 134.41, 130.79, 130.71, 130.50, 130.18, 129.97, 129.89, 129.75, 129.71, 129.31, 126.96, 126.88, 126.82, 116.73, 116.52, 116.40, 116.19, 56.82, 56.47, 55.73, 53.01, 52.80, 50.21, 48.21, 48.00, 39.62, 39.61, 34.52, 33.84, 33.23, 24.54, 24.39. ESI-HRMS (*m*/*z*) [M + H]^+^: calcd for C_28_H_36_FN_4_O_3_S 527.2492; found 527.2488.

#### 2-((3-Aminopropyl)(phenethyl)amino)-*N*-(naphthalen-2-ylmethyl)-*N*-(4-sulfamoylphenethyl)acetamide (**43**)

Compound **43** was obtained according to the general procedure earlier reported using 2-((2-cyanoethyl)(phenethyl)amino)-*N*-(naphthalen-2-ylmethyl)-*N*-(4-sulfamoylphenethyl)acetamide **37**. The obtained solid was purified by flash chromatography to give **43** as an oil. Yield 34%; silica gel TLC *R*_*f*_ 0.42 (TFA/MeOH/DCM 3/5/92% v/v). δH (400 MHz, DMSO-*d*_6_): 7.91 (m, 3H, Ar-*H*), 7.76 (m, 3H, Ar-*H*), 7.28 (m, 10H, Ar-*H*), 7.20 (s, 2H, exchange with D_2_O, SO_2_N*H*_2_, overlap with signal at 7.28), 4.79 (m, 2H, C*H*_2_), 3.52 (m, 4H, 2 × C*H*_2_), 2.91 (m, 4H, 2 × C*H*_2_), 2.66 (m, 6H, 3 × C*H*_2_), 1.70 (m, 2H, C*H*_2_). δC (100 MHz, DMSO-*d*_6_): 172.58, 172.30, 163.07, 161.66, 144.37, 143.97, 143.60, 143.32, 141.33, 134.21, 134.00, 133.51, 133.34, 130.55, 130.22, 130.16, 129.77, 129.68, 129.57, 129.31, 129.27, 129.19, 128.73, 128.63, 127.53, 127.35, 127.10, 126.96, 126.91, 126.81, 126.17, 126.13, 126.02, 125.27, 56.76, 56.41, 55.89, 55.76, 53.20, 52.91, 52.69, 51.09, 49.69, 49.25, 48.60, 39.62, 33.93, 33.27, 32.59, 32.46, 28.12, 21.21. ESI-HRMS (*m*/*z*) [M + H]^+^: calcd for C_32_H_39_N_4_O_3_S 559.2743; found 559.2737.

#### *N*-(3-Aminopropyl)-2-(dihexylamino)-*N*-(4-sulfamoylphenethyl)acetamide (**44**)

Compound **44** was obtained according to the general procedure earlier reported using *N*-(2-cyanoethyl)-2-(dihexylamino)-*N*-(4-sulfamoylphenethyl)acetamide **33**. The obtained solid was purified by flash chromatography to give **44** as an oil. Yield 31%; silica gel TLC *R*_*f*_ 0.43 (TFA/MeOH/DCM 3/5/92% v/v). δH (400 MHz, DMSO-*d*_6_): 7.78 (d, *J* = 8.0 Hz, 2H, Ar-*H*), 7.49 (d, *J* = 8.0 Hz, 2H, Ar-*H*), 7.35 (s, 2H, exchange with D_2_O, SO_2_N*H*_2_), 3.48 (m, 2H, C*H*_2_), 3.20 (m, 2H, C*H*_2_), 2.88 (m, 8H, 4 × C*H*_2_), 1.85 (m, 4H, 2 × C*H*_2_), 1.45 (m, 4H, 2 × C*H*_2_), 1.25 (m, 12H, 6 × C*H*_2_), 0.86 (m, 6H, 2 × C*H*_3_). δC (100 MHz, DMSO-*d*_6_): 169.55, 169.26, 143.81, 143.54, 135.94, 133.31, 131.36, 130.47, 130.20, 126.81, 55.06, 55.00, 48.79, 47.51, 37.69, 37.45, 37.41, 34.96, 34.74, 33.94, 33.80, 31.96, 31.94, 28.69, 27.89, 27.31, 27.17, 27.03, 26.88, 26.30, 23.05, 23.02, 14.93, 14.92. ESI-HRMS (*m*/*z*) [M + H]^+^: calcd for C_25_H_47_N_4_O_3_S 483.3369; found 483.3374.

### General Synthesis Procedure of Carboxylic Acid Derivatives **45–49**

To a solution of the appropriate nitrile derivatives **33–39** (0.5 mmol, 1.0 equiv) in EtOH (5 mL), 5 M NaOH_(aq)_ (3.0 equiv) was added, and the mixture was heated at reflux temperature under stirring o.n.. The solution was cooled to 0 °C and 12 M HCl (2.0 equiv) was added dropwise until precipitation of a powder that was collected by filtration. The solid was purified by flash chromatography (5–15% MeOH in DCM) to give the compounds **45–49**.

#### 3-((2-Oxo-2-(phenethyl(4-sulfamoylphenethyl)amino)ethyl)(phenethyl)amino)propanoic acid (**45**)

Compound **45** was obtained according to the general procedure earlier reported using 2-((2-cyanoethyl)(phenethyl)amino)-*N*-phenethyl-*N*-(4-sulfamoylphenethyl)acetamide **34**. The obtained solid was purified by flash chromatography to give **45** as a powder. Yield 31%; mp 74–76 °C; silica gel TLC *R*_*f*_ 0.35 (TFA/MeOH/DCM 3/5/92% v/v). δH (400 MHz, DMSO-*d*_6_): 12.05 (brs, 1H, exchange with D_2_O, COO*H*), 7.72 (m, 3H, Ar-*H*), 7.27 (m, 16H, Ar-*H*), 7.23 (s, 2H, exchange with D_2_O, SO_2_N*H*_2_, overlap with signal at 7.27), 3.46 (m, 4H, 2 × C*H*_2_), 3.14 (s, 0.9H, C*H*_2_), 3.09 (s, 1.1H, C*H*_2_), 2.72 (m, 10H, 5x C*H*_2_), 2.25 (s, 2H, C*H*_2_). δC (100 MHz, DMSO-*d*_6_): 175.20, 175.13, 170.64, 170.52, 167.24, 143.38, 143.18, 142.84, 142.84, 141.41, 141.31, 141.31, 130.43, 130.19, 130.11, 130.05, 129.74, 129.67, 129.65, 129.45, 129.41, 129.29, 127.40, 127.21, 126.89, 126.81, 126.78, 126.65, 57.46, 57.04, 56.40, 51.81, 51.39, 50.34, 49.51, 49.15, 47.86, 47.58, 36.78, 36.48, 35.36, 35.14, 34.17, 33.92, 33.33. ESI-HRMS (*m*/*z*) [M + H]^+^: calcd for C_29_H_36_N_3_O_5_S 538.2376; found 538.2381.

#### 3-((2-((Furan-2-ylmethyl)(4-sulfamoylphenethyl)amino)-2-oxoethyl)(phenethyl)amino)propanoic acid (**46**)

Compound **46** was obtained according to the general procedure earlier reported using 2-((2-cyanoethyl)(phenethyl)amino)-*N*-(furan-2-ylmethyl)-*N*-(4-sulfamoylphenethyl)acetamide **35**. The obtained solid was purified by flash chromatography to give **46** as a powder. Yield 35%; mp 33–35 °C; silica gel TLC *R*_*f*_ 0.39 (TFA/MeOH/DCM 3/5/92% v/v). δH (400 MHz, DMSO-*d*_6_): 12.01 (brs, 1H, exchange with D_2_O, COO*H*), 7.74 (t, *J* = 7.3 Hz, 2H, Ar-*H*), 7.64 (s, 0.5H, Ar-*H*), 7.57 (s, 0.5H, Ar-*H*), 7.25 (m, 7H, Ar-*H*), 7.21 (s, 2H, exchange with D_2_O, SO_2_N*H*_2_, overlap with signal at 7.25), 6.39 (m, 2H, Ar-*H*), 4.54 (m, 2H, C*H*_2_), 3.48 (s, 2H, C*H*_2_), 3.38 (m, 2H, C*H*_2_), 3.27 (s, 2H, C*H*_2_), 2.76 (m, 6H, 3 × C*H*_2_), 2.35 (m, 2H, C*H*_2_). δC (100 MHz, DMSO-*d*_6_): 174.69, 174.67, 170.58, 152.17, 152.06, 144.35, 144.06, 144.03, 143.49, 143.38, 143.18, 141.33, 141.22, 130.33, 130.06, 129.70, 129.66, 129.28, 126.86, 111.65, 111.59, 109.46, 109.37, 57.46, 57.32, 56.26, 50.31, 50.16, 48.59, 47.95, 44.57, 41.76, 34.77, 33.80, 33.60, 32.95, 32.87, 31.76. ESI-HRMS (*m*/*z*) [M + H]^+^: calcd for C_26_H_32_N_3_O_6_S 514.2012; found 514.2008.

#### 3-((2-((4-Fluorobenzyl)(4-sulfamoylphenethyl)amino)-2-oxoethyl)(phenethyl)amino)propanoic acid (**47**)

Compound **47** was obtained according to the general procedure earlier reported using 2-((2-cyanoethyl)(phenethyl)amino)-*N*-(4-fluorobenzyl)-*N*-(4-sulfamoylphenethyl)acetamide **36**. The obtained solid was purified by flash chromatography to give **47** as a powder. Yield 33%; mp 64–66 °C; silica gel TLC *R*_*f*_ 0.41 (TFA/MeOH/DCM 3/5/92% v/v). δH (400 MHz, DMSO-*d*_6_): 11.82 (brs, 1H, exchange with D_2_O, COO*H*), 7.73 (m, 2H, Ar-*H*), 7.24 (m, 11H, Ar-*H*), 7.21 (s, 2H, exchange with D_2_O, SO_2_N*H*_2_, overlap with signal at 7.24), 4.53 (m, 2H, C*H*_2_), 3.39 (s, 6H, 3 × C*H*_2_), 2.80 (m, 6H, 3 × C*H*_2_), 2.36 (m, 2H, C*H*_2_). δF (376 MHz, DMSO-*d*_6_): −115.43, −115.60. δC (100 MHz, DMSO-*d*_6_): 174.66, 174.63, 173.07, 170.88, 163.52, 161.23, 144.37, 144.02, 143.38, 143.19, 141.24, 141.16, 135.44, 134.93, 131.42, 130.84, 130.76, 130.36, 130.20, 130.07, 129.94, 129.67, 129.65, 129.28, 126.93, 126.89, 126.82, 126.63, 116.59, 116.38, 116.28, 116.07, 65.98, 57.24, 56.27, 56.19, 50.23, 50.10, 48.62, 47.81, 34.83, 33.84, 33.52, 32.84, 22.12, 16.23. ESI-HRMS (*m*/*z*) [M + H]^+^: calcd for C_28_H_33_FN_3_O_5_S 542.2125; found 542.2131.

#### 3-((2-((Naphthalen-2-ylmethyl)(4-sulfamoylphenethyl)amino)-2-oxoethyl)(phenethyl)amino)propanoic acid (**48**)

Compound **48** was obtained according to the general procedure earlier reported using 2-((2-cyanoethyl)(phenethyl)amino)-*N*-(naphthalen-2-ylmethyl)-*N*-(4-sulfamoylphenethyl)acetamide **37**. The obtained solid was purified by flash chromatography to give **48** as a powder. Yield 37%; mp 96–98 °C; silica gel TLC *R*_*f*_ 0.43 (TFA/MeOH/DCM 3/5/92% v/v). δH (400 MHz, DMSO-*d*_6_): 12.34 (brs, 1H, exchange with D_2_O, COO*H*), 7.88 (m, 3H, Ar-*H*), 7.72 (m, 3H, Ar-*H*), 7.32 (m, 10H, Ar-*H*), 7.22 (s, 2H, exchange with D_2_O, SO_2_N*H*_2_, overlap with signal at 7.32), 3.48 (m, 3.1H, 2 × C*H*_2_), 3.17 (s, 0.9H, C*H*_2_), 2.75 (m, 8H, 4 × C*H*_2_), 2.32 (m, 2H, C*H*_2_). δC (100 MHz, DMSO-*d*_6_): 175.33, 175.19, 171.15, 171.11, 144.40, 144.12, 143.40, 143.18, 141.34, 141.22, 136.77, 136.44, 134.12, 133.95, 133.32, 133.25, 130.38, 130.10, 129.65, 129.41, 129.28, 129.25, 129.15, 128.71, 128.64, 128.61, 127.42, 127.29, 127.14, 127.10, 126.96, 126.83, 126.28, 125.95, 57.54, 57.38, 56.29, 56.26, 51.41, 50.52, 50.34, 49.67, 48.66, 48.64, 48.13, 48.12, 34.93, 33.97, 33.48, 33.35, 33.31. ESI-HRMS (*m*/*z*) [M + H]^+^: calcd for C_32_H_36_N_3_O_5_S 574.2376; found 574.2371.

#### 3-(2-(Dihexylamino)-*N*-(4-sulfamoylphenethyl)acetamido)propanoic Acid (**49**)

Compound **49** was obtained according to the general procedure earlier reported using *N*-(2-cyanoethyl)-2-(dihexylamino)-*N*-(4-sulfamoylphenethyl)acetamide **33**. The obtained solid was purified by flash chromatography to give **49** as a powder. Yield 33%; mp > 300 °C; silica gel TLC *R*_*f*_ 0.36 (TFA/MeOH/DCM 3/5/92% v/v). δH (400 MHz, DMSO-*d*_6_): 12.14 (brs, 1H, exchange with D_2_O, COO*H*), 7.73 (d, *J* = 8.0 Hz, 2H, Ar-*H*), 7.40 (d, *J* = 8.0 Hz, 2H, Ar-*H*), 7.31 (s, 2H, exchange with D_2_O, SO_2_N*H*_2_), 3.66 (m, 2H, C*H*_2_), 3.48 (m, 2H, C*H*_2_), 3.25 (s, 1.1H, C*H*_2_), 2.99 (s, 0.9H, C*H*_2_), 2.91 (m, 1H, C*H*_2_), 2.79 (m, 1H, C*H*_2_), 2.42 (m, 2H, C*H*_2_), 2.32 (m, 2H, C*H*_2_), 2.10 (m, 2H, C*H*_2_), 1.30 (m, 16H, 8 × C*H*_2_), 0.84 (m, 6H, 2 × C*H*_3_). δC (100 MHz, DMSO-*d*_6_): 175.95, 175.28, 170.94, 170.61, 144.84, 144.49, 143.37, 143.20, 130.27, 130.11, 126.80, 126.76, 59.22, 57.99, 54.91, 54.80, 48.91, 47.61, 38.89, 35.54, 34.23, 32.27, 32.20, 27.67, 27.64, 27.52, 27.35, 25.62, 23.19, 23.14, 14.99, 14.95. ESI-HRMS (*m*/*z*) [M + H]^+^: calcd for C_25_H_44_N_3_O_5_S 498.3001; found 498.2997.

#### Synthesis of (*Z*)-3-((2-((furan-2-ylmethyl)(4-sulfamoylphenethyl)amino)-2-oxoethyl)(phenethyl)amino)-*N*-(octadec-9-en-1-yl)propanamide (**50**)

To a solution of **46** (0.5 mmol, 1.0 eq) in DMF dry (1 mL), oleylamine (1.1 eq), EDC·HCl (1.2 eq), and DMAP (catalytic) were added, and the reaction mixture was stirred at r.t. for 6 h. The reaction was quenched with water and extracted with EtOAc (15 mL × 3). The organic layers were washed with brine (20 mL × 4), dried over Na_2_SO_4_, filtered off, and evaporated under *vacuum*. The obtained residue was purified by flash chromatography (3% MeOH in DCM) to give compound **50** as an oil. Yield 73%; silica gel TLC *R*_*f*_ 0.29 (TFA/MeOH/DCM 3/5/92% v/v). δH (400 MHz, DMSO-*d*_6_): 7.93 (s, 1H, exchange with D_2_O, CON*H*), 7.73 (t, J = 7.2 Hz, 2H, Ar-*H*), 7.63 (s, 0.5H, Ar-*H*), 7.57 (s, 0.5H, Ar-*H*), 7.27 (m, 7H, Ar-*H*), 7.21 (s, 2H, exchange with D_2_O, SO_2_N*H*_2_, overlap with signal at 7.27), 6.39 (m, 2H, Ar-*H*), 5.31 (m, 2H, 2 × =C*H*), 4.55 (s, 2H, C*H*_2_) 3.48 (m, 3.1 H, 2 × C*H*_2_), 3.21 (s, 0.9H, C*H*_2_), 2.99 (m, 2H, C*H*_2_), 2.73 (m, 10H, 5 × C*H*_2_), 2.21 (m, 2H, C*H*_2_), 1.97 (m, 4H, 2 × C*H*_2_), 1.29 (m, 22H, 11 × C*H*_2_), 0.83 (m, 3H, C*H*_3_). δC (100 MHz, DMSO-*d*_6_): 171.94, 171.93, 171.89, 171.88, 170.88, 170.76, 170.75, 170.69, 152.29, 152.07, 144.44, 144.16, 143.40, 143.12, 141.47, 141.33, 131.14, 130.69, 130.34, 130.04, 129.68, 129.63, 129.23, 126.84, 126.81, 111.61, 111.57, 109.42, 109.39, 65.97, 57.81, 57.55, 56.03, 55.96, 55.41, 50.91, 50.82, 48.61, 47.91, 44.54, 39.49, 34.27, 34.20, 34.18, 33.81, 33.55, 33.52, 33.49, 32.94, 32.32, 30.17, 30.14, 30.13, 30.08, 30.05, 29.94, 29.88, 29.82, 29.73, 29.63, 29.51, 27.67, 27.61, 27.50, 23.13, 16.23, 15.00. ESI-HRMS (*m*/*z*) [M + H]^+^: calcd for C_44_H_67_N_4_O_5_S 763.4832; found 763.4826.

### Carbonic Anhydrase Inhibition

An Applied Photophysics stopped-flow instrument has been used for assaying the CA-catalyzed CO_2_ hydration activity.^34^ Phenol red (at a concentration of 0.2 mM) has been used as an indicator, working at the absorbance maximum of 557 nm, with 20 mM Hepes (pH 7.5) as a buffer and 20 mM Na_2_SO_4_ (for maintaining the ionic strength constant), following the initial rates of the CA-catalyzed CO_2_ hydration reaction for a period of 10–100 s. The CO_2_ concentrations ranged from 1.7 to 17 mM for the determination of the kinetic parameters and inhibition constants. For each inhibitor, at least six traces of the initial 5–10% of the reaction have been used for determining the initial velocity. The uncatalyzed rates were determined in the same manner and subtracted from the total observed rates. Stock solutions of inhibitor (0.1 mM) were prepared in distilled–deionized water, and dilutions up to 0.01 nM were done thereafter with the assay buffer. Inhibitor and enzyme solutions were preincubated together for 15 min at room temperature prior to assay to allow the formation of the E–I complex. The inhibition constants were obtained by nonlinear least-squares methods using PRISM 3 and the Cheng–Prusoff equation, as reported earlier,^36^ and represent the mean from at least three different determinations. All hCA isofoms were recombinant ones obtained in-house as reported earlier.^49^

### X-ray Crystallography

#### Protein Expression and Purification

Competent BL21 *Escherichia coli* cells were transformed separately with plasmid DNA containing the hCA II gene using standard protocols.^50,51^ An overnight culture in LB was started with large-scale growth the following day until OD_600_ reached ∼0.6. Isopropyl β-d-1-thiogalactoside (IPTG, 0.5 mM) and zinc sulfate (1 mM) were used to induce protein expression for 3 h. The cells were pelleted and lysed via a microfluidizer set to 18 000 PSI. Supernatant was filtered with a 0.4 μm filter before being run through an affinity column with *p*-aminomethyl-benzenesulfonamide agarose. Enzyme was eluted with azide and buffer-exchanged into storage buffer (50 mM Tris pH 7.8) to remove azide. The purity of the protein was determined by a 12% sodium dodecyl sulfate polyacrylamide gel electrophoresis (SDS-PAGE) and UV/vis spectroscopy at a 280 nm measured protein concentration.

#### Crystallization

Inhibitors were successfully co-crystallized with hCA II via the hanging-drop vapor diffusion method. Mother liquor (500 μL) consisting of 1.6 M sodium citrate and 50 mM Tris at pH 7.8 was used in setting up crystal trays for each well. Each drop contained a 1:1 ratio of 10 mg/mL protein to mother liquor. DMSO was used to dissolve inhibitors to 1 mM, with the drops’ final concentration ∼100 μM. Co-crystals of hCA II formed within a week.

#### Data Collection and Processing

Diffraction data were collected via the F1 beamline at Cornell High Energy Synchrotron Source (CHESS) at 0.977 Å wavelength. A Pilatus 6M detector collected data sets with a crystal-to-detector distance of 270 mm, 1° oscillation, and 4 s image exposure, for a total of 180 images. Diffraction data were indexed and integrated with XDS.^52^ Data were scaled in space group *P*2_1_ via AIMLESS^53^ from the CCP4 program suite.^54^ Phases were determined via molecular replacement using PDB: 3KS3(55) as a search model. Modifications to the model such as addition of inhibitor, ligand (glycerol), zinc, and water to the active site were executed in Coot^56^ along with ligand PDB file modifications. Refinements were completed and ligand restraint files were created in Phenix.^57^ Figures were generated with PyMol (Schrödinger). Protein–ligand bond lengths and active site interactions were observed with LigPlot Plus.^58^

#### Computational Study

HCA I (PDB: 2NMX),^43^ hCA II (PDB: 5LJT),^46^ hCA IV (PDB: 1ZNC),^44^ and hCA XII (PDB: 1JD0)^45^ crystal structures were prepared according to the Protein Preparation module in Maestro-Schrödinger suite, assigning bond orders, adding hydrogens, deleting water molecules, and optimizing H-bonding networks.^59^ Finally, energy minimization with a root-mean-square deviation (RMSD) value of 0.30 was applied using an Optimized Potentials for Liquid Simulation (OPLS-3) force field. Input 3D ligand structures were prepared by Maestro^59a^ and evaluated for their ionization states with Epik.^59b^ Sulfonamides were considered in their deprotonated form on the basis of evidence from neutron crystallography. OPLS-3 force field in Macromodel^59c^ was used for energy minimization for a maximum number of 2500 conjugate gradient iteration and setting a convergence criterion of 0.05 kcal mol^–1^ Å^–1^. The docking grid was generated using Glide^59d^ with default settings, with the center located on the center of mass of the co-crystallized ligand. Ligands were docked with the standard precision (SP) mode of Glide and the five top-scoring poses of each molecule retained as output. The best pose for each compound, evaluated in terms of coordination, hydrogen-bond interactions, and hydrophobic contacts, was refined by Prime MM-GBSA methods using a VSGB solvation model.^60−63^

#### Hypertensive Rabbit IOP Lowering Studies

Male New Zealand albino rabbits weighing 1500–2000 g were used in these studies. Animals were anesthetized using Zoletil (tiletamine chloride plus zolazepam chloride, 3 mg/kg body weight, im), and elevated IOP was induced by the injection of 0.05 mL of hypertonic saline solution (5% in distilled water) into the vitreous of both eyes. IOP was determined using a pneumo-tonometer Reichert, model 30 (Reichert, Inc., Depew, NY) prior to hypertonic saline injection (basal), and at 1, 2, 3, and 4 h after administration of the different drugs. Vehicle (hydroxypropylcellulose at 0.05%) or drugs were instilled immediately after the injection of hypertonic saline. Eyes were randomly assigned to different groups. Vehicle or drug (0.05 mL) was directly instilled into the conjunctive pocket at the desired doses (1–2%).^64^ Four different animals were used for each tested compound. All animal manipulations were carried out according to the European Community guidelines for animal care [DL 116/92, application of the European Communities Council Directive of 24 November 1986 (86/609/EEC)]. The ethical policy of the University of Florence complies with the Guide for the Care and Use of Laboratory Animals of the US National Institutes of Health (NIH Publication no. 85–23, revised 1996; University of Florence assurance number A5278-01). Formal approval to conduct the experiments described was obtained from the Animal Subjects Review Board of the University of Florence and upon authorization of the National Ethics Committee of the Italian Ministry of Health (number 1179/2015-PR). Experiments involving animals have been reported according to ARRIVE, Animal Research: Reporting of in Vivo Experiments, guidelines.^65^ All efforts were made to minimize animal suffering and to reduce the number of animals used.

## Supporting Information Available

^1^H and ^13^C NMR spectra, selectivity index (SI) of carbonic anhydrases inhibition, supplemental molecular modeling figures, and HPLC chromatograms (PDF)
SMILES representation for compounds **18**–**50** (CSV)

## Accession Codes

The atomic coordinates of the complexes have been deposited in the Protein Data Bank with accession codes 6WQ4, 6WQ5, 6WQ7, 6WQ8, and 6WQ9.

## Abbreviations Used

MeOH: Methanol
NaBH_4_: sodium borohydride
r.t.: room temperature
Et_3_N: triethylamine
MeCN: acetonitrile
NaOH: sodium hydroxide
EtOH: ethanol
o.n.: overnight
EDC·HCl: *N*-(3-dimethylaminopropyl)-*N*′-ethylcarbodiimide hydrochloride
DMAP: 4-dimethylaminopyridine
DMF: *N*,*N*-dimethylformamide
DCM: dichloromethane
TFA: trifluoroacetic acid
DMSO: dimethylsulfoxide
EtOAc: ethyl acetate
Et_2_O: diethyl ether
