# Supplementary material for: Sulfonamide Inhibitors of Human Carbonic Anhydrases Designed through a Three-Tails Approach: Improving Ligand/Isoform Matching and Selectivity of Action
Source: J Med Chem. 2020 Jun 10;63(13):7422–44. doi: 10.1021/acs.jmedchem.0c00733 (PMC8008423; doi:10.1021/acs.jmedchem.0c00733)
Supplement: Supplementary file 1 — jm0c00733_si_001.pdf [file jm0c00733_si_001.pdf]

Supporting Information

Sulfonamide Inhibitors of Human Carbonic Anhydrases Designed through a Three-Tails Approach: Improving Ligand/Isoform Matching and Selectivity of Action

Alessandro Bonardi,[a,b] Alessio Nocentini,[a,b*] Silvia Bua,[a] Jacob Combs,[c] Carrie Lomelino,[c] Jacob Andring,[c] Laura Lucarini,[d] Silvia Sgambellone,[d] Emanuela Masini,[d] Robert McKenna,[c] Paola Gratteri,[a,b] Claudiu T. Supuran[a,*]

[a] *Department NEUROFARBA – Pharmaceutical and nutraceutical section, University of Firenze, via Ugo Schiff 6, 50019 Sesto Fiorentino (Florence), Italy.*

[b] *Department NEUROFARBA – Pharmaceutical and nutraceutical section; Laboratory of Molecular Modeling Cheminformatics & QSAR, University of Firenze, via Ugo Schiff 6, 50019 Sesto Fiorentino (Florence), Italy.*

[c] *Department of Biochemistry and Molecular Biology, College of Medicine, University of Florida, Box 100245, Gainesville, FL 32610, USA.*

[d] *Department NEUROFARBA – Pharmaceutical and nutraceutical section, University of Firenze, viale Gaetano Pieraccini 6, 50139 Firenze (Florence), Italy.*

Selectivity Index (*SI*) of Carbonic Anhydrases Inhibition S2

Supplemental Modelling Figures S3

HPLC chromatograms S7

Table S1. Selective index (*SI*) for the single-tail compounds **1-7** and **TTIs 18-50** calculated as ratio between $K_{I\ CA1}$ and $K_{I\ CA2}$

Cmpd	R_1	R_2	R_3	*SI*					
				CA I/II	CA I/IV	CA II/IV	CA I/XII	CA II/XII	CA IV/XII
**1**	C_6H_5			1.0	0.03	0.03	1.5	1.5	43.6
**2**	4-NO_2-C_6H_4	-	-	1.9	0.1	0.07	2.9	1.6	21.4
**3**	4-F-C_6H_4	-	-	1.4	0.09	0.06	1.9	1.3	19.9
**4**	2-Naph	-	-	5.3	0.07	0.01	5.8	1.1	79.5
**5**	Fu	-	-	1.1	0.04	0.04	1.2	1.1	28.6
**6**	CH_2CN	-	-	0.7	0.02	0.03	0.9	1.4	49.0
**7**	$CH_2C_6H_5$	-	-	3.1	0.08	0.02	2.7	0.9	34.4
**18**	C_6H_5	-	-	94.8	0.2	0.002	17.9	0.2	94.5
**19**	C_6H_5	CH_2CH_3	CH_2CH_3	10.7	<0.4	<0.04	51.0	4.7	>121.1
**20**	C_6H_5	CH_2CH_3	$CH_2C_6H_5$	2.1	<0.09	<0.04	3.7	8.8	>101.2
**21**	C_6H_5	$CH_2C_6H_5$	$CH_2C_6H_5$	4.1	<0.05	<0.01	7.3	1.8	>144.1
**22**	C_6H_5	$(CH_2)_4CH_3$	$(CH_2)_4CH_3$	3.7	<0.09	<0.02	2.6	9.5	>107.8
**23**	C_6H_5	$(CH_2)_5CH_3$	$(CH_2)_5CH_3$	1.1	<0.09	<0.08	9.5	8.5	>100.6
**24**	C_6H_5	$(CH_2)_7CH_3$	$(CH_2)_7CH_3$	20.7	0.04	0.002	3.0	0.1	64.3
**25**	C_6H_5	CH_2CH_3	CH_2CH_3	6.8	<0.05	<0.008	6.0	0.9	>110.6
**26**	C_6H_5	CH_2CH_3	$CH_2C_6H_5$	1.2	0.2	0.1	2.3	1.8	15.3
**27**	C_6H_5	$CH_2C_6H_5$	$CH_2C_6H_5$	1.1	0.2	0.2	5.6	5.2	32.4
**28**	C_6H_5	$(CH_2)_4CH_3$	$(CH_2)_4CH_3$	0.5	0.09	0.2	4.0	7.4	44.4
**29**	C_6H_5	$(CH_2)_5CH_3$	$(CH_2)_5CH_3$	0.3	<0.02	<0.08	0.7	2.8	>35.7
**30**	$CH_2C_6H_5$	$(CH_2)_7CH_3$	$(CH_2)_7CH_3$	0.7	0.2	0.2	8.3	12.5	54.6
**31**	Fu	$(CH_2)_5CH_3$	$(CH_2)_5CH_3$	3.6	0.07	0.02	22.7	6.2	325.1
**32**	2-Naph	$(CH_2)_5CH_3$	$(CH_2)_5CH_3$	0.1	<0.05	<0.5	8.8	74.0	>162.1
**33**	CH_2CN	$(CH_2)_5CH_3$	$(CH_2)_5CH_3$	7.5	0.1	0.02	46.0	6.1	404.5
**34**	$CH_2C_6H_5$	$(CH_2)_5CH_3$	$(CH_2)_5CH_3$	2.1	<0.08	<0.04	10.3	4.9	>132.5
**35**	Fu	$(CH_2)_2C_6H_5$	$(CH_2)_2CN$	4.1	0.7	0.2	34.6	8.4	52.6
**36**	4-F-C_6H_4	$(CH_2)_2C_6H_5$	$(CH_2)_2CN$	5.1	0.2	0.1	69.8	13.6	421.8
**37**	2-Naph	$(CH_2)_2C_6H_5$	$(CH_2)_2CN$	2.8	0.2	0.03	10.5	3.8	58.8
**38**	4-NO_2-C_6H_4	$(CH_2)_2C_6H_5$	$(CH_2)_2CN$	1.5	0.2	0.06	4.6	3.0	27.1
**39**	CH_2CN	$(CH_2)_2C_6H_5$	$(CH_2)_2CN$	88.0	0.08	0.001	6.9	0.08	81.6
**40**	$CH_2C_6H_5$	$(CH_2)_2C_6H_5$	$(CH_2)_2CN$	0.7	0.1	0.2	2.9	4.4	25.7
**41**	Fu	$(CH_2)_2C_6H_5$	$(CH_2)_3NH_2$	4.3	0.7	0.2	5.8	1.3	8.8
**42**	4-F-C_6H_4	$(CH_2)_2C_6H_5$	$(CH_2)_3NH_2$	14.9	1.2	0.08	752.3	50.7	608.8
**43**	2-Naph	$(CH_2)_2C_6H_5$	$(CH_2)_3NH_2$	90.5	0.6	0.007	48.3	0.5	78.0
**44**	$(CH_2)_2NH_2$	$(CH_2)_2C_6H_5$	$(CH_2)_3NH_2$	0.2	0.5	3.2	13.4	90.0	28.1
**45**	$CH_2C_6H_5$	$(CH_2)_5CH_3$	$(CH_2)_5CH_3$	2.8	0.09	0.03	6.9	2.4	78.5
**46**	Fu	$(CH_2)_2C_6H_5$	$(CH_2)_2COOH$	33.1	0.2	0.007	11.2	0.3	47.3
**47**	4-F-C_6H_4	$(CH_2)_2C_6H_5$	$(CH_2)_2COOH$	4.1	0.2	0.06	10.9	2.7	47.6
**48**	2-Naph	$(CH_2)_2C_6H_5$	$(CH_2)_2COOH$	2.7	0.3	0.1	29.0	10.7	100.1
**49**	CH_2COOH	$(CH_2)_2C_6H_5$	$(CH_2)_2COOH$	0.5	6.2	12.8	28.8	59.2	4.6
**50**	Fu	$(CH_2)_5CH_3$	$(CH_2)_5CH_3$	5.6	0.4	0.07	134.2	24.0	328.6

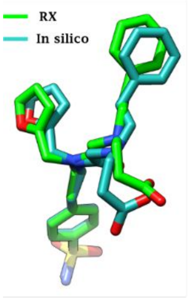

CA II

Figure S1. Superimposition of crystallographic/predicted binding orientations of compound **46** in CA II active site.

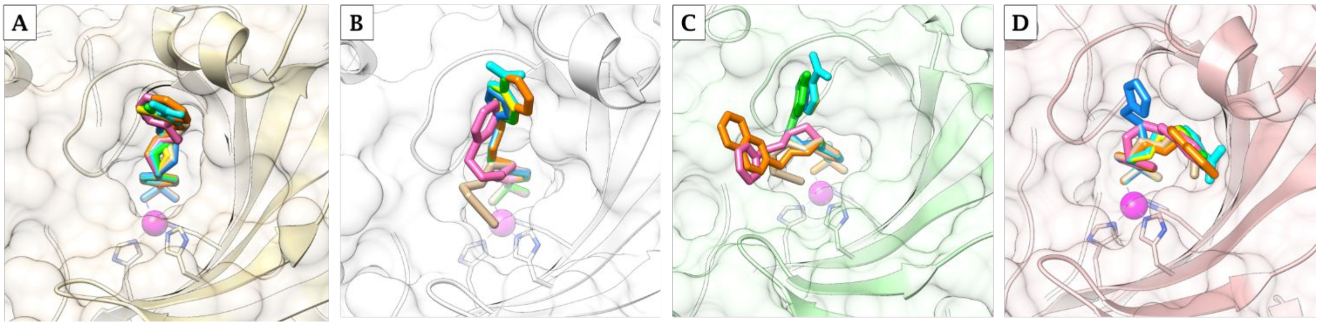

Figure S2. Superimpostion of docked orientations of **1** (yellow), **2** (cyan), **3** (green), **4** (orange), **5** (blue), **6** (tan), **7** (pink) within the active site of A) CA I, B) CA II, C) CA VI and D) CA XII.

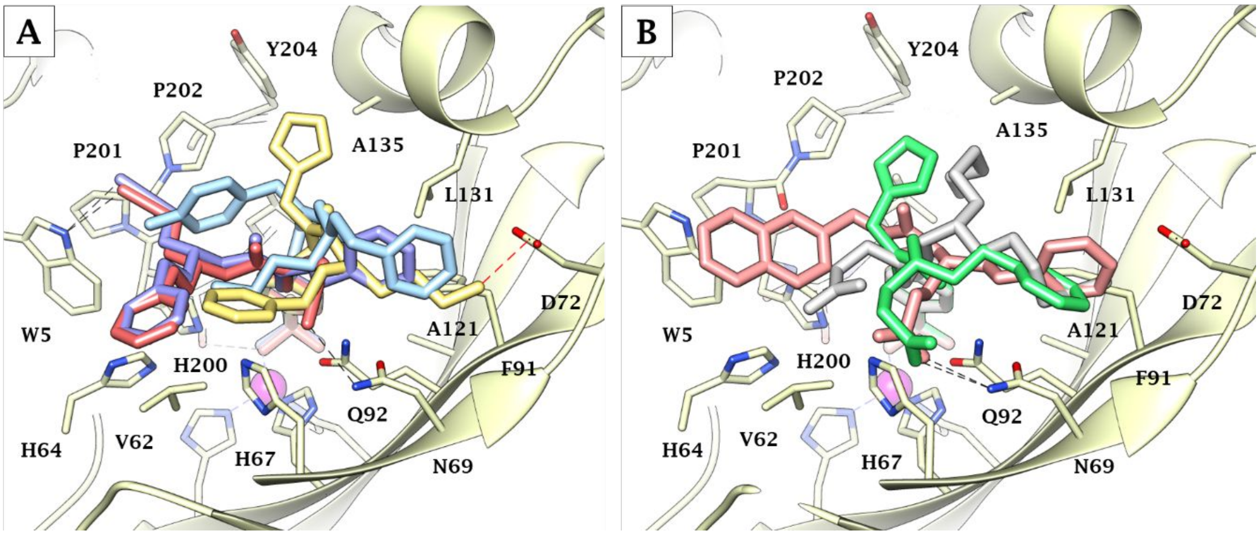

Figure S3. *In silico* predicted binding conformations of A) **34** (light purple), **39** (light red), **41** (light yellow), **42** (light blue) and B) **46** (green), **48** (peach), **49** (light grey) in CA I active site. H-bond and salt bridge interactions are depicted as black, and red dashed lines, respectively. Amino acids are

labelled with one letter symbols: A, Ala; D, Asp; E, Glu; F, Phe; H, His; I, Ile; K, Lys; L, Leu; M, Met; N, Asn; P, Pro; Q, Gln; T, Thr; V, Val; W, Trp; Y, Tyr.

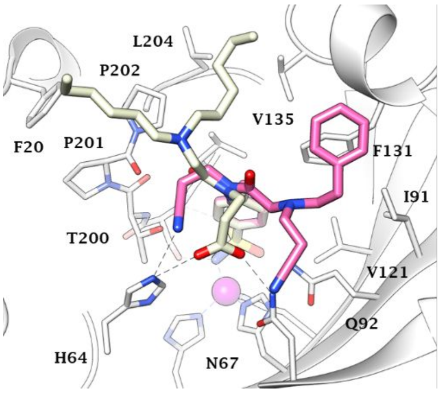

Figure S4. *In silico* predicted binding conformations of **39** (fuchsia) and **49** (white) in CA II active site. H-bond and salt bridge interactions are depicted as black, and red dashed lines, respectively.

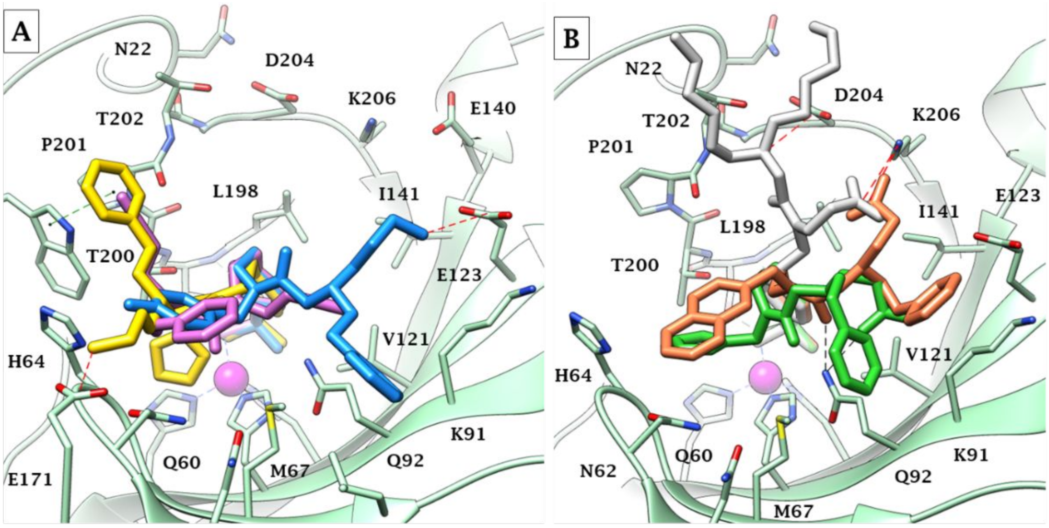

Figure S5. *In silico* predicted binding conformations of A) **39** (magenta), **41** (yellow), **42** (blue) and B) **46** (green), **48** (orange), **49** (light grey) in CA IV active site. H-bond, π-π and salt bridge interactions are depicted as black, green and red dashed lines, respectively.

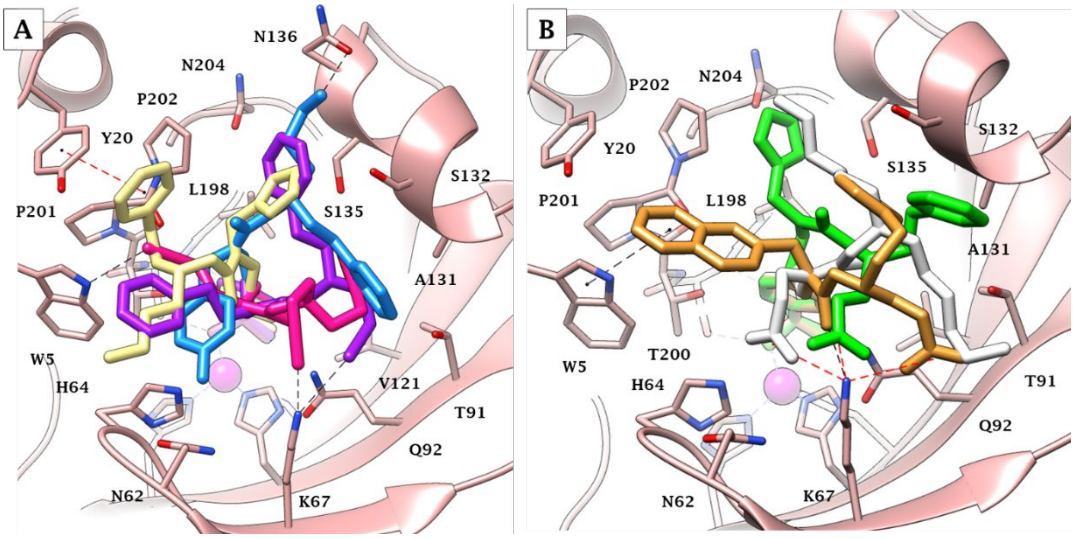

Figure S6. *In silico* predicted binding conformations of A) **34** (purple), **39** (magenta), **41** (light yellow), **42** (blue) and B) **46** (green), **48** (orange), **49** (white) in CA XII active site. H-bond, π-π and salt bridge interactions are depicted as black, green and red dashed lines, respectively.

The chromatographic profiles of LC-DAD analysis of compounds **18-50** are reported in Figures S7-S39.

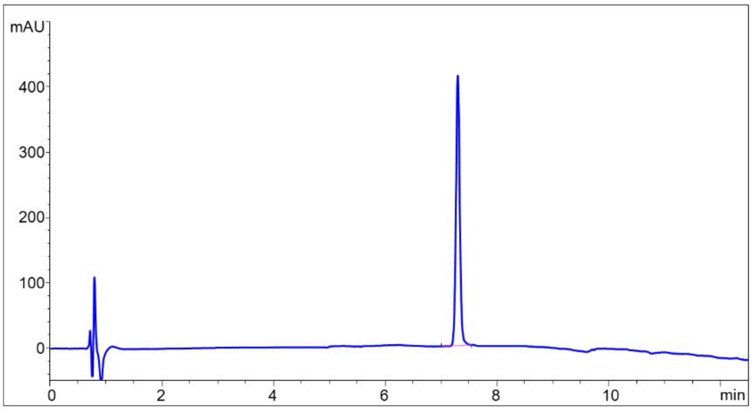

Figure S7. Chromatographic profile of **18** monitored at λ=230 nm.

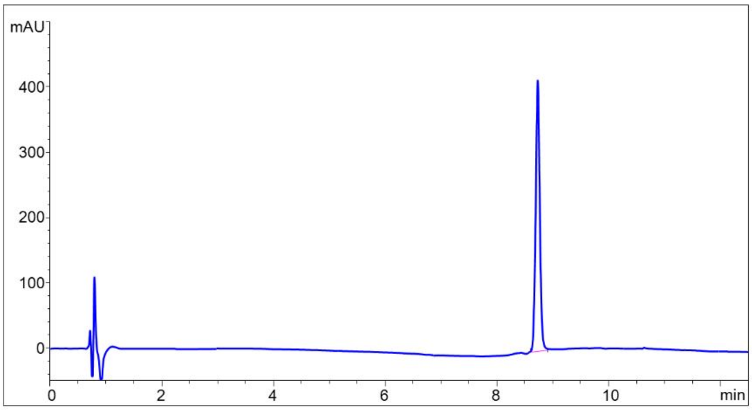

Figure S8. Chromatographic profile of **19** monitored at λ=230 nm.

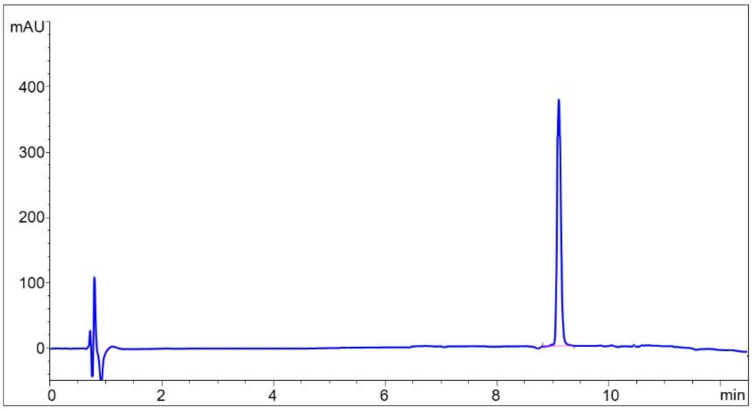

Figure S9. Chromatographic profile of **20** monitored at λ=230 nm.

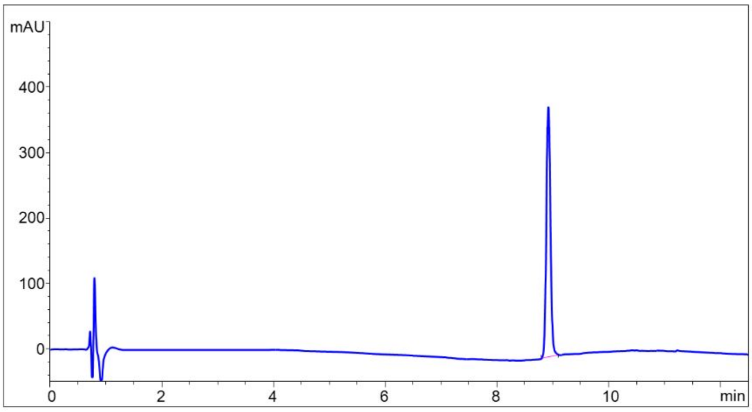

Figure S10. Chromatographic profile of **21** monitored at λ=230 nm.

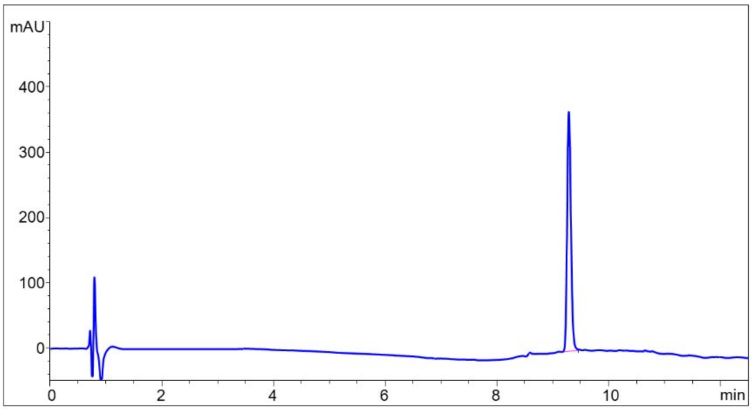

Figure S11. Chromatographic profile of **22** monitored at λ=230 nm.

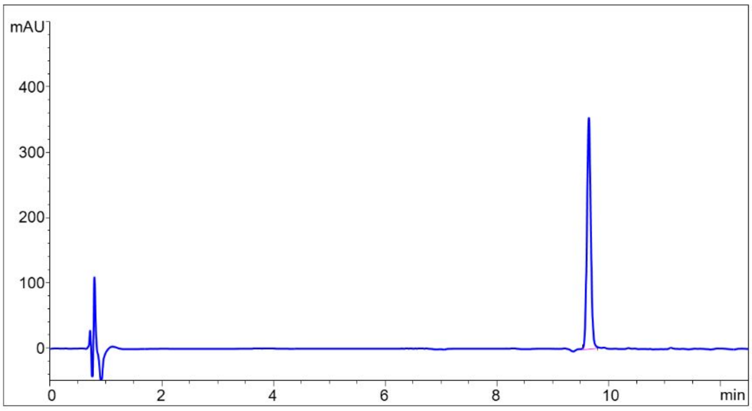

Figure S12. Chromatographic profile of **23** monitored at λ=230 nm.

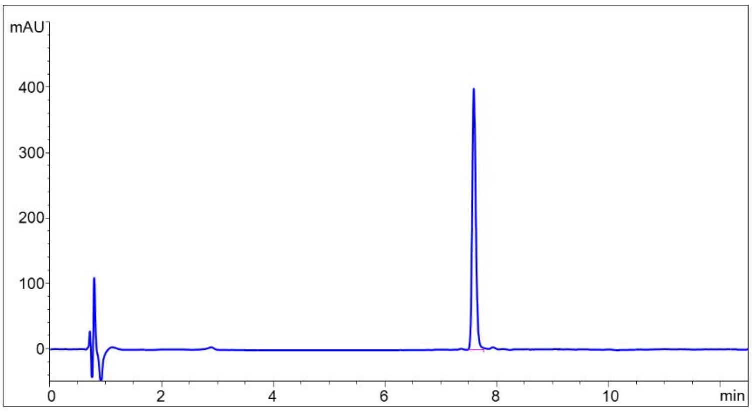

Figure S13. Chromatographic profile of **24** monitored at λ=230 nm.

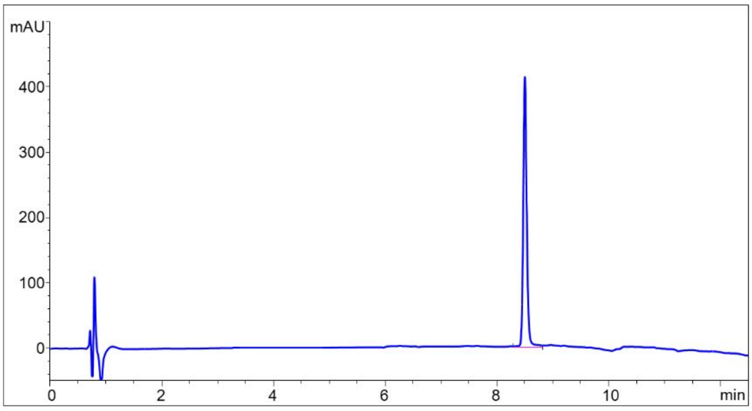

Figure S14. Chromatographic profile of **25** monitored at λ=230 nm.

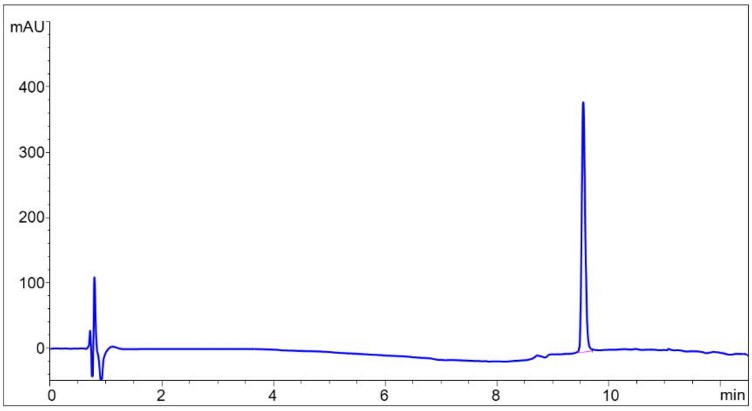

Figure S15. Chromatographic profile of **26** monitored at λ=230 nm.

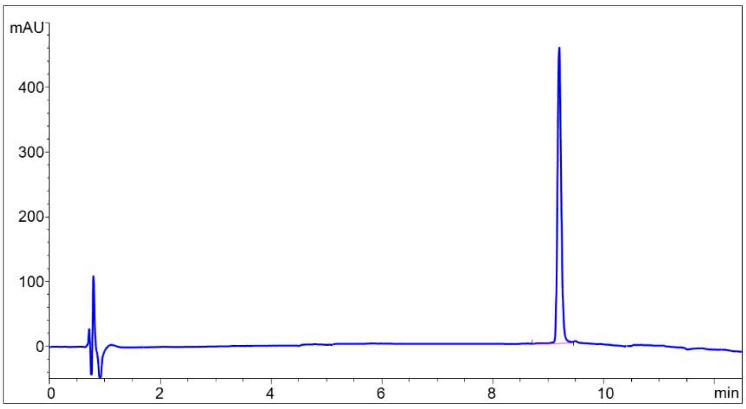

Figure S16. Chromatographic profile of **27** monitored at λ=230 nm.

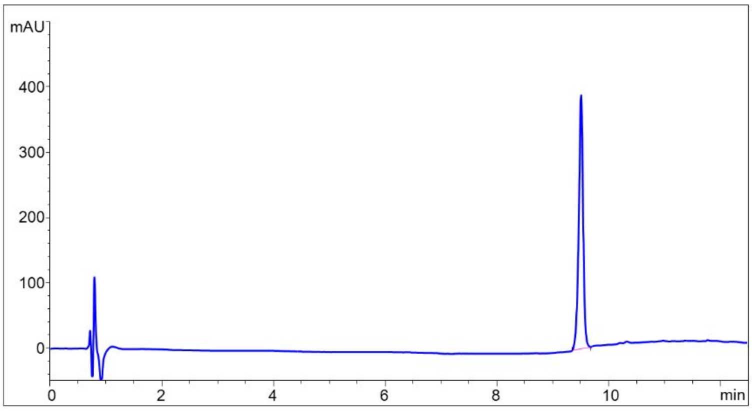

Figure S17. Chromatographic profile of **28** monitored at λ=230 nm.

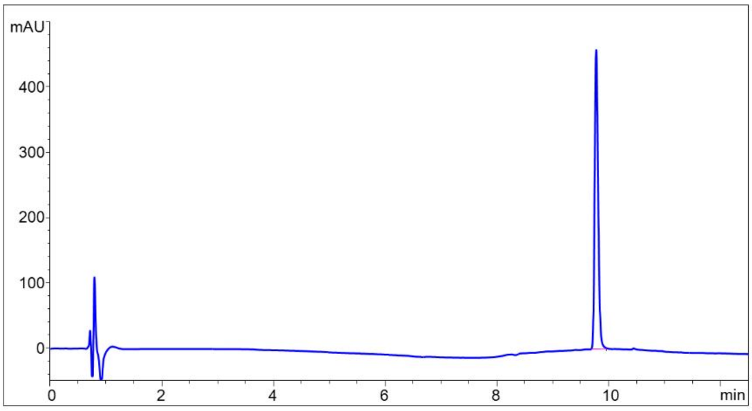

Figure S18. Chromatographic profile of **29** monitored at λ=230 nm.

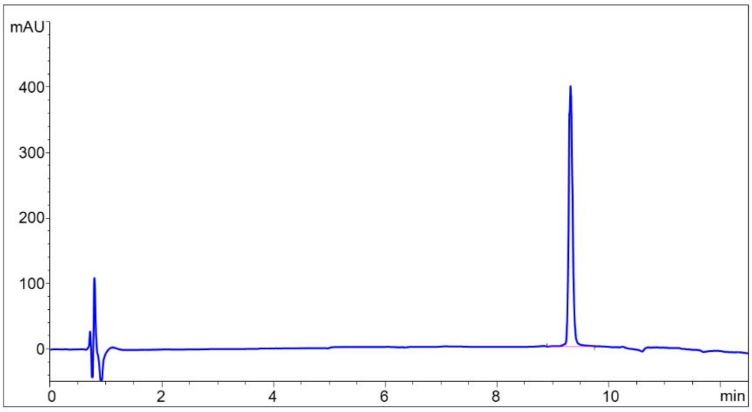

Figure S19. Chromatographic profile of **30** monitored at λ=230 nm.

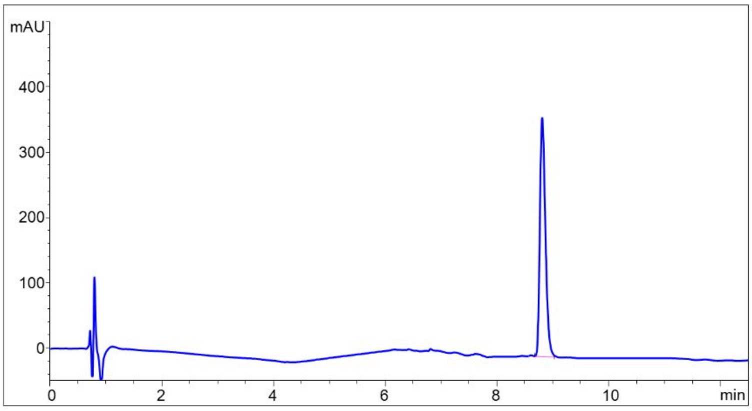

Figure S20. Chromatographic profile of **31** monitored at λ=230 nm.

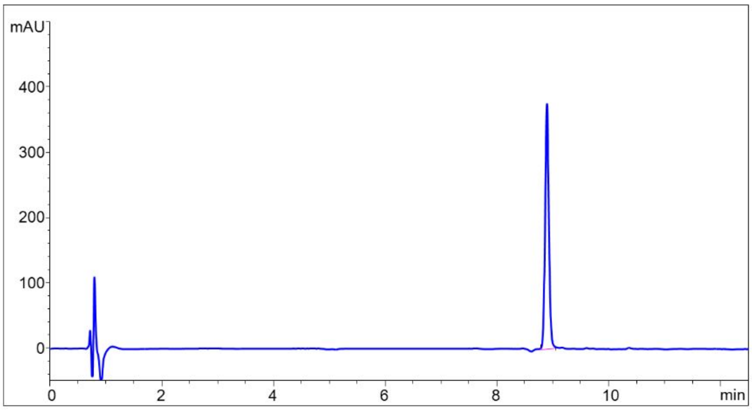

Figure S21. Chromatographic profile of **32** monitored at λ=230 nm.

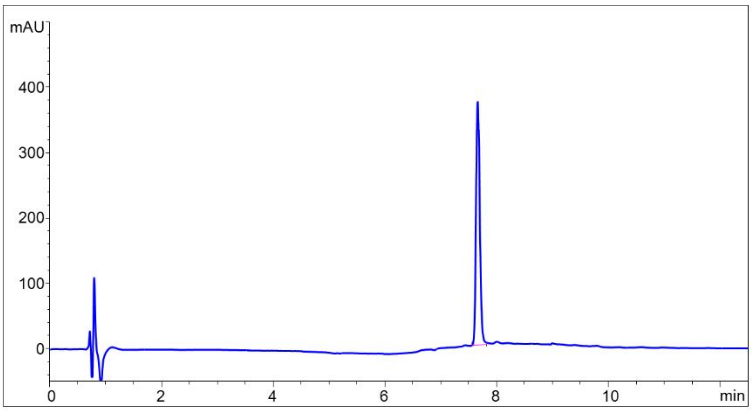

Figure S22. Chromatographic profile of **33** monitored at λ=230 nm.

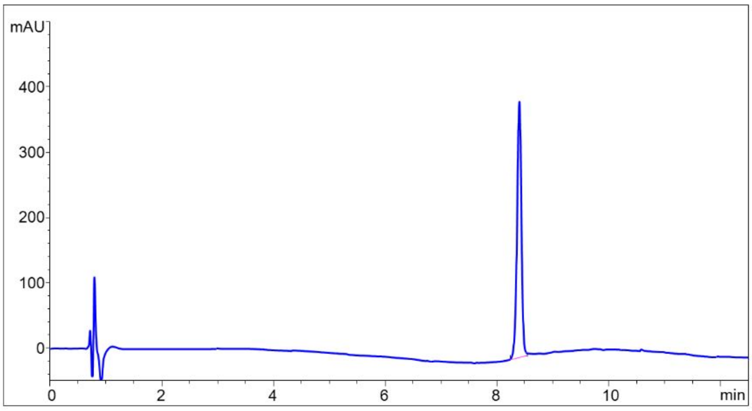

Figure S23. Chromatographic profile of **34** monitored at λ=230 nm.

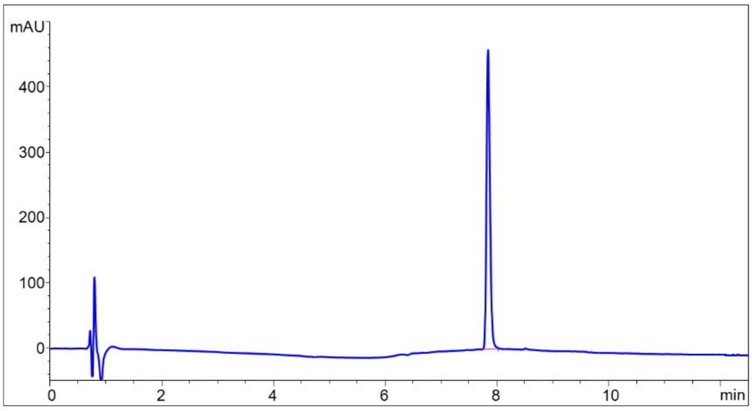

Figure S24. Chromatographic profile of **35** monitored at λ=230 nm.

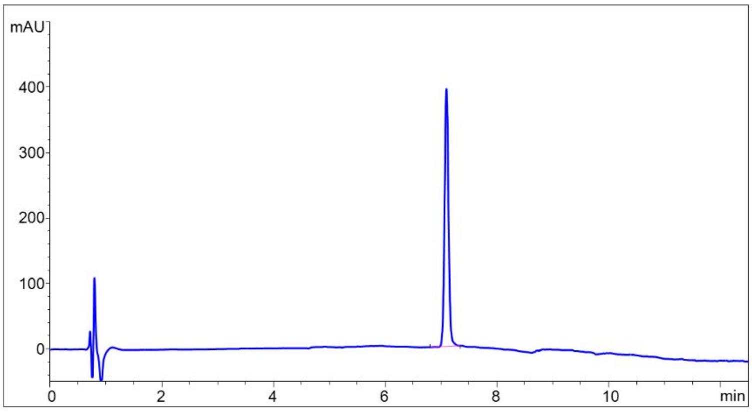

Figure S25. Chromatographic profile of **36** monitored at λ=230 nm.

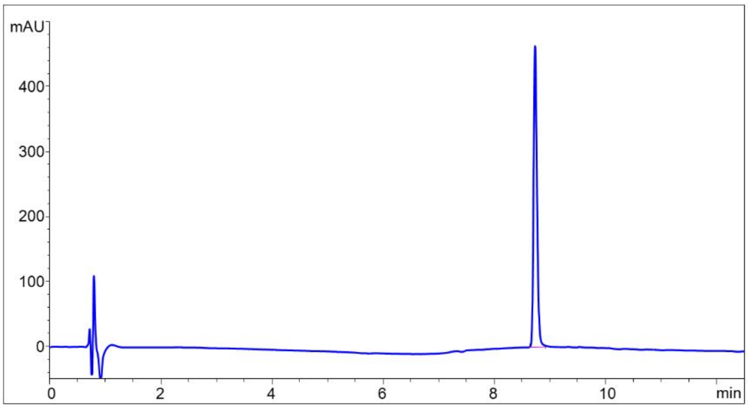

Figure S26. Chromatographic profile of **37** monitored at λ=230 nm.

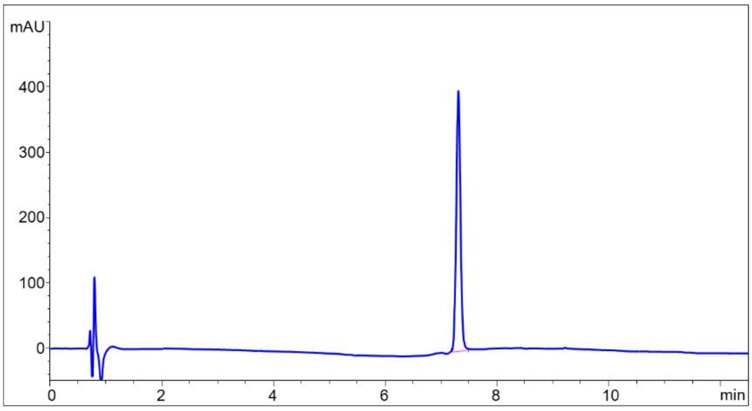

Figure S27. Chromatographic profile of **38** monitored at λ=230 nm.

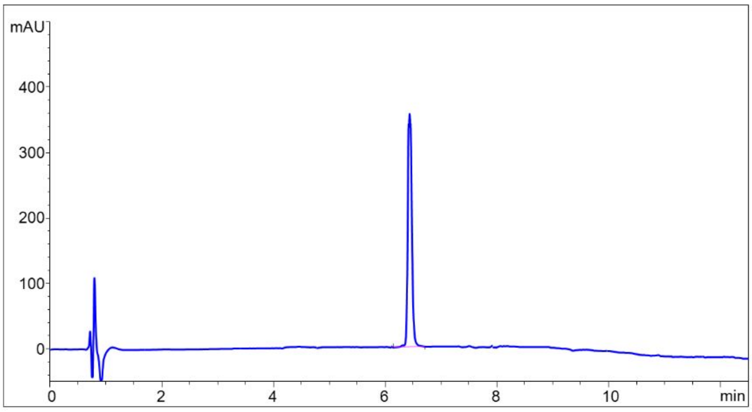

Figure S28. Chromatographic profile of **39** monitored at λ=230 nm.

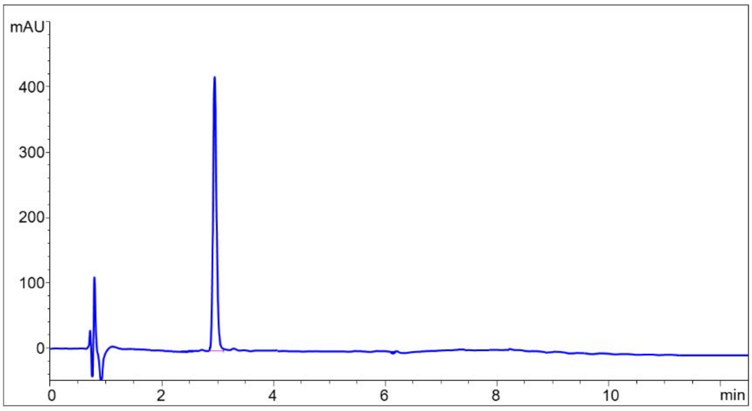

Figure S29. Chromatographic profile of **40** monitored at λ=230 nm.

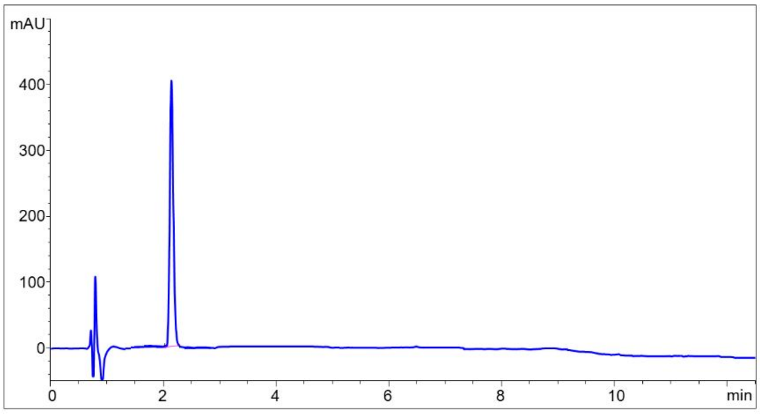

Figure S30. Chromatographic profile of **41** monitored at λ=230 nm.

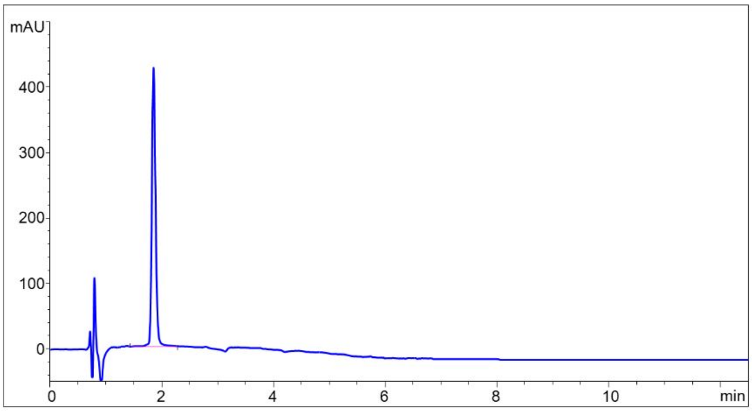

Figure S31. Chromatographic profile of **42** monitored at λ=230 nm.

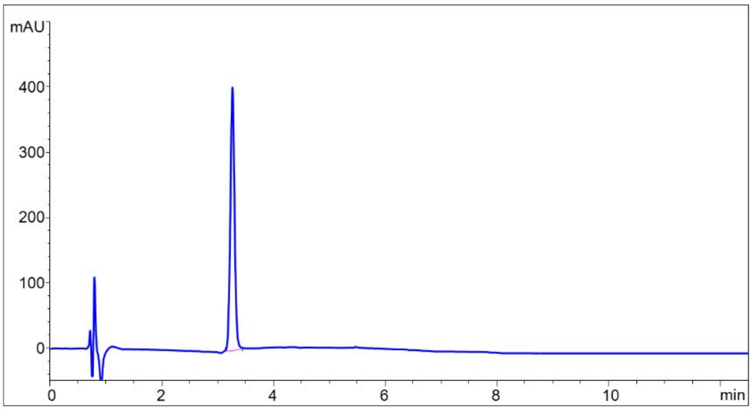

Figure S32. Chromatographic profile of **43** monitored at λ=230 nm.

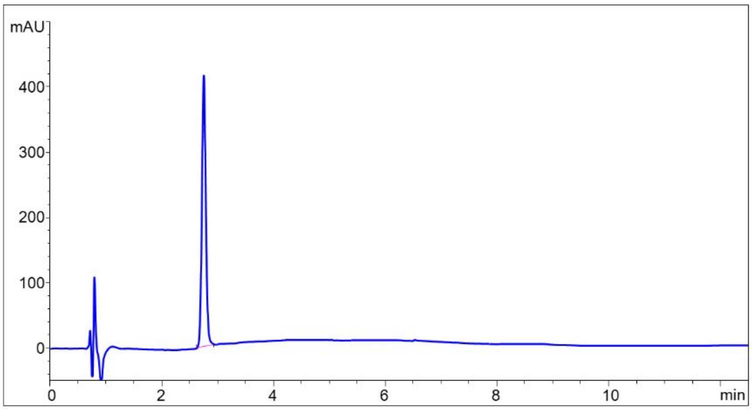

Figure S33. Chromatographic profile of **44** monitored at λ=230 nm.

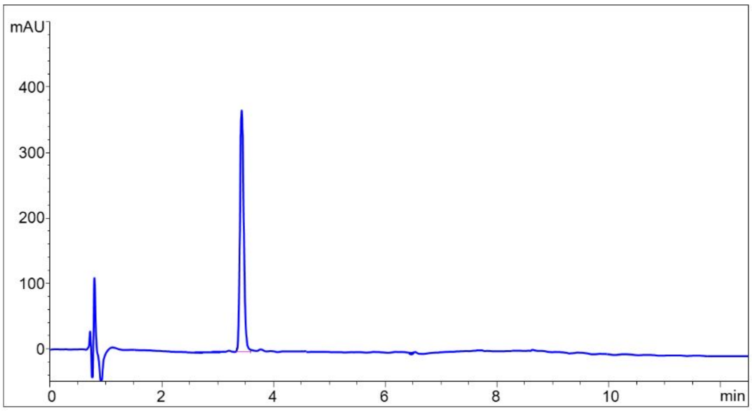

Figure S34. Chromatographic profile of **45** monitored at λ=230 nm.

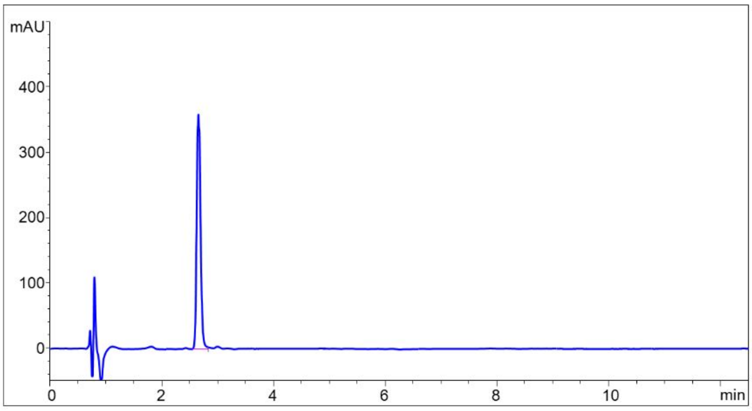

Figure S35. Chromatographic profile of **46** monitored at λ=230 nm.

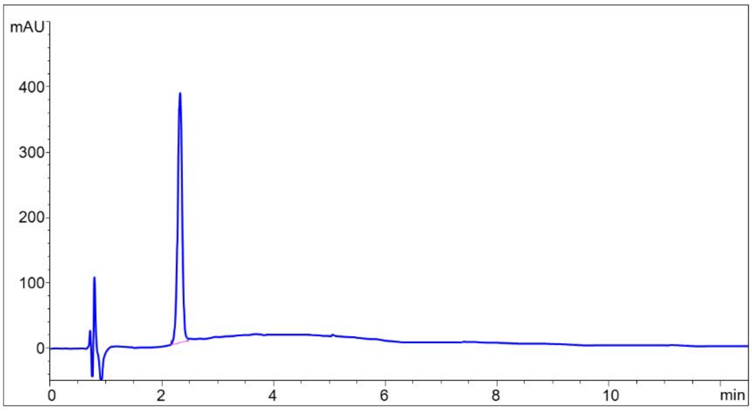

Figure S36. Chromatographic profile of **47** monitored at λ=230 nm.

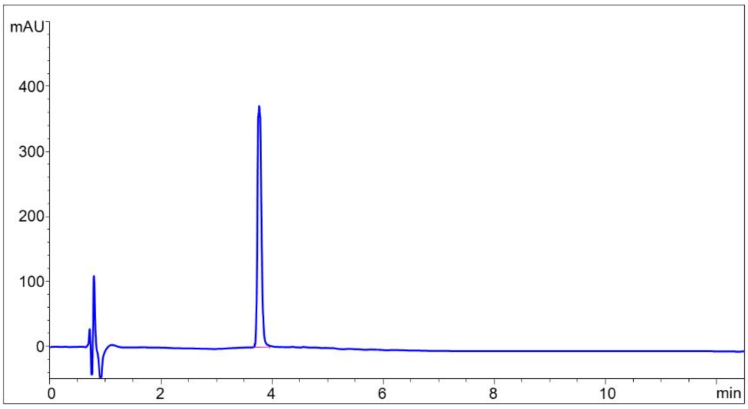

Figure S37. Chromatographic profile of **48** monitored at λ=230 nm.

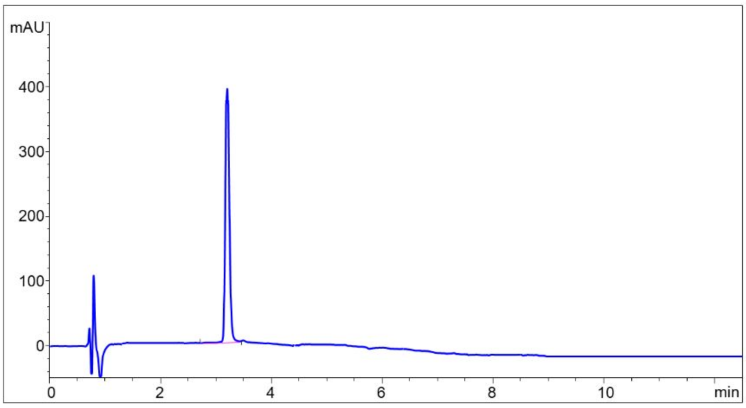

Figure S38. Chromatographic profile of **49** monitored at λ=230 nm.

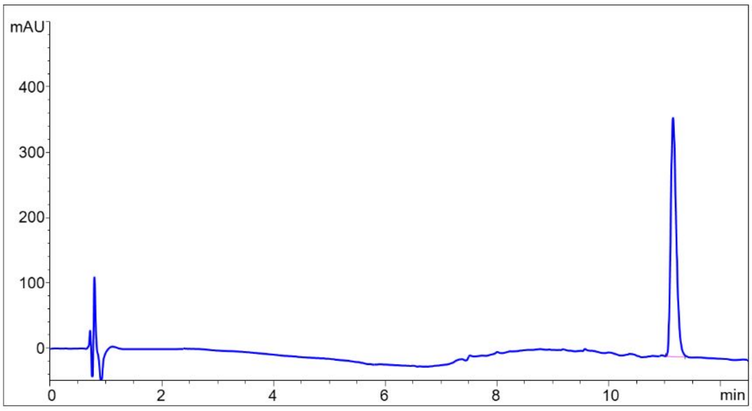

Figure S39. Chromatographic profile of **50** monitored at λ=230 nm.
